# An Osteoimmunomodulatory Biopatch Potentiates Stem Cell Therapies for Bone Regeneration by Simultaneously Regulating IL‐17/Ferroptosis Signaling Pathways

**DOI:** 10.1002/advs.202401882

**Published:** 2024-07-18

**Authors:** Shan Liu, Wenle Wang, Zhiyu Chen, Peng Wu, Wendan Pu, Gang Li, Jinlin Song, Jianxiang Zhang

**Affiliations:** ^1^ Chongqing Key Laboratory of Oral Diseases and Biomedical Sciences Chongqing Municipal Key Laboratory of Oral Biomedical Engineering of Higher Education Stomatological Hospital of Chongqing Medical University Chongqing Medical University Chongqing 401147 P. R. China; ^2^ Department of Pharmaceutics College of Pharmacy Third Military Medical University (Army Medical University) Chongqing 400038 P. R. China; ^3^ Department of Orthodontics II Affiliated Stomatological Hospital of Zunyi Medical University Zunyi 563000 P. R. China; ^4^ Department of Orthopedics The First Affiliated Hospital of Chongqing Medical University Chongqing 400016 P. R. China; ^5^ College of Pharmacy and Medical Technology Vocational and Technical College Hanzhong Shaanxi 723000 P. R. China; ^6^ Department of Stomatology Southwest Hospital Third Military Medical University (Army Medical University) Chongqing 400038 P. R. China; ^7^ State Key Laboratory of Trauma and Chemical Poisoning Third Military Medical University (Army Medical University) Chongqing 400038 P. R. China; ^8^ Yu‐Yue Pathology Scientific Research Center 313 Gaoteng Avenue, Jiulongpo Chongqing 400039 P. R. China

**Keywords:** bioactive patch, bone defect, nanofiber, osteoimmunity, stem cell therapy, tissue repair

## Abstract

Currently, there are still great challenges in promoting bone defect healing, a common health problem affecting millions of people. Herein an osteoimmunity‐regulating biopatch capable of promoting stem cell‐based therapies for bone regeneration is developed. A totally biodegradable conjugate is first synthesized, which can self‐assemble into bioactive nano micelles (PPT NMs). This nanotherapy effectively improves the osteogenesis of periodontal ligament stem cells (PDLSCs) under pathological conditions, by simultaneously regulating IL‐17 signaling and ferroptosis pathways. Incorporation of PPT NMs into biodegradable electrospun nanofibers affords a bioactive patch, which notably improves bone formation in two rat bone defect models. A Janus bio patch is then engineered by integrating the bioactive patch with a stem cell sheet of PDLSCs. The obtained biopatch shows additionally potentiated bone regeneration capacity, by synergistically regulating osteoimmune microenvironment and facilitating stem cell differentiation. Further surface functionalization of the biopatch with tannic acid considerably increases its adhesion to the bone defect, prolongs local retention, and sustains bioactivities, thereby offering much better repair effects in rats with mandibular or cranial bone defects. Moreover, the engineered bioactive patches display good safety. Besides bone defects, this osteoimmunity‐regulating biopatch strategy can be applied to promote stem cell therapies for spinal cord injury, wound healing, and skin burns.

## Graphical abstract

A Janus biopatch is engineered by integrating a nanofibrous membrane containing an inflammation‐resolving nanotherapy and a stem cell sheet. Synergistic osteoimmunomodulatory effects and stem cell therapies for bone regeneration are achieved and demonstrated in rats with mandibular and cranial defects, by simultaneously regulating IL‐17/ferroptosis signaling. This bioactive bilayer strategy is promising for potentiating stem cells to treat inflammatory bone diseases.

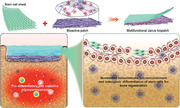

## 1 Introduction

Bone defects, caused by a range of factors, including trauma, surgery, infection, and tumors, affect millions of people worldwide.^[^ ^1^ ^]^ Whereas autogenous and allogenic bone grafts remain gold standard treatments for bone defects in clinics,^[^ ^2^ ^]^ these options have significant drawbacks, such as limited availability, the need for additional surgery, donor site morbidity, immunological and inflammatory complications at the implantation site, and size mismatch.^[^ ^3^ ^]^ To overcome these limitations, various bone tissue engineering strategies, like biomaterial‐based substitutes or scaffolds, stem cell therapies, and different types of molecular therapeutics (e.g., growth factors and nucleic acids) as well as their combinations have been examined to promote bone defect repair.^[^ ^4^ ^]^ In particular, stem cell‐based therapies have emerged as a promising strategy for bone repair, owing to their multiple advantages, such as self‐renewal capacity, multilineage differentiation potential, and paracrine effects. However, direct injection of stem cells into the target tissues will lead to decreased cell viability, short‐time retention, undesirable engraftment, and impaired differentiation, thus limiting their practical applications. Consequently, different types of natural and/or synthetic biomaterials have been examined for stem cell delivery, such as chitosan, hyaluronic acid, alginate, cellulose, collagen, gelatin, and polyesters, in the forms of microparticles, hydrogels, or other delicately engineered structures.^[^ ^5^ ^]^ In addition, to avoid drawbacks of tissue regeneration based on various scaffolds or single cell suspensions, 2/3D cell sheets and bone organoids have been proposed as alternative strategies, for various tissue reconstructions, including the bone, corneal endothelium, periodontal tissue, blood vessel, cardiac tissue, bladder, and liver.^[^ ^6^ ^]^

Despite these achievements, most of the available delivery systems largely provide physical microenvironments for stem cell attachment, proliferation, and differentiation, and their tissue regeneration capabilities under many pathological conditions are considerably compromised. As well recognized, tissue repair after injury is a complex process that is closely related to the regenerative capacity of the injured tissue and the quality of inflammatory responses.^[^ ^7^ ^]^ Bone defects resulting from different factors are commonly accompanied by inflammation. Indeed, delicately modulated inflammation is beneficial for eradicating the injurious source, initiating molecular/cellular cascades for tissue repair and regeneration, and restoring normal tissue architecture and function.^[^ ^7^, ^8^ ^]^ However, if this well‐orchestrated tissue‐regenerative response is perturbed by uncontrolled and sustained inflammatory and immune responses, tissue homeostasis will not be re‐established. Previous studies showed that severe inflammation can hamper the regeneration process and promote the progression of bone defects, especially inflammatory bone diseases.^[^ ^9^ ^]^ For instance, excessive neutrophil infiltration and activation can amplify inflammatory cascades and impair the repair process.^[^ ^10^ ^]^ In addition, many studies indicated that the balance of regulatory T (Treg) and T helper 17 (Th17) cells is important for the maintenance of bone homeostasis.^[^ ^11^ ^]^ In inflammatory bone diseases, an excess of Th17 cells, due to Treg/Th17 cell imbalance, mainly contributes to pathological bone metabolism. Besides, excess reactive oxygen species (ROS) accompanied by inflammation can result in oxidative damage, induce apoptosis of bone cells (such as osteocytes and osteoblasts), and attenuate osteogenic differentiation of stem cells, thus impeding bone regeneration.^[^ ^2^, ^12^ ^]^ Therefore, various therapeutic approaches capable of regulating the osteoimmune microenvironment have been examined to treat bone diseases, involving the use of glucocorticoids, nonsteroid anti‐inflammatory drugs, and biologic agents like antibodies to interleukin (IL)−6 and tumor necrosis factor (TNF)‐α.^[^ ^13^ ^]^ Unfortunately, depending on the dosage and treatment period, these anti‐inflammatory agents can cause gastrointestinal disorders,^[^ ^14^ ^]^ acute liver/kidney injury,^[^ ^15^ ^]^ cardiovascular complications,^[^ ^16^ ^]^ osteoporosis,^[^ ^17^ ^]^ and the increased risk of malignancies and infections.^[^ ^18^ ^]^ Consequently, novel potent and safe anti‐inflammatory strategies remain to be discovered. On the other hand, it is highly necessary to rationally design delivery systems to efficiently and simultaneously load anti‐inflammatory drugs and stem cells as well as programmatically and synergistically regulate the osteoimmune microenvironment for highly effective bone tissue regeneration.

**Figure 1:**
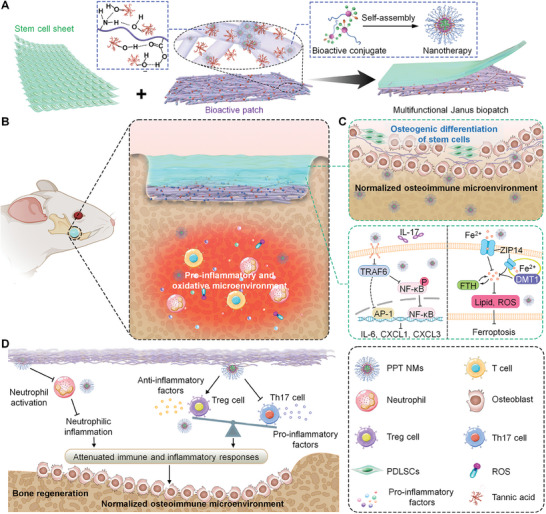
Schematic illustration of engineering a multifunctional Janus biopatch for bone regeneration. A) A sketch showing the structure, compositions, and development of the multifunctional Janus biopatch. The Janus biopatch consists of an inflammation‐resolving membrane and a stem cell sheet derived from periodontal ligament stem cells (PDLSCs). In the bioactive membrane, an anti‐inflammatory nanotherapy PPT NMs assembled by an amphiphilic conjugate is embedded in electrospun nanofibers, which are further functionalized with tannic acid (TA). B) Schematic of the treatment of a bone defect with the Janus biopatch. The bioactive membrane is planted onto the interior side, while the cell sheet is located outside. C) Cellular and molecular mechanisms underlying bone regeneration effects of PDLSCs in the Janus biopatch. The upper panel shows the gradual release of the nanotherapy concomitant with degradation of the fibrous membrane, thus affording an improved pathological microenvironment and facilitating differentiation of PDLSCs into osteoblasts in the bone defect. Meanwhile, degradation of the fibrous membrane enables infiltration of osteoblasts into the interior of the Janus biopatch to promote bone regeneration. The low panel illustrates signaling pathways underscoring bioactivities of PPT NMs in PDLSCs. D) A diagram indicates the regulation of the osteoimmune (immune‐inflammatory) microenvironment by PPT NMs to promote bone regeneration.

To address the above‐mentioned challenges in bone tissue regeneration, here we propose an osteoimmunity‐regulating strategy to promote stem cell therapies. In this aspect, a bilayer patch integrated with a bioactive nanofibrous membrane and a stem cell sheet was designed. The bioactive membrane, constructed by incorporating intrinsically anti‐inflammatory nanomicelles (NMs) into electrospun nanofibers based on biodegradable and biocompatible materials, enables sustained release of an inflammation‐relieving nanotherapy, which in turn facilitates osteogenesis of stem cells by regulating the pathological osteoimmune microenvironment and reshaping the stem cell niche (**Figure** **1**). The cell sheet is engineered using periodontal ligament stem cells (PDLSCs) that play critical roles in periodontal tissue regeneration. Furthermore, this Janus biopatch was functionalized with a natural polyphenol to enhance its tissue adhesion and prolong retention time, thus sustaining biological activities. The effectiveness of this engineered multifunctional Janus patch was fully examined in rats with mandibular or cranial defects, i.e., two typical models of inflammation‐related bone defects.

## 2 Result

### 2.1 Preparation, Characterization, and Biological Evaluations of Inflammation‐Resolving Nanomicelles

First, we developed a bioactive and amphiphilic conjugate (defined as PPT) to engineer inflammation‐resolving NMs. PPT was synthesized by sequentially conjugating three functional modules, i.e., polyethylene glycol (PEG), Tempol (TP), and phenylboronic acid pinacol ester (PBE), onto a hydrolyzable hexachlorocyclotriphosphazene (HCCP) scaffold (Figure S1A, Supporting Information). Of note, PEG can provide hydrophilicity for the conjugate and afford colloidal stability for resulting NMs, while TP functions as a superoxide dismutase‐mimetic unit capable of eliminating superoxide anion and free radicals. PBE is a ROS‐responsive/scavenging moiety, and its ROS‐triggered hydrolysis can generate p‐(hydroxymethyl) phenol (HMP), an anti‐inflammatory agent. Importantly, PBE may initiate hydrolysis of PPT in the presence of hydrogen peroxide (H_2_O_2_), thus offering biodegradability for PPT.

**Figure 2:**
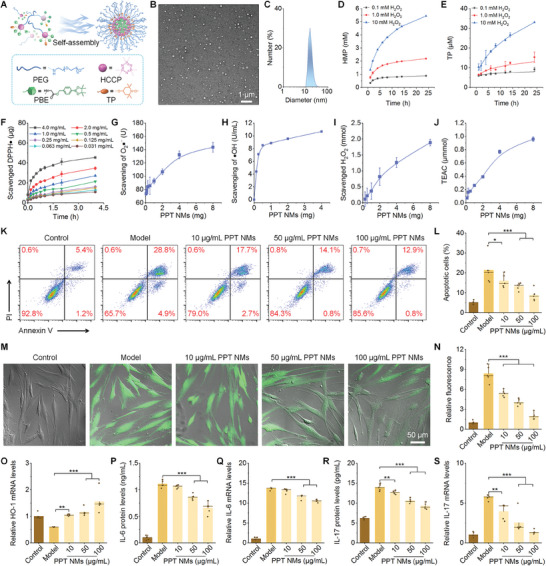
Engineering of inflammation‐resolving nanomicelles (NMs) and evaluation of the biological effects. A) Schematic showing self‐assembly of bioactive NMs by an amphiphilic conjugate PPT. B,C) TEM image B) and size distribution C) of PPT NMs. D,E) ROS‐triggered time‐ and dose‐dependent release of HMP and TP from PPT NMs. F) Time‐ and dose‐dependent scavenging of DPPH radical by PPT NMs. G–I) Dose‐dependent elimination of superoxide anion G), hydroxyl radical H), and H_2_O_2_ I). J) Trolox‐equivalent antioxidant capacity (TEAC) of PPT NMs. K,L) Flow cytometric profiles and quantitative analysis of cell apoptosis of hPDLSCs treated with different formulations. M,N) Typical fluorescence images M) and quantified fluorescence intensities N) showing ROS generation in hPDLSCs after different treatments and stained with a fluorescent probe DCFH‐DA. O–S) mRNA levels of HO‐1 as well as mRNA and protein levels of IL‐6 P,Q) and IL‐17 R,S). Of note, 1 mg PPT is equivalent to 270 µmol PPT, while 100 µg mL^−1^ PPT NMs means 27 µmol L^−1^ PPT. hPDLSCs in the control group were treated with fresh medium, while the model group was induced with 10 µg mL^−1^ LPS for 24 h. For PPT NMs groups, cells were treated with 10 µg mL^−1^ LPS and various doses of PPT NMs for 24 h. Data are presented as means ± SD D–J, *n* = 3; K–S, *n* = 5). ^*^ *p* < 0.05, ^**^ *p* < 0.01, and ^***^ *p* < 0.001.

The successful synthesis of PPT was structurally confirmed by Fourier transform infrared (FTIR) and ^1^H NMR spectroscopy (Figure S1B,C, Supporting Information). According to the ^1^H NMR spectrum, each PPT molecule contained one PEG chain, one TP unit, and four PBE moieties. PPT was able to dissolve in water and common organic solvents, such as methanol, ethanol, acetone, and dichloromethane, thus affording excellent processability. Owing to its amphiphilicity, PPT can directly self‐assemble into NMs in an aqueous solution (**Figure** **2**A). Observation by transmission electron microscopy (TEM) indicated a nearly spherical shape for the assembled PPT NMs, with an average hydrodynamic diameter of 33 ± 2 nm and ζ‐potential of 5 ± 1 mV (Figure 2B,C).

PBE is highly sensitive to H_2_O_2_.^[^ ^19^ ^]^ In the presence of H_2_O_2_, the PBE unit will be first hydrolyzed and sequentially initiate hydrolysis of the HCCP scaffold. Complete hydrolysis of PPT generates totally water‐soluble byproducts, including PEG, pinacol, p‐(hydroxymethyl)phenol (HMP), TP, and glycine, as confirmed by electrospray ionization mass spectroscopy and ^1^H NMR spectrometry (Figure S1D–F, Supporting Information). Concomitant with PPT hydrolysis, HMP, and TP were gradually released, in both H_2_O_2_ concentration‐ and time‐dependent manners (Figure 2D,E; Figure S1G–I, Supporting Information). Notably, HMP displays desirable anti‐inflammatory and antiapoptotic effects, while TP is a free radical/superoxide anion scavenger with good anti‐oxidative activity.^[^ ^20^ ^]^ Therefore, ROS‐triggered hydrolysis of PPT enables the simultaneous release of two bioactive agents, which collectively deploy inflammation‐resolving capability. Correspondingly, in vitro tests showed that PPT NMs effectively eliminated 2,2‐diphenyl‐1‐picrylhydrazyl radical (DPPH•), superoxide anion (O_2_•^−^), hydroxyl radical (•OH), and H_2_O_2_, in dose‐response profiles (Figure 2F–I). Also, a total antioxidant capacity verified that PPT NMs exhibited excellent ROS‐scavenging capacity in a dose‐dependent manner (Figure 2J). These results demonstrated that PPT NMs can effectively scavenge multiple types of ROS.

As well documented, PDLSCs are essential for periodontal tissue regeneration, and their multi‐differentiation ability enables facilitated osteogenesis.^[^ ^21^ ^]^ However, severe inflammation can cause oxidative stress, cell apoptosis, and impaired stemness of PDLSCs.^[^ ^21^, ^22^ ^]^ Resolution of inflammation provides a favorable microenvironment for periodontal bone regeneration.^[^ ^21^, ^23^ ^]^ Consequently, we examined in vitro biological effects of PPT NMs in human PDLSCs (i.e., hPDLSCs). hPDLSCs were isolated from premolar extractions of adolescents, which were identified by flow cytometry (Figure S2, Supporting Information), showing high expressions of specific stem cell surface biomarkers including CD44 (99.7%), CD166 (98.8%), CD29 (99.7%), and CD73 (94.9%), but low expressions of CD45 (6.7%) and HLA‐DR (0.6%). After in vitro incubation of hPDLSCs with Cy5‐labeled PPT NMs (Cy5‐PPT NMs), confocal microscopic observation and flow cytometric quantification showed time‐ and dose‐dependent cellular internalization of Cy5‐PPT NMs in hPDLSCs (Figures S3 and S4, Supporting Information). Moreover, 3 days of incubation with various doses of PPT NMs showed no significant effects on cell proliferation of hPDLSCs (Figure S5, Supporting Information).

Then we used an acute inflammation model by inducing hPDLSCs with lipopolysaccharide (LPS) for 24 h to investigate whether PPT NMs possess antiapoptotic, antioxidative, and anti‐inflammatory activities. Whereas LPS mediated notable apoptosis of hPDLSCs, PPT NMs effectively mitigated LPS‐stimulated cell apoptosis, in a dose‐dependent manner (Figure 2K,L). Correspondingly, fluorescence observation and flow cytometric analysis suggested that PPT NMs significantly inhibited LPS‐induced intracellular ROS generation in hPDLSCs (Figure 2M,N; Figure S6A,B, Supporting Information). Consistently, PPT NMs effectively reduced ROS excretion in LPS‐induced hPDLSCs (Figure S6C). Interestingly, treatment with PPT NMs was able to reverse the reduced mRNA level of heme oxygenase‐1 (HO‐1) in LPS‐treated hPDLSCs (Figure 2O). Of note, HO‐1, an inducible enzyme capable of breaking heme, is a well‐recognized antioxidant.^[^ ^24^ ^]^ Accordingly, this result implicated that PPT NMs can also serve as a novel HO‐1 inducer, thus exerting additional indirect antioxidative effects by the HO‐1 pathway. Moreover, hPDLSCs induced with LPS for 24 h expressed significantly higher mRNA and protein levels of interleukin (IL)−6 and IL‐17, two typical pro‐inflammatory cytokines, while treatment with PPT NMs remarkably inhibited their expressions (Figure 2P–S).

Collectively, we successfully synthesized a bioactive amphiphile by integrating three different functional moieties onto a cyclic and labile scaffold, which can assemble into intrinsically multi‐bioactive nanotherapy PPT NMs. Upon ROS‐triggered hydrolysis, PPT NMs can efficiently scavenge multiple types of ROS, concomitant with the release of natural antioxidant and anti‐inflammatory compounds. Consistent with these results, cellular studies in hPDLSCs demonstrated that PPT NMs exhibited excellent antioxidant, anti‐inflammatory, and antiapoptotic activities. Consequently, PPT NMs can function as a potent nanotherapy for normalizing oxidative and pro‐inflammatory conditions.

### 2.2 PPT NMs Promote Osteogenic Differentiation of hPDLSCs under Inflammatory Conditions

**Figure 3:**
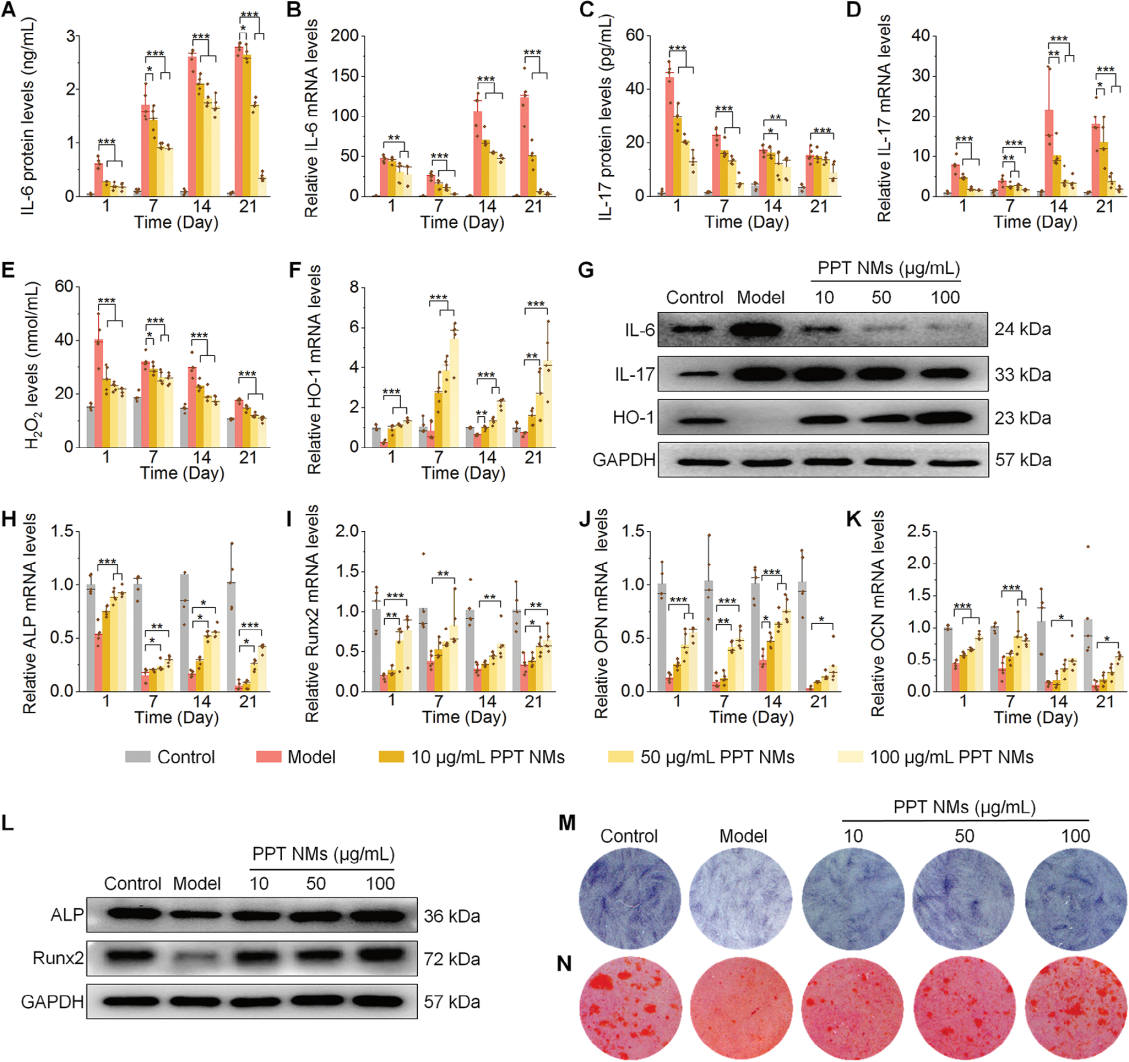
PPT NMs promote osteogenic capacity of hPDLSCs under chronic inflammatory conditions. A–D) mRNA and protein levels of typical pro‐inflammatory cytokines IL‐6 A,B) and IL‐17 C,D). E,F) Quantification of ROS secretion E) and mRNA levels of HO‐1 F). G) WB analysis of protein levels of IL‐6, IL‐17, and HO‐1 at day 7 after different treatments. H–K) mRNA levels of ALP H), Runx2 I), OPN J), and OCN K). L) Typical WB bands indicating protein levels of ALP and Runx2 at day 7. M,N) Digital photos show ALP activity and mineralized nodules of hPDLSCs after staining with ALP M) or ARS N) at days 7 and 21, respectively. In all groups, hPDLSCs were cultured in the osteogenic medium. In the control group, hPDLSCs were treated with the osteogenic medium alone, while the model group was induced with 10 µg mL^−1^ LPS. For PPT NMs groups, hPDLSCs were treated with 10 µg mL^−1^ LPS and various doses of PPT NMs for various time periods. Data are presented as means ± SD (*n* = 5). ^*^ *p* < 0.05, ^**^ *p* < 0.01, and ^***^ *p* < 0.001.

Based on the excellent inflammation resolution capability of PPT NMs, we then examined whether this nanotherapy can rescue impaired osteogenic differentiation of hPDLSCs under inflammatory conditions. To this end, sustained LPS exposure was employed to simulate chronic inflammation. LPS‐treated hPDLSCs showed significantly high mRNA/protein levels of pro‐inflammatory cytokines IL‐6 and IL‐17, compared with the normal cells at days 1, 7, 14, and 21 after different treatments (**Figure** **3**A–D). By contrast, PPT NMs demonstrated dose‐dependent anti‐inflammatory effects, showing significantly reduced IL‐6 and IL‐17 in hPDLSCs treated with PPT NMs. Also, LPS‐induced ROS excretion was significantly inhibited and the HO‐1 expression was dramatically increased at predefined time points after intervention with PPT NMs in this cellular model of chronic inflammation (Figure 3E,F). The antioxidant and anti‐inflammatory activities of PPT NMs were further confirmed by western blot (WB) analysis on day 7 after different treatments (Figure 3G; Figure S7A, Supporting Information). In particular, PPT NMs at 100 µg mL^−1^ exhibited the best antioxidant and anti‐inflammatory effects. This is in line with the results obtained in the acute inflammation model (Figure 2K–S).

On the other hand, LPS‐treated hPDLSCs showed significantly lower levels of bone formation‐related genes like alkaline phosphatase (ALP), Runx family transcription factor 2 (Runx2), osteopontin (OPN), and osteocalcin (OCN), as compared to the control group treated with the osteogenic medium alone (Figure 3H–K). By contrast, PPT NMs‐treated hPDLSCs displayed considerably increased mRNA levels of ALP, Runx2, OPN, and OCN, with the best pro‐osteogenic effect for PPT NMs at 100 µg mL^−1^, which was further confirmed by WB analysis (Figure 3L; Figure S7B, Supporting Information). Furthermore, direct observation and quantitative analysis of ALP‐stained hPDLSCs at day 7, as well as alizarin red S (ARS)‐stained hPDLSCs at day 21, verified that PPT NMs‐treated hPDLSCs displayed notably enhanced osteogenic activity and much more mineralized nodules under chronic inflammatory conditions (Figure 3M,N; Figure S7C,D, Supporting Information). Taken together, these results indicated that PPT NMs can effectively rescue the impaired osteogenic differentiation of hPDLSCs in a chronic inflammatory environment. The pro‐osteogenic effect of PPT NMs is mainly attributed to the antioxidative and anti‐inflammatory activities.

### 2.3 PPT NMs Relieve Inflammatory Responses in hPDLSCs by Regulating the IL‐17 Signaling and Ferroptosis Pathways

**Figure 4:**
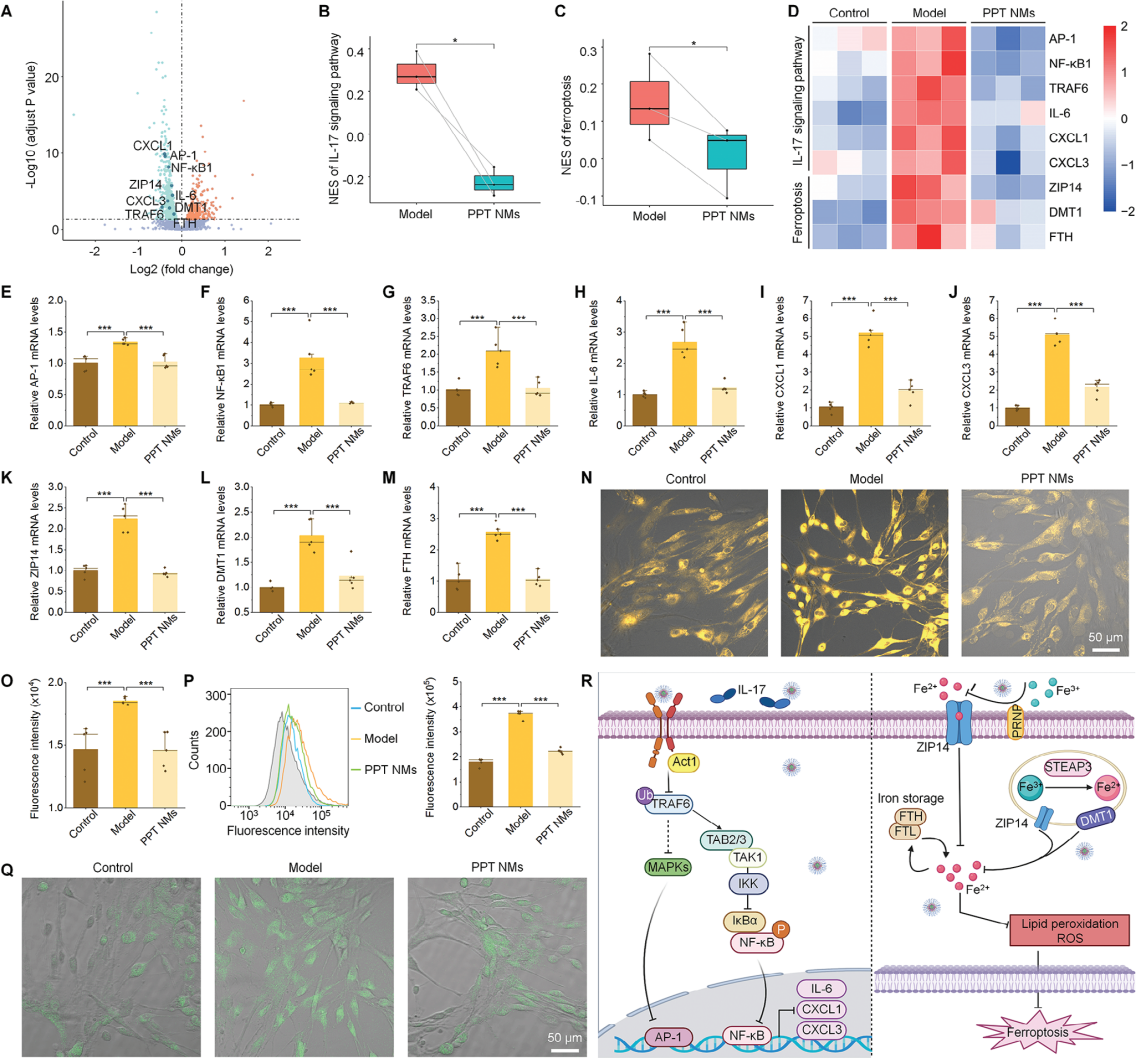
PPT NMs rescue hPDLSCs from pathological environment‐induced abnormal cellular stress responses via IL‐17 signaling and ferroptosis pathways. A) The volcano plot of RNA‐seq illustrates the expressions of up‐regulated genes (orange dots) and downregulated genes (green dots) in the model and PPT NMs groups. B,C) Box plots of IL‐17 signaling B) and ferroptosis C) pathways based on GSVA‐KEGG. D) The heatmap of DEGs in IL‐17 signaling and ferroptosis pathways. E–J) mRNA levels of AP‐1 E), NF‐κB1 F), TRAF6 G), IL‐6 H), CXCL1 I), and CXCL3 J) in the IL‐17 signaling pathway. K–M) mRNA levels of ZIP14 K), DMT1 L), and FTH M) in the ferroptosis pathway. N,O) Confocal microscopic observation N) and quantitative analysis O) of intracellular Fe^2+^ levels. P,Q) Flow cytometric analysis P) and confocal microscopic observation Q) of intracellular LPO. In all cases, hPDLSCs were treated with medium alone (control) or 10 µg mL^−1^ LPS (model), while cells in the PPT NMs group were treated with 10 µg mL^−1^ LPS and PPT NMs (100 µg mL^−1^) for 24 h, followed by different analyses. R) A diagram illustrates the signaling pathways. Data are presented as means ± SD (A–D, *n* = 3; E–M, O, P, *n* = 5). ^*^ *p* < 0.05, ^**^ *p* < 0.01, and ^***^ *p* < 0.001.

According to the above findings on the excellent bioactivities of PPT NMs, further mechanistic studies were performed. At 24 h after hPDLSCs were treated with different formulations, RNA‐sequencing (RNA‐seq) was conducted. Based on the empirical Bayes method, there were a total of 1004 differentially expressed genes (DEGs), including 340 upregulated and 664 downregulated DEGs, when the PPT NMs group was compared with the model group (**Figure** **4**A). Additionally, by comparing the model group with the control group, we identified 4243 DEGs, with 2189 upregulated and 2054 downregulated genes (Figure S8A). Further, in the upset plot, we found 327 high‐expressed DEGs in the model group, which were notably decreased after PPT NMs treatment. Meanwhile, PPT NMs increased 163 low‐expressed DEGs (Figure S8B, Supporting Information). In addition, Gene Ontology (GO) analysis suggested that co‐expressed, up‐ and down‐regulated DEGs mainly enriched in the regulation of neutrophil homeostasis (purple box) and inflammatory responses (red boxes) (Figure S9, Supporting Information). As expected, GO analysis showed that PPT NMs treatment inhibited immune and inflammatory responses (Figure S10, Supporting Information). Moreover, Kyoto Encyclopedia of Genes and Genomes (KEGG) pathway enrichment analyses of all DEGs as well as co‐expressed DEGs between up‐regulated genes of the model group and the down‐regulated genes of the PPT NMs group both revealed significant changes in the ferroptosis and IL‐17 signaling pathways in hPDLSCs subjected to different treatments (Figures S11 and S12, Supporting Information). Gene set variation analysis (GSVA) further affirmed the KEGG results (Figure S13, Supporting Information). In particular, GSVA verified that PPT NMs notably inhibited both IL‐17 signaling and ferroptosis pathways (Figure 4B,C). Given the close relationship between the IL‐17 signaling pathway and inflammation, as well as the association of the ferroptosis pathway with cell apoptosis and ROS production, the following mechanistic studies were concentrated on the IL‐17 signaling pathway and ferroptosis.

We found that AP‐1, NF‐ΚB1, TRAF6, IL‐6, CXCL1, AND CXCL3 genes were up‐regulated in the model group, as compared with the control group, while PPT NMs effectively inhibited their expressions (Figure 4D–J). These results suggested that PPT NMs can normalize the inflammatory environment by inhibiting the IL‐17 signaling pathway. Also, RNA‐seq and PCR quantification revealed notably higher mRNA levels of ZIP14, DMT1, and FTH in the model group, which were significantly reduced by PPT NMs (Figure 4D,K–M). In view of their important roles in ferroptosis, this finding implied that PPT NMs can inhibit ferroptosis. Further fluorescence observation after staining with FerroOrange (a Fe^2+^‐specific probe) and ELISA quantification verified significantly increased Fe^2+^ in hPDLSCs of the model group, while PPT NMs effectively decreased the intracellular Fe^2+^ level (Figure 4N,O). Moreover, the detection of intracellular lipid peroxides (LPO) based on an LPO‐specific probe Liperfluo substantiated obvious lipid peroxidation in response to LPS stimulation, and this aberrant effect was rescued by PPT NMs (Figure 4P,Q). Consequently, PPT NMs can suppress ferroptosis in hPDLSCs.

Collectively, these mechanistic studies demonstrated that PPT NMs can effectively inhibit inflammatory responses in hPDLSCs by simultaneously regulating IL‐17 signaling and ferroptosis pathways. Moreover, in the pro‐inflammatory environment, PPT NMs not only eliminate excessive ROS but also reduce the pro‐inflammatory cytokine production and attenuate the recruitment of immune cells. These effects collectively contribute to the resolution of inflammation, restoration of the inflammation‐impaired osteogenic differentiation capacity of stem cells, and promoted tissue repair (Figure 4R).

### 2.4 Preparation and Characterization of a PPT NMs‐Loaded Bioactive Nanofiber Patch

**Figure 5:**
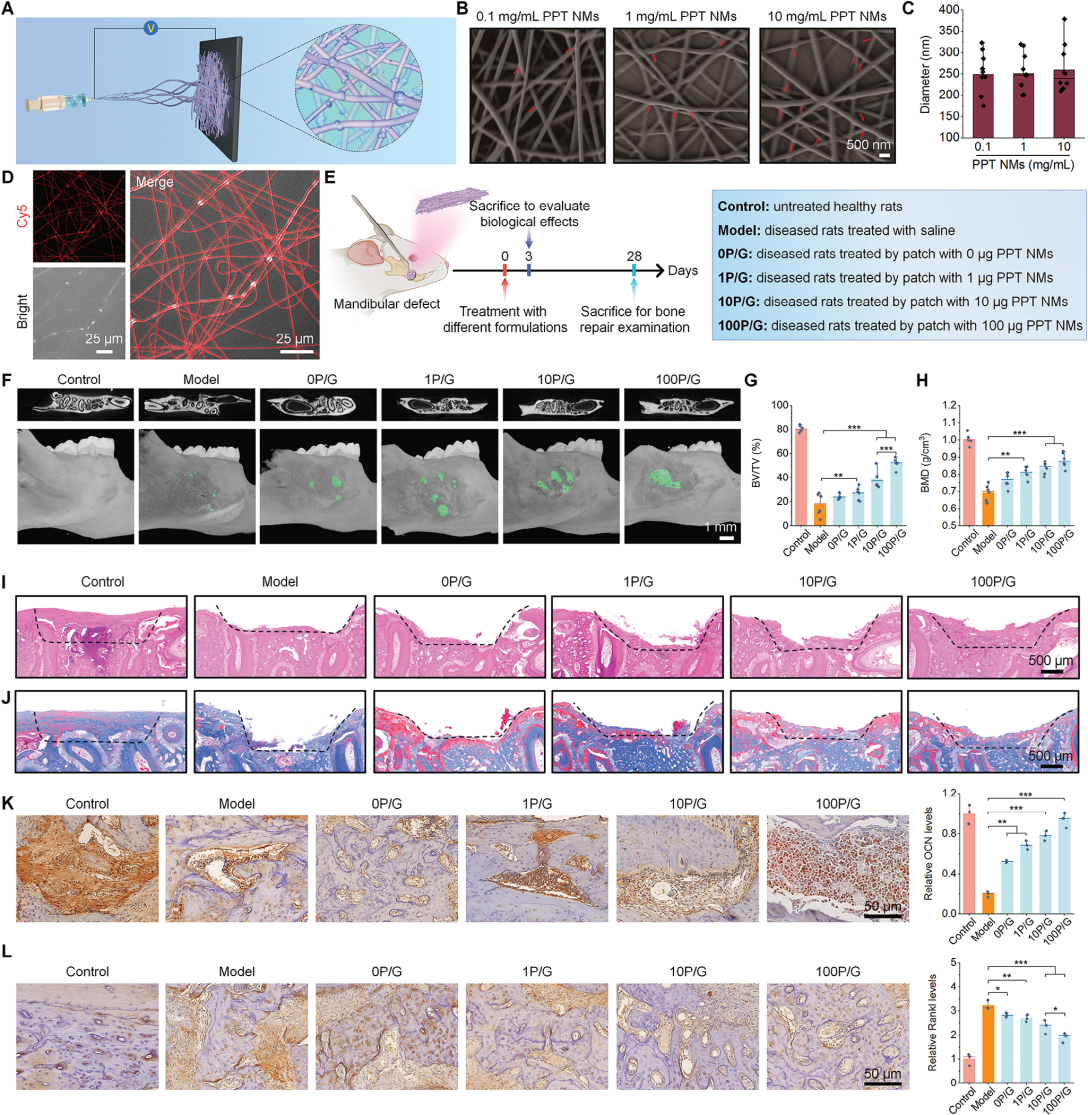
Engineering of a PPT NMs‐loaded bioactive patch to promote osteogenesis in rats with mandibular defects. A) A sketch shows preparation of PPT NMs‐loaded nanofibrous patches based on PVA and GE by electrospinning. B,C) SEM images B) and quantified diameter C) of bioactive nanofibers containing different contents of PPT NMs. D) Confocal microscopy observation of Cy5‐PPT NMs distribution in the bioactive patch P/G. E) Schematic illustration of the establishment of a mandibular bone defect model in rats and treatment procedures. F) 2D transverse section images (upper) and 3D images (lower) by micro‐CT illustrate mandibular defect regeneration in different groups at day 28 after different treatments. Bone regeneration regions in 3D images are indicated by green. G,H) Quantification of BV/TV G) and BMD H) in the mandibular defect areas. I) Histological analysis of defect tissue sections after staining with H&E. J) Masson staining of defect tissue sections. Blue areas indicate collagen deposition, while red areas indicate other components. K,L) IHC analysis of OCN K) and RANKL L) in the bone defect areas at day 28 after different treatments. In both cases, the quantitative results are shown in the right panel. Data are presented as means ± SD (C, *n* = 10; G, H, *n* = 6; K, L, *n* = 3). ^*^ *p* < 0.05, ^**^ *p* < 0.01, and ^***^ *p* < 0.001.

Extensive studies have demonstrated that nanofiber patches can not only serve as scaffolds for different cells to realize tissue engineering,^[^ ^25^ ^]^ but also be used as drug carriers to achieve localized and sustained drug delivery.^[^ ^26^ ^]^ Herein we hypothesize that PPT NMs‐loaded patches can function as bioactive scaffolds for bone regeneration by promoting the implantation/proliferation of pro‐osteogenic cells and attenuating inflammatory reactions. As a proof of concept, two biocompatible polymers polyvinyl alcohol (PVA) and gelatin (GE) were employed to prepare nanofibrous patches with desirable biocompatibility, biodegradability, and mechanical strength by electrospinning, a well‐established technology extensively used for fabricating functional nanofiber structures for drug delivery and tissue regeneration. PVA and GE were blended at different mass ratios to fabricate nanofiber patches. The mass ratio of PVA/GE at 2:8 was screened by morphology observation and diameter quantification via scanning electron microscopy (SEM) (Figure S14, Supporting Information), since previous studies demonstrated that nanofiber patches with smaller diameters show higher mechanical strength.^[^ ^27^ ^]^ The nanofiber patch based on PVA/GE at a weight ratio of 2:8, defined as P/G, was used in the following studies. In vitro tests showed that P/G gradually hydrolyzed in PBS, minimum essential medium α (α‐MEM), and artificial saliva, with the fibrous structure being maintained for ≈1 month (Figure S15, Supporting Information). Then different contents of PPT NMs were loaded into P/G to engineer bioactive patches (**Figure** **5**A). SEM observation revealed slightly increased diameters after loading PPT NMs, showing protrusions or swollen regions on nanofibers (Figure 5B,C). Further confocal microscopy observation of P/G containing Cy5‐PPT NMs indicated a uniform distribution of PPT NMs in the resulting nanofiber patch (Figure 5D). Moreover, in line with the hydrolysis profile of P/G, in vitro release of PPT NMs from the nanofiber patch can be sustained for ≈1 month (Figure S16, Supporting Information). These results demonstrated that PPT NMs can be effectively loaded into the P/G nanofiber patch and released in a sustained manner, thus affording a bioactive patch with sustained activities.

### 2.5 Bioactive Patches Promote the Bone Formation in Rats with Mandibular/Cranial Defects by Regulating the Osteoimmune Environment

Using the patch containing Cy5‐PPT NMs, in vivo fluorescence imaging suggested that the locally implanted patch retained in the mandibular defect for ≈7 days, while it maintained in the cranial defect for ≈2 weeks (Figure S17, Supporting Information). Then we examined in vivo biological effects of the bioactive nanofiber patches containing 0, 1, 10, or 100 µg PPT NMs, which are defined as 0P/G, 1P/G, 10P/G, and 100P/G, respectively (Figure 5E). In rats with mandibular defects, significantly higher levels of IL‐6, TNF‐α, ROS, and myeloperoxidase (MPO) were detected around mandibular defects at day 3 post‐treatment with saline, compared with the control group (Figure S18, Supporting Information), indicating the existence of a pro‐inflammatory and oxidative microenvironment. Treatment with bioactive patches effectively reduced the levels of these pro‐inflammatory cytokines and oxidative mediators, in a PPT NMs dose‐dependent manner, with the best effect being achieved by 100P/G. Consistently, immunohistochemistry (IHC) analysis revealed that treatment with bioactive patches significantly reduced the MPO expression, indicating a substantial decrease in neutrophil infiltration and activation in the bone defect tissues (Figure S19, Supporting Information). Furthermore, the local tissue sections exhibited elevated levels of forkhead box P3 (FOXP3), IL‐10, and transforming growth factor (TGF)‐β, coupled with reduced expressions of RAR‐related orphan receptor gamma t (RORγt), IL‐17, and IL‐6 after treatment. These results suggest that PPT NMs can regulate the Treg/Th17 cell balance in mandibular defects (Figure S20, Supporting Information). In line with our finding based on the animal model, evaluation of global transcriptomic changes by RNA‐seq of human gingival tissues of patients with moderate or severe periodontitis (there were bone defects in these cases) and healthy ones from the GEO database revealed significantly higher infiltration of neutrophils in the diseased group, as compared to the healthy group (Figure S21A,B, Supporting Information). Additionally, the Treg cell population was significantly lower in the diseased group (Figure S21C,D, Supporting Information). Collectively, these results demonstrated that our bioactive patches can effectively improve the osteoimmune environment by attenuating the neutrophil infiltration, suppressing local oxidative distress, and regulating the Treg/Th17 cell balance to maintain immune homeostasis and normal bone metabolism, thereby promoting a favorable locus for bone regeneration.

Subsequently, the bone repair effects of bioactive patches were evaluated. Analysis by micro‐computed tomography (micro‐CT) revealed notably promoted bone regeneration by bioactive patches, and the defect repair capability was also associated with the dose of PPT NMs (Figure 5F). Further quantitative analysis showed that bone volume per tissue volume (BV/TV), bone mineral density (BMD), bone surface per tissue volume (BS/TV), trabecular number (Tb.N), and trabecular thickness (Tb.Th) were dramatically decreased in the model group (Figure 5G,H; Figure S22, Supporting Information). By contrast, treatment with bioactive patches remarkably increased these bone formation‐related indicators. Consistently, examination of hematoxylin and eosin (H&E)‐stained histological sections at day 28 after different treatments indicated that bioactive patches notably narrowed the range of the defects with newly formed tissues (Figure 5I). Analysis of Masson‐stained sections revealed much more collagen generation and mineral deposition in the defects after intervention with bioactive patches (Figure 5J). Moreover, IHC analysis of tissues surrounding the defect showed significantly higher expression of OCN after intervention with bioactive patches, particularly at 100 µg PPT NMs (Figure 5K). In line with increased bone regeneration, bioactive patches notably decreased receptor activator of NF‐κB ligand (RANKL)‐positive osteoclasts, compared to the model group, indicating simultaneous inhibition of bone resorption (Figure 5L). Consequently, these results unambiguously demonstrated that bioactive patches can efficiently promote bone formation in rats with mandibular defects.

**Figure 6:**
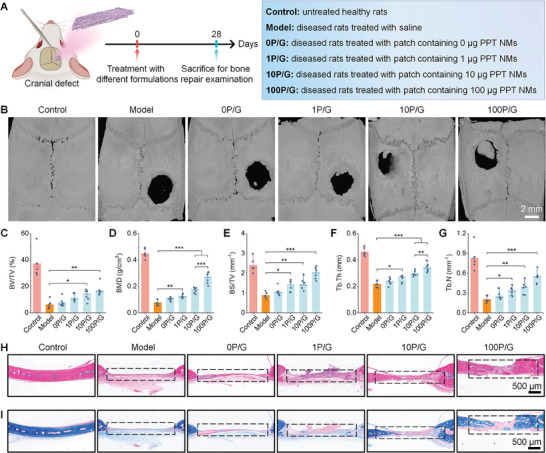
The bioactive patch promotes osteogenesis in rats with cranial defects. A) A sketch shows the establishment of a cranial bone defect model in rats and treatment protocols. B) 3D micro‐CT images illustrate cranial defect regeneration at day 28 after different treatments. C–G) Quantification of BV/TV C), BMD D), BS/TV E), Tb.Th F), and Tb.N G) in cranial defect areas. H,I) Histological analysis of defect tissue sections after staining with H&E H) or Masson I). Blue areas indicate collagen deposition, while red areas indicate other components. Data are presented as means ± SD (*n* = 6). ^*^ *p* < 0.05, ^**^ *p* < 0.01, and ^***^ *p* < 0.001.

Next, we confirmed the bone regeneration performance of the bioactive patch in rats with cranial defects, another well‐recognized bone defect model frequently used to evaluate bone regeneration effects of different therapies and tissue engineering strategies (**Figure** **6**A).^[^ ^28^ ^]^ Micro‐CT and quantitative analyses revealed notably lower bone formation in the defect area of the model group, while treatment with the PPT NMs‐loaded bioactive patch effectively increased bone mass, in a PPT NMs dose‐dependent manner (Figure 6B–G). Inspection of histological sections stained with H&E or Masson further affirmed the newly formed bone and collagen fibrils in the defect area after treatment with bioactive patches, especially in 100P/G group (Figure 6H,I).

Taken together, the above results clearly demonstrated that the newly engineered bioactive patches can efficaciously promote bone regeneration in two rat models of mandibular and cranial defects, largely by relieving inflammation and normalizing the osteoimmune microenvironment. In both cases, the bone repair efficacy was closely related to the nanotherapy dose.

### 2.6 Engineering of a Stem Cell Sheet for Constructing a Multifunctional Janus Patch

**Figure 7:**
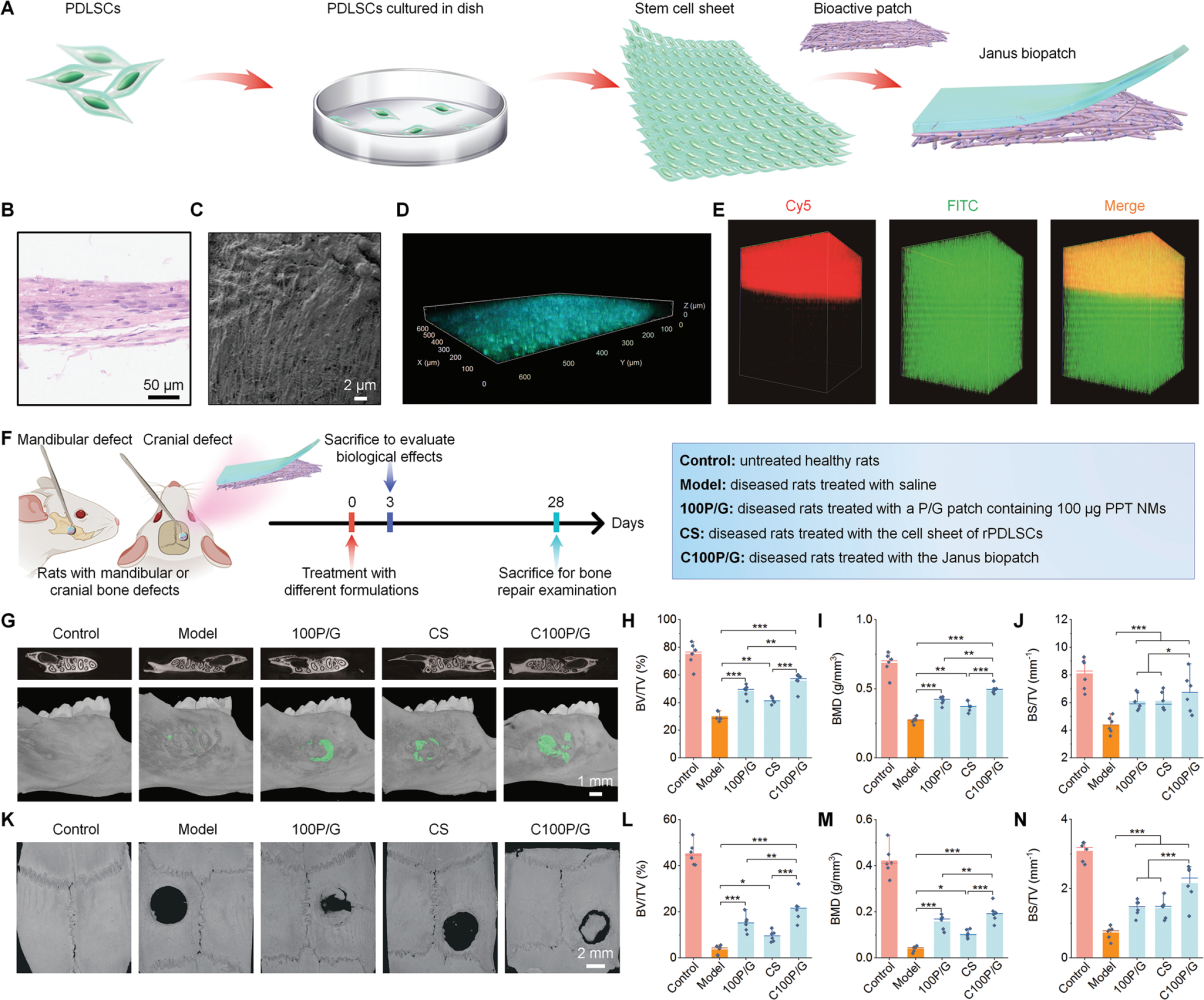
Construction of a multifunctional Janus patch to promote bone regeneration in rats. A) Schematic of engineering a Janus biopatch based on a stem cell sheet and a bioactive nanofibrous membrane. B) A typical optical micrograph shows the H&E‐stained rPDLSCs cell sheet. C) SEM image of the stem cell sheet. D) Confocal microscopic image shows the rPDLSC sheet labeled with FITC and DAPI. E) 3D reconstruction of confocal images of the Janus patch based on a Cy5‐labeled nanofibrous membrane and a FITC‐labeled rPDLSCs sheet. F) A workflow indicates treatment procedures. G) 2D transverse section (upper) and 3D (lower) micro‐CT images illustrate mandibular defect regeneration after different treatments. Bone regeneration regions in 3D images are indicated by green. H–J) Quantification of BV/TV H), BMD I), and BS/TV J) in mandibular defect areas. K) 3D micro‐CT images show cranial defect regeneration in different groups. L–N) Quantification of BV/TV (L), BMD M), and BS/TV N) in cranial defect areas. Data are presented as means ± SD (*n* = 6). ^*^ *p* < 0.05, ^**^ *p* < 0.01, and ^***^ *p* < 0.001.

Besides shaping the pathological environment into a niche more suitable for cells associated with bone regeneration, a sufficient number of stem cells are necessary for successful tissue repair.^[^ ^29^ ^]^ Accordingly, we further proposed a stem cell sheet‐based bilayer strategy to realize more effective and synergistic bone tissue repair (**Figure** **7**A), on the basis of the above engineered bioactive patch. To this end, rat PDLSCs (rPDLSCs) were isolated and identified by flow cytometry (Figure S23, Supporting Information). Then, rPDLSCs were cultured, passaged, and finally obtained rPDLSCs sheets (Figure S24A, Supporting Information). H&E staining indicated that the sheet was composed of multiple layers of cells and the extracellular matrix (ECM), and cells were arranged uniformly, with the deposition of ECM between cells (Figure 7B). SEM observation revealed a substantial amount of ECM secretion on the cell sheet surface, with the layered deposition and encapsulated cells (Figure 7C). Besides, confocal microscopy indicated layered proliferation and high density of rPDLSCs in the obtained cell sheet (Figure 7D). Further, the superior osteogenic capacity of the cell sheet was verified by ALP and ARS staining, after osteogenic induction for 14 days (Figure S24B, Supporting Information). Moreover, we found that the rPDLSCs sheet exhibited normal paracrine effects, as implicated by the notable secretion of growth factors, such as insulin‐like growth factor 1 (IGF‐1), vascular endothelial growth factor (VEGF), and TGF‐β (Figure S25, Supporting Information). Collectively, these results demonstrated the successful engineering of the rPDLSCs sheet with desirable pro‐osteogenic and paracrine effects.

Before constructing the bilayer membrane (defined as the Janus biopatch), the rPDLSCs sheet was labeled with DiR. In vivo, fluorescence imaging indicated that the implanted cell sheet could be maintained in the mandibular/cranial defects for over 28 days (Figure S26, Supporting Information). This implied that rPDLSCs in the cell sheet can survive in the defects and exert their functions for a long period of time. Further, it was found that paracrine actions of the rPDLSCs sheet were considerably impaired by LPS‐mediated inflammation, as implied by the significantly reduced secretion of IGF‐1, VEGF, and TGF‐β, compared to the control cell sheet. By contrast, when the LPS‐induced rPDLSCs sheet was simultaneously treated with 100P/G, its paracrine activity was effectively rescued (Figure S27, Supporting Information). Then, a Janus patch consisting of the rPDLSCs sheet and the bioactive membrane 100P/G was constructed by directly placing the cell sheet onto the bioactive membrane (Figure 7A). After the cell sheet was labeled with FITC and the bioactive membrane was labeled with Cy5, confocal microscopy observation revealed a characteristic bilayer structure for the obtained Janus patch (Figure 7E), which was further confirmed by SEM (Figure S28, Supporting Information). Notably, cells from the sheet could infiltrate into the bioactive film, which should be attributed to the porosity and swelling of 100P/G after water absorption that facilitated the infiltration of rPDLSCs into the film matrix. Consequently, a multifunctional Janus patch (defined as C100P/G) derived by integrating a stem cell sheet and a bioactive membrane was successfully engineered.

### 2.7 The Janus Patch Facilitates Bone Defect Repair by Simultaneously Alleviating Pathological Abnormalities and Inducing Osteogenesis in Rats

For the engineered bilayer patch C100P/G, its anti‐inflammatory effects were evaluated in rats with mandibular defects (Figure 7F). At day 3 after local implantation, the bilayer patch C100P/G significantly reduced the levels of typical pro‐inflammatory and oxidative mediators (IL‐6, TNF‐α, ROS, and MPO) in the tissues around mandibular defects, with effects comparable to those of the bioactive film 100P/G (Figure S29, Supporting Information).

Further, we examined the bone regeneration capability of C100P/G in rats with mandibular defects. Compared with the model group, the 100P/G, CS, and C100P/G groups showed considerable new bone formation, as implicated by micro‐CT imaging and quantitative analysis of BV/TV, BMD, BS/TV, Tb.Th, and Tb.N (Figure 7G–J; Figure S30A,B, Supporting Information). Consistently, histological analyses of H&E or Masson‐stained sections also revealed notably increased new bone and collagen deposition in three treated groups (Figure S30C,D, Supporting Information). It is worth noting that the best bone repair effect was afforded by treatment with C100P/G. Moreover, C100P/G demonstrated similar pro‐osteogenic effects in rats with cranial bone defects (Figure 7K–N; Figure S31, Supporting Information).

Together, these results substantiated that the multifunctional Janus biopatch integrating with an inflammation‐resolving film and a pro‐osteogenic stem cell sheet can remarkably promote bone repair in both mandibular and cranial bone defect models in rats, by synergistically regulating the local osteoimmune environment, attenuating apoptosis/ferroptosis of stem cells, thus facilitating osteogenic differentiation and paracrine effects of stem cells. This bioengineered patch can be applied to treat various inflammation‐related bone defects.

### 2.8 Tannic Acid‐Coating Improves Bioadhesion and Bone Regeneration Capabilities of the Janus Biopatch C100P/G

**Figure 8:**
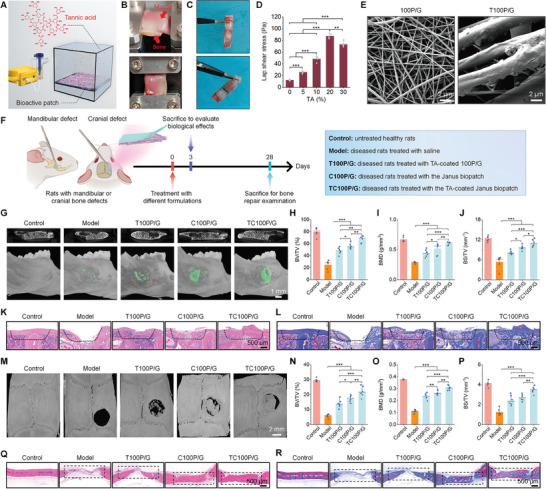
Functionalization of the Janus biopatch with TA to promote osteogenesis in rats with mandibular/cranial defects. A) Schematic of functionalizing the bioactive patch 100P/G with TA via aerosolization. B,C) Digital photos illustrate adhesion force test setups C) and strong adhesion of rat bone‐bone or muscle‐bone tissues C) by TA‐functionalized 100P/G. D) The quantified lap shear stress between the rat muscle and bone tissues that were bound together with the bioactive patches coated with different concentrations of TA. E) SEM observation of morphology of nanofibers with or without TA. F) Schematic illustration of treatment procedures. G) 2D transverse section (upper) and 3D (lower) micro‐CT images illustrate mandibular defect regeneration in different groups. Bone regeneration regions in 3D images are indicated by green. H–J) Quantification of BV/TV H), BMD I), and BS/TV J) in mandibular defect areas. K,L) Histological analysis of mandibular defect tissue sections stained with H&E K) or Masson L). M) 3D micro‐CT images show cranial defect regeneration after different treatments. N–P) Quantification of BV/TV N), BMD O), and BS/TV P) in cranial defect areas. Q,R) Histological analysis of cranial defect tissue sections stained with H&E Q) or Masson R). Data are presented as means ± SD (D, *n* = 4; H–J, N–P, *n* = 6). ^*^ *p* < 0.05, ^**^ *p* < 0.01, and ^***^ *p* < 0.001.

From the viewpoint of clinical applications, the retention of functional patches at bone defect sites needs to be prolonged to afford better tissue repair effects. We hypothesize that the bone regeneration capacity of C100P/G can be further improved by increasing its tissue adhesion capacity, prolonging retention time, and sustaining the functions of PPT NMs and stem cells. As a biocompatible polyphenolic compound, TA has been widely used for constructing different adhesive materials for a diverse array of biomedical applications.^[^ ^30^ ^]^ Therefore, we further functionalized 100P/G by coating TA onto the Janus biopatch via aerosolization to improve its adhesive ability (**Figure** **8**A). First, we explored the adhesion capacity of PPT NMs‐containing bioactive patches after aerosolization coating with different concentrations (0, 5, 10, 20, and 30%) of TA on both sides, using rat muscles and cranial bones (Figure 8B). It was found that the bioactive P/G film coated with TA could effectively adhere to bone and muscle tissues (Figure 8C). Adhesive force tests confirmed that lap shear stress gradually increased with increasing in TA (Figure 8D), with the highest adhesion for the patch obtained at 20% TA. Therefore, coating with 20% TA was performed for the following studies. After TA coating, the diameter of nanofibers was increased from 0.2 ± 0.1 to 2.7 ± 0.6 µm, as indicated by SEM images (Figure 8E; Figure S32A, Supporting Information). The successful coating of TA was confirmed by the Ag deposition test, UV–visible and FTIR spectrometry, and X‐ray photoelectron spectroscopy (Figure S32B–F, Supporting Information). Also, these results indicated the presence of hydrogen bonding between TA and P/G, other than a simple physical mixture. Accordingly, TA can be effectively decorated onto 100P/G by simple nebulization. Importantly, the tissue adhesive ability of the Janus biopatch was significantly increased after TA coating.

In line with enhanced tissue adhesion after TA functionalization, in vivo imaging indicated that TA‐coated bioactive patches displayed considerably prolonged retention in both mandibular (≈14 days) and cranial (over 28 days) defects (Figure S33, Supporting Information), compared to TA‐deficient patches. Based on the TA‐coated 100P/G film (defined as T100P/G) and rPDLSCs sheet, a bioadhesive Janus patch (TC100P/G) was further developed. In rats with mandibular defects, local treatment with different TA‐containing patches demonstrated desirable anti‐inflammatory effects, as implicated by significantly decreased TNF‐α, IL‐6, ROS, and MPO (Figure S34, Supporting Information), indicating that TA coating did not affect the inflammation‐resolving activity of C100P/G. Then, the pro‐osteogenic effects of TC100P/G were evaluated in rats with mandibular and cranial defects, using C100P/G as a control (Figure 8F).

In both animal models, TC100P/G showed more effective bone repair outcomes, compared to T100P/G and C100P/G, as implicated by the quantitative data of BV/TV, BMD, BS/TV, Tb.Th, and Tb.N, as well as histological analyses of tissue sections stained with H&E or Masson (Figure 8G–R; Figures S35 and S36, Supporting Information). These results substantiated that the bone regeneration capability of the multifunctional Janus biopatch based on the stem cell sheet and bioactive membrane can be effectively enhanced by improving the tissue adhesive capacity with bioadhesive molecules.

### 2.9 Safety Studies

Finally, in vivo, the safety profiles of T100P/G, C100P/G, and TC100P/G were examined. During and after treatment, there were no significant changes in the body weight and organ index of major organs, including the heart, liver, spleen, lung, and kidney, for all examined groups (Figure S37, Supporting Information). In addition, the levels of representative hematological parameters and biomarkers relevant to liver/kidney functions were in normal ranges for all treatment groups, as compared to the normal control group (Figure S38, Supporting Information). Furthermore, examination of H&E‐stained sections of major organs revealed no obvious pathological changes or injuries for all rats subjected to different treatments (Figure S39, Supporting Information). These preliminary results suggested that local application of the multifunctional patch TC100P/G did not cause adverse effects. This is in line with the fact that all the used materials in the finally engineered patch possess excellent biocompatibility. Importantly, the patch can be hydrolyzed into water‐soluble and biocompatible molecules that can be easily excreted.

## 3 Discussion

Bone defects often coincide with inflammation, which can severely harm bone tissue and impair its structure and function. This is particularly evident in conditions like inflammatory bone resorption,^[^ ^31^ ^]^ osteoarthritis,^[^ ^32^ ^]^ and periodontitis,^[^ ^33^ ^]^ causing considerable distress for patients. Although certain growth factors can facilitate bone regeneration, their use is hindered by challenges such as low bioavailability, short half‐life, and the risk of ectopic bone formation.^[^ ^34^ ^]^ Moreover, a single growth factor frequently falls short in achieving optimal regeneration effects, while it is very difficult to enhance efficacies by identifying the best combination of different growth factors. Stem cell therapies have been demonstrated promising for bone regeneration. In addition to serving as a source of osteoblasts, stem cells can release various beneficial growth factors like VEGF, IGF‐1, and TGF‐β to further boost bone regeneration.^[^ ^2^, ^35^ ^]^ However, severe inflammation and other pathological conditions can cause abnormal proliferation and differentiation of local stem cells, thus resulting in poor bone regeneration outcomes.^[^ ^22^, ^36^ ^]^ Increasing evidence has demonstrated that the combination of stem cells and anti‐inflammatory or osteoimmunomodulatory biomaterials represents an intriguing strategy toward the effective regeneration of different tissues.^[^ ^5^, ^37^ ^]^ Herein we propose a multifunctional Jannus biopatch strategy for bone regeneration, in which a nanotherapy‐containing bioactive layer enables effective regulation of the osteoimmune microenvironment, while the cell sheet affords adequate stem cells for bone regeneration. Further functionalization of the biopatch significantly improves its adhesion to the defect and prolongs local retention and release of nanotherapy, thereby providing superior bone formation effects.

First, we engineered a totally biodegradable, amphiphilic, and bioactive conjugate PPT that can self‐assemble into a nanotherapy PPT NMs. In both acute and chronic inflammation models based on hPDLSCs, PPT NMs notably reduced the expression of IL‐6 and IL‐17 in vitro. IL‐6 plays a crucial role in mediating inflammation and bone resorption, while IL‐17 can activate the NF‐κB signaling pathway, leading to the secretion of pro‐inflammatory and chemotactic factors.^[^ ^38^ ^]^ Consequently, PPT NMs can effectively suppress inflammatory responses in hPDLSCs, a type of stem cell capable of promoting osteogenesis through delicately controlled differentiation. By inhibiting inflammatory responses, PPT NMs effectively enhanced the osteogenic differentiation capacity of hPDLSCs under chronic inflammatory conditions. This is in line with the previous finding that suppression of inflammation can create a favorable microenvironment for the osteogenesis of stem cells.^[^ ^21^, ^23^ ^]^ Mechanistically, PPT NMs alleviate inflammatory responses in hPDLSCs by regulating IL‐17 signaling and ferroptosis pathways. Available studies have demonstrated that inhibition of IL‐17 signaling can downregulate inflammatory cytokine/chemokine expressions, reduce neutrophil infiltration, and promote differentiation of T helper cells into Treg cells.^[^ ^39^ ^]^ Additionally, attenuation of ferroptosis enables reduced release of free iron and ROS as well as decreased cell death and inflammation, which positively regulates bone regeneration.^[^ ^40^ ^]^ Consequently, PPT NMs can be used as an effective nanotherapy to enhance stem cell functions during bone regeneration.

At inflammatory bone defect sites, stem cells or progenitor cells from local or distant tissues will be recruited to facilitate bone regeneration.^[^ ^41^ ^]^ These cells receive signals from a variety of growth factors and cytokines involved in tissue repair, actively participating in localized bone regeneration processes.^[^ ^42^ ^]^ By loading PPT NMs into PVA/GE nanofibers, a bioactive patch was fabricated by electrospinning. Thus engineered bioactive patch 100P/G effectively inhibited local inflammatory and oxidative stress responses as well as normalized the osteoimmune environment, thereby promoting bone repair in rats with mandibular or cranial bone defects, in a PPT NMs dose‐dependent manner. Furthermore, the bioactive patch promoted Treg cell differentiation, as implicated by increased expressions of Foxp3 (a Treg cell marker), TGF‐β (a Treg cell‐inducing cytokine), and IL‐10 (an anti‐inflammatory cytokine produced by Treg cells) in bone defect tissues, which is beneficial for bone regeneration.^[^ ^43^ ^]^ Consistently, we found that treatment with 100P/G reduced Th17 cell populations, characterized by decreased levels of IL‐17, RORγt, and IL‐6. Notably, IL‐17 generally serves as a Th17 cell marker, while RORγt and IL‐6 play pivotal roles in driving Th17 cell differentiation and proliferation.^[^ ^44^ ^]^ By inhibiting osteogenic differentiation and activating osteoclasts, Th17 cells may enhance bone resorption.^[^ ^11^, ^45^ ^]^ Accordingly, by enhancing Foxp3/IL‐10/TGF‐β and reducing IL‐17/RORγt/IL‐6 expressions, the bioactive patch effectively modulated the Treg/Th17 cell balance, thus regulating osteoimmunity to promote bone repair and regeneration at the defect site. Moreover, IHC analysis showed that intervention with the bioactive patch increased OCN but decreased RANKL expressions. Since OCN is a marker relevant to late‐stage osteogenic differentiation and maturation, while RANKL is closely associated with osteoclastogenesis,^[^ ^46^ ^]^ these results suggest that 100P/G can promote bone regeneration and simultaneously inhibit bone resorption. Collectively, our PPT NMs‐loaded bioactive patch can efficaciously reshape the osteoimmune‐inflammatory microenvironment in damaged tissues by simultaneously regulating IL‐17 signaling and ferroptosis, therefore it is a promising platform to potentiate stem cell therapies.

Subsequently, we constructed a Janus biopatch by integrating the bioactive patch with a pro‐osteogenic stem cell sheet, to circumvent limitations associated with direct injection of stem cells and stem cell sheets in bone tissue regeneration. As demonstrated by previous studies, stem cells implanted into the bone defect can differentiate into osteoblasts and secrete growth factors for bone repair.^[^ ^41^ ^]^ We found that the nanotherapy PPT NMs can rescue the paracrine effects of the rPDLSCs sheets under a pathological condition, by resolving inflammation. Besides, the implanted rPDLSCs sheet could survive in the bone defects in rats for a long time period, in line with the previous study on stem cell retention in vivo.^[^ ^47^ ^]^ In the treatment of mandibular/cranial bone defects, the anti‐inflammatory membrane attached to the defect gradually degraded and slowly released PPT NMs, thereby effectively alleviating local inflammatory responses and regulating the osteoimmune environment. Concurrently, the degradation of the fibrous membrane facilitated the survival of stem cells, which then differentiated into osteoblasts, leading to superior regenerative and repair effects on bone defects. Compared to the stem cell sheet and anti‐inflammatory fibrous membrane alone, the Janus biopatch showed more significantly enhanced bone repair effects in both mandibular and cranial bone defect models in rats by synergistically regulating the local osteoimmune environment as well as facilitating survival and osteogenic differentiation of stem cells.

Lastly, considering the possible detachment or displacement of the implanted biomaterials at the defect sites due to bone tissue movement alongside physical activities, the Janus patch was further functionalized to enhance its adhesion capacity and prolong tissue retention. To address this issue and achieve long‐acting effects, we further functionalized the engineered Janus patch with TA, a polyphenolic compound that can enhance wet bio adhesion by increasing interactions with different biomolecules in tissues.^[^ ^30^ ^]^ Previous studies demonstrated that TA can crosslink with GE through hydrogen bonding.^[^ ^48^ ^]^ Also, it has been reported that TA exhibits high binding affinity to biomolecules in different tissues.^[^ ^30^, ^49^ ^]^ Our results indicated that TA‐coating remarkably enhanced the tissue adhesive capacity of the bioactive membrane, which should be attributed to increased noncovalent interactions between the patch and tissue via multiple hydrogen‐bonding‐mediated cross‐linking effects. Therefore, the improved bioadhesive capacity enabled prolonged retention of the TA‐coated Janus biopatch and sustained biological functions of both the nanotherapy and stem cells at the bone defect site, thereby enhancing regenerative capability. Moreover, the superior bone regeneration outcomes of C100P/G and TC100P/G over T100P/G indicated that combining the stem cell sheet provides an enriched source of stem cells with osteogenic differentiation and paracrine effects, which facilitate bone regeneration and repair. Compared to previously reported biomaterial systems for bone regeneration, our Janus biopatch has multiple advantages, such as ease to produce, versatility for the addition of functions, excellent bone tissue regeneration capability, good in vivo safety, and operational convenience. Consequently, it holds great potential for clinical translation.

## 4 Conclusion

In summary, we successfully engineered Janus biopatches integrating different functional moieties for bone regeneration. Our multifunctional biopatch can be used for treating various bone diseases associated with inflammation. In view of the excellent drug loading and controlled release capability of electrospun nanofibers and the availability of various types of stem cells, this bioactive nanofiber‐based bilayer strategy can also be applied to promote the translation of stem cell therapies in other diseases, such as cardiovascular diseases, gastrointestinal diseases, spinal cord injury, wound healing, and skin burns.

## Conflict of Interest

The authors declare no conflict of interest.

## Supporting information

Supporting Information

## Data Availability

The data that support the findings of this study are available from the corresponding author upon reasonable request.
