# Supplementary material for: An Osteoimmunomodulatory Biopatch Potentiates Stem Cell Therapies for Bone Regeneration by Simultaneously Regulating IL‐17/Ferroptosis Signaling Pathways
Source: Adv Sci (Weinh). 2024 Jul 18;11(35):2401882. doi: 10.1002/advs.202401882 (PMC11425236; doi:10.1002/advs.202401882)
Supplement: Supplementary file 1 — Supporting Information [file ADVS-11-2401882-s001.docx]

Supporting Information

**An Osteoimmunomodulatory Biopatch Potentiates Stem Cell Therapies for Bone Regeneration by Simultaneously Regulating IL-17/Ferroptosis Signaling Pathways**

*Shan Liu, Wenle Wang, Zhiyu Chen, Peng Wu, Wendan Pu, Gang Li,^*^ Jinlin Song,^*^ Jianxiang Zhang^*^*

S. Liu, W. L. Wang, J. L. Song

Chongqing Key Laboratory of Oral Diseases and Biomedical Sciences, Chongqing Municipal Key Laboratory of Oral Biomedical Engineering of Higher Education, Stomatological Hospital of Chongqing Medical University, Chongqing Medical University, Chongqing 401147, P. R. China

E-mail: songjinlin@hospital.cqmu.edu.cn

S. Liu, W. L. Wang, Z. Y. Chen, P. Wu, W. D. Pu, G. Li, J. X. Zhang

Department of Pharmaceutics, College of Pharmacy, Third Military Medical University (Army Medical University), Chongqing 400038, P. R. China

E-mail: jxzhang1980@gmail.com; jxzhang@tmmu.edu.cn

W. L. Wang

Department of Orthodontics II, Affiliated Stomatological Hospital of Zunyi Medical University, Zunyi 563000, P. R. China

Z. Y. Chen

Department of Orthopedics, The First Affiliated Hospital of Chongqing Medical University, Chongqing 400016, P. R. China

P. Wu

College of Pharmacy and Medical Technology, Vocational and Technical College, Hanzhong, Shaanxi 723000, P. R. China

G. Li

Department of Stomatology, Southwest Hospital, Third Military Medical University (Army Medical University), Chongqing 400038, P. R. China

E-mail: ligang@tmmu.edu.cn

J. X. Zhang

State Key Laboratory of Trauma and Chemical Poisoning, Third Military Medical University (Army Medical University), Chongqing 400038, P. R. China

J. X. Zhang

Yu-Yue Pathology Scientific Research Center, 313 Gaoteng Avenue, Jiulongpo District, Chongqing 400039, P. R. China

**Materials and methods**

**Materials**

Hexachlorocyclotriphosphazene (HCCP), N-(tert-butoxycarbonyl) glycine (Boc-Gly), 4-(dimethylamino) pyridine (DMAP), 4-(hydroxymethyl) phenylboronic acid pinacol ester (PBE), N, N′-dicyclohexylcarbodiimide (DCC), 4-hydroxy-2,2,6,6-tetramethylpiperidine-1-oxyl (TP), sodium hydride (NaH), N-Boc-ethylenediamine (N-Boc-EDA), pyrene, 2,2-diphenyl-2-picrylhydrazyl (DPPH), lipopolysaccharide (LPS), 2',7'-dichlorofluorescin diacetate (DCFH-DA), 4',6-diamidino-2-phenylindole (DAPI), and tannic acid (TA) were purchased from Sigma-Aldrich (USA). Trifluoroacetic acid (TFA), triethylamine (TEA), anhydrous dichloromethane (DCM), anhydrous 1, 4-dioxane (DO), and anhydrous tetrahydrofuran (THF) were purchased from J&K Scientific Ltd (Beijing, China). mPEG-amine (PEG-NH_2_, Mw = 2000) was obtained from Laysan Bio, Inc (USA). Cyanine5 NHS ester (Cy5-NHS) was obtained from Lumiprobe, LLC (USA). Penicillin, streptomycin, fetal bovine serum (FBS), and α-MEM medium were provided by Gibco (Waltham, USA). Amplex Red Hydrogen Peroxide/Peroxidase Assay Kit was obtained from Thermo Fisher Scientific (USA). BCA protein assay kit was obtained from Beyotime Biotechnology (Shanghai, China). The CCK-8 cell proliferation kit was purchased from MedChemExpress (USA). Human mesenchymal stem cell surface marker detection kit (Cat# HUXMX-09011) and rat mesenchymal stem cell surface marker detection kit (Cat# RAXMX-09011) were purchased from Cyagen (China). APC Annexin V apoptosis kit was purchased from Biolegend (USA). DiR (Cat# BMD0074) was purchased from Abbkine (USA). ELISA kit of rat insulin-like growth factor 1 (rIGF-1) was purchased from Biorbyt (UK), while other ELISA kits were all obtained from Boster Biological Technology (China). Polyvinyl alcohol (PVA, > 99% hydrolyzed, Mw = 115,000) and gelatin type A (GE) from porcine skin were obtained from Sigma-Aldrich (USA). Artificial saliva was purchased from Solarbio (China). All other reagents are commercially available and used as received.

**Synthesis of a bioactive amphiphile PPT**

A bioactive amphiphile (defined as PPT) was synthesized by sequential nucleophilic substitution of TP, PEG-NH_2_, and PBE onto HCCP. First, TP-conjugated HCCP (HCCP-TP) was synthesized. Specifically, TP (0.35 g, 2 mmol) was dissolved in anhydrous THF (10 mL) followed by the gradual addition of NaH (0.16 g, 4 mmol) under nitrogen protection. The reaction mixture was allowed to react at room temperature for 3 hours. The resulting mixture was then added dropwise to a solution containing HCCP (0.71 g, 2 mmol) in anhydrous THF (10 mL) at -20°C and stirred for additional 3 hours. The desired product HCCP-TP was obtained by purification via chromatography on silica gel (hexane: ethyl acetate = 5:1), giving rise to a yellow solid. To synthesize glycine-conjugated PBE (Gly-PBE), Boc-Gly (4.50 g, 25.5 mmol), DMAP (0.32 g, 2.55 mmol), and PBE (6.00 g, 25 mmol) were co-dissolved in 200 mL of DCM. The reaction was performed at 0ºC for 1 h, followed by the addition of DCC (5.30 g, 25.5 mmol). After 3 h of reaction at room temperature, the reaction mixture was filtered, washed, and dried with Na_2_SO_4_, followed by rotary evaporation to obtain Boc-Gly-PBE. Subsequently, the obtained product was de-protected in a solvent mixture of CF_3_COOH/CH_2_Cl_2_ (1:3, 40 mL) for hours. The obtained precipitate was washed with cold ether, filtrated, and then dried under vacuum, yielding a white solid. Subsequently, HCCP-TP (0.29 g, 0.6 mmol) and TEA (168 μL, 1.2 mmol) were dissolved in 10 mL of dry DO at -20ºC, into which PEG-NH_2_ (1.0 g, 0.5 mmol) dissolved in 10 mL of dry DO was added. After overnight reaction at room temperature, Gly-PBE (1.05 g, 3.6 mmol) and 1 mL of TEA in 10 mL dry DO were added dropwise into this reaction system, which was stirred under nitrogen protection at 75°C for another 48 hours. To collect the final product, the reaction solution was filtered, concentrated under vacuum, and the residue was dissolved in 5 mL of DCM and precipitated in 45 mL of cold diethyl ether for 3 times. The final product obtained was a yellowish solid.

To synthesize a Cy5-labeled conjugate, i.e., PPCy5, N-Boc-EDA (327 mg, 2 mmol) was dissolved in 10 mL of dry THF and added dropwise to a solution containing 710 mg of HCCP (2 mmol) and 615 μL of TEA in 10 mL of dry THF. After being stirred magnetically at -20°C for 3 h, the mixture was filtered, concentrated in vacuum, and purified by chromatography on silica gel (hexane: ethyl acetate = 5:1) to obtain a white solid, i.e., N-Boc-EDA-conjugated HCCP (N-Boc-EDA-HCCP). Then, PEG-NH_2_ was conjugated with N-Boc-EDA-HCCP, according to the aforementioned procedures. For thus obtained product, after being deprotected in CF_3_COOH /CH_2_Cl_2_ (1:3) solution and purified by precipitation from Et_2_O, it was reacted with Cy5-NHS (0.1 mmol) in the presence of TEA (0.1 mmol) in DCM. After stirring in the dark at room temperature for 24 h, the final product was purified by precipitation from Et_2_O.

**Materials characterization**

For different materials, their ^1^H NMR spectra were obtained using an Agilent DD2rometer operating at 600 MHz, while Fourier transform infrared (FTIR) spectra were recorded using a PerkineElmer FT-IR spectrometer (100S, USA).

**Preparation and characterization of PPT-derived nanomicelles (NMs)**

PPT NMs were synthesized by directly dissolving PPT in deionized water at appropriate concentrations, followed by sonication for 10 min. The resulting solution was allowed to statically equilibrate at room temperature for 30 min. Similarly, Cy5-labeled fluorescent PPT NMs (defined as Cy5-PPT NMs) were prepared by mixing PPT and PPCy5 at a mass ratio of 10:1.

The size, size distribution profile, and zeta-potential value of PPT NMs were determined using using a Malvern Zetasizer (Nano ZS90, Malvern Instruments) at 25°C, in which 1 mL of aqueous solution containing 1 mg/mL PPT NMs was used. The morphology of NMs was observed via transmission electron microscopy (TEM) using a TECNAI-10 microscope (Philips, Netherlands). To prepare the sample for TEM observation, the formvar-coated Cu grid was dipped into an aqueous solution containing 1 mg/mL PPT NMs for 5 min. The grid was then stained with 1% phosphotungstic acid for 1 min for negative staining. After excessive solution was removed, the TEM samples were allowed to dry at room temperature on filter paper before TEM observation.

**Hydrolysis of PPT in the presence of hydrogen peroxide**

To examine hydrolysis of PPT in the presence of H_2_O_2_, PPT was incubated with an aqueous solution containing 10 mM H_2_O_2_ at 37°C for 24 h. The hydrolyzed products were collected by lyophilization and characterized by ^1^H NMR spectroscopy and electrospray ionization mass spectrometry (ESI-MS, Bruker, German). Besides, after PPT was incubated with various concentrations of H_2_O_2_ (0.1, 1, and 10 mM) at 37°C for 24 h, the release profiles of TP and p-(hydroxymethyl) phenol (HMP) in the hydrolyzed samples were analyzed with a Shimadzu high-performance liquid chromatography (HPLC) system (LC-2030C and RF-20A), using a methanol-water eluent (20:80, v:v) at a flow rate of 1.0 mL/min and detected at 254 nm.

**ROS-scavenging abilities of PPT**

To assess the eliminating capability of PPT to superoxide anion (•O_2_^-^), various concentrations of PPT (ranging from 0.063 to 8.0 mg/mL) were incubated with an excess amount of •O_2_^-^. The concentration of remaining •O_2_^-^ was measured using the Superoxide Anion Radical Detection Kit (Nanjing Jiancheng Bioengineering Institute, China) by quantifying the absorbance at 550 nm using an Infinite M200 Pro plate reader (TECAN, USA). Then, the •O_2_^-^-eliminating capacity of PPT was calculated.

To evaluate the free radical-eliminating capability of PPT, different concentrations (ranging from 0 to 4.0 mg/mL) of PPT in methanol (100 μL) were mixed with a fresh methanol solution containing DPPH• (100 μL, 100 μg/mL) in a 96-well plate and incubated in the dark at room temperature. The absorbance of the mixture was then measured at 520 nm at various time intervals, and the amount of eliminated DPPH• was calculated.

To evaluate the capability of PPT to scavenge hydroxyl radical (•OH), different concentrations of PPT (ranging from 0.031 to 4.0 mg/mL) were incubated with an excess amount of •OH. The concentration of remaining •OH was measured using the Hydroxyl Radicals Detection Kit (Nanjing Jiancheng Bioengineering Institute, China). Subsequently, the •OH-eliminating capacity of PPT was calculated by measuring the absorbance at 550 nm.

To determine H_2_O_2_-scavenging capability of PPT, a fresh solution of H_2_O_2_ (10 mM, 500 μL) was mixed with 500 μL of methanol containing different concentrations of PPT (ranging from 0 to 8.0 mg/mL). The mixture was incubated at 37°C for 2 h. The remaining content of H_2_O_2_ was quantified using the Hydrogen Peroxide Assay Kit (Beyotime Biotechnology, China) by measuring the absorbance at 560 nm. The H_2_O_2_-eliminating capacity of PPT was then calculated.

Also, the total ROS-scavenging capability of PPT was quantified. In brief, various concentrations of PPT (ranging from 0.5 to 8.0 mg/mL) were incubated with an excess amount of ABTS•+/ABTS. Trolox was used as a reference, and the total antioxidant capacity was calculated by measuring the absorbance at 734 nm.

**Animals**

Sprague-Dawley rats (aged 2 or 8-10 weeks) were obtained from the Animal Center of the Third Military Medical University. Rats were maintained under standard laboratory conditions, with free access to food and water. All animals were acclimatized to the laboratory for a minimum of 7 days before experiments. All animal experiments were approved by the Animal Ethics Committee at Third Military Medical University, Chongqing, China (approval number: AMUWEC20201180).

**Isolation and cell culture of human/rat periodontal ligament stem cells**

Human periodontal ligament stem cells (hPDLSCs) were isolated from extracted premolars of adolescents undergoing orthodontic treatment. All experiments regarding hPDLSCs were approved by the Human Ethics Committee of Chongqing Medical University (Chongqing, China), approval No. CQHS-REC-2022 (LSNo.167). Rat periodontal ligament stem cells (rPDLSCs) were isolated from extracted incisors of rats (aged 2 weeks). hPDLSCs and rPDLSCs were cultured in α-MEM supplemented with 10% FBS and 1% penicillin-streptomycin at 37°C in a humidified 5% CO_2_ incubator. The culture media were replaced every 3 days. hPDLSCs and rPDLSCs from passages 3-5 were used for subsequent experiments. To induce osteogenesis, the normal medium was supplemented with 50 μg/mL ascorbic acid (Solarbio, China), 10 mM β-glycerophosphate (Sigma-Aldrich, USA), and 100 nM dexamethasone (Solarbio, China).

**Identification of hPDLSCs and rPDLSCs by flow cytometry**

The stem cell characteristics of hPDLSCs were verified using flow cytometry to detect cell surface molecular markers, including CD44, CD166, CD29, CD73, CD45, and HLA-DR. For the third generation of cells, a total of 3 × 10^6^ cells were fixed in 4% formaldehyde for 30 min and incubated with primary antibodies including purified anti-human antibodies of CD44, CD166, CD29, CD73, CD45, and HLA-DR (1:50) for 30 min at 4°C. Subsequently, the secondary FITC-labeled goat anti-mouse (1:50) CD166, CD29, CD73, CD45, and HLA-DR antibodies, as well as FITC-labeled goat anti-rat (1:50) CD44 antibody, were added and incubated at 4°C for 30 min. Finally, the samples were analyzed using a FACS Calibur flow cytometer (BD Biosciences).

Similarly, rPDLSCs were identified by flow cytometric analysis of related cell surface molecular markers, including CD34, CD29, CD11b/c, and CD14. Briefly, cells were fixed and incubated with the corresponding primary mouse anti-rat antibodies (1:50) for 30 min. Then the secondary FITC-labeled goat anti-mouse (1:50) were added and incubated at 4°C for 30 min, followed by flow cytometric measurement.

**In vitro cellular uptake** **of PPT NMs in hPDLSCs**

hPDLSCs were seeded onto sterilized glass coverslips in 12-well plates at a density of 1 × 10^5^ per well. The cells were incubated with 1 mL of normal medium for 24 h and then switched to 1 mL of fresh medium containing 50 μg/mL Cy5-PPT NMs. Confocal laser scanning microscopy (CLSM, Zeiss) observation was performed, after cells were fixed with 4% paraformaldehyde and stained with DAPI at different time points (0, 1, 2, 4, and 8 h) post incubation. To quantify internalized Cy5-PPT NMs, hPDLSCs were seeded at a density of 1 × 10^5^ cells per well in a 12-well plate with 1 mL of fresh medium for 24 h, followed by incubation with 1 mL of medium containing 50 μg/mL Cy5-PPT NMs. After incubation for different time periods (0, 1, 2, 4, and 8 h), cells were digested and fluorescent intensities were assessed by fluorescence activated cell sorting via flow cytometry. Similar methods were applied to examine the dose-dependent internalization profile, after cells were incubated with different concentrations of Cy5-PPT NMs (0, 1, 5, 10, 50, and 100 μg/mL) for 4 h via CLSM and flow cytometry.

**In vitro cytotoxicity evaluation in hPDLSCs**

hPDLSCs were seeded in 96-well plates at a density of 3 × 10^4^ cells per well and cultured with PPT NMs at different concentrations (10, 50, and 100 µg/mL). The cell viability was assessed using CCK-8 assay at days 1, 3, and 7 after incubation.

**Cell apoptosis assay**

hPDLSCs were seeded in 6-well plates and incubated overnight. The cells were then treated with 10 μg/mL LPS and different concentrations of PPT NMs (0, 10, 50, or 100 μg/mL). After incubation for 24 h, the cells were rinsed three times using PBS, collected, and stained with APC Annexin V apoptosis kit. Apoptotic cells were determined using a FACS Calibur flow cytometer.

**Measurement of intracellular ROS generation in hPDLSCs**

To investigate the inhibitory effect of PPT NMs on intracellular ROS generation in LPS-stimulated hPDLSCs, the cells were seeded in a 6-well plate (3 × 10^5^ cells per well) and incubated in normal medium for 24 h. Then the cells were stimulated with LPS (10 μg/mL) and treated with various concentrations (0, 10, 50, or 100 μg/mL) of PPT NMs for 24 h. The cells were washed with PBS and incubated with a ROS-sensitive fluorescent dye DCFH-DA (10 μM) for 30 min. The fluorescence intensity was then measured by flow cytometry.

Additionally, to visualize ROS-induced DCFH-DA fluorescence, hPDLSCs were cultured in glass-bottom cell culture dishes at a density of 1 × 10^5^ cells/plate overnight. Subsequently, cells were treated through similar procedures as mentioned above. Fluorescent images were then captured by CLSM.

**In vitro anti-inflammatory effects of PPT NMs in hPDLSCs**

To examine inflammation-resolving effects of PPT NMs in LPS-stimulated hPDLSCs, the cells were seeded in a 6-well plate (3 × 10^5^ cells per well) and incubated in normal medium for 24 h. Then, cells were treated with LPS (10 μg/mL) or LPS plus different doses of PPT NMs (ranging from 0, 10, 50, to 100 µg/mL) for 24 h. After different treatments, the supernatant was collected and the levels of interleukin (IL)-6 and IL-17 were quantified by ELISA. Besides, the level of H_2_O_2_ was acquired by Amplex Red Hydrogen Peroxide/Peroxidase Assay Kit (Thermo Fisher Scientific, USA). In addition, intracellular mRNA levels of IL-6, IL-17, and heme oxygenase-1 (HO-1) were quantified by real-time quantitative PCR (qPCR) after treatment with different formulations.

**In vitro pro-osteogenic effects of PPT NMs in hPDLSCs under inflammatory conditions**

To explore the pro-osteogenic effect of PPT NMs in hPDLSCs under inflammatory conditions, cells were treated with LPS (10 μg/mL) or LPS in combination with different doses of PPT NMs (ranging from 0, 10, 50, to 100 µg/mL) in pro-osteogenic medium. After 28 days of incubation, the supernatant was collected for analyzing the secretion of inflammatory cytokines (IL-6 and IL-17) and H_2_O_2_. In addition, the cells were collected to determine the mRNA levels of IL-6, IL-17, HO-1, Runx2, ALP, OPN, and OCN at pre-defined time points.

**Real-time quantitative PCR**

After cells were treated with various formulations, total RNA was isolated using the Trizol reagent (Takara, Japan). The concentrations of RNA were determined using a NanoDrop Lite spectrophotometer (Thermo Scientific, USA). Subsequently, 1 µg total RNA was reverse-transcribed into cDNA using the PrimeScript RT Master Mix (Takara, Japan), following the manufacturer’s instructions. Real-time qPCR was conducted on a LightCycler96 (Roche, Swiss) utilizing the SYBRII qPCR master mix (Takara, Japan). The gene primers utilized are provided in Table S1. The relative expression levels of different genes were evaluated using the ΔΔCt method and normalized to the housekeeping gene GAPDH.

**Western blotting analysis**

Total protein extraction was performed using RIPA buffer (Bioss Antibodies) supplemented with 1% PMSF (Bioss Antibodies). Then the concentrations were determined using the BCA Protein Assay Kit (Beyotime). After that, the proteins (30 μg/lane) were separated by 10% (wt/vol) SDS-PAGE and transferred to PVDF membranes (Millipore, USA). After blocking with 5% fat-free dry milk in TBST (TBS with 0.1% Tween-20) for 2 h at room temperature, the membranes were incubated overnight at 4°C with primary antibodies against GAPDH (1:1000, Bioworld Technology), ALP (1:800, ET1601-21, Huabio), Runx2 (1:1000, ab236639, Abcam), HO-1 (1:1000, ab52947, Abcam), IL-6 (1:1000, A21264, abclonal), or IL-17 (1:1000, 66148-1-lg, Proteintech). Subsequently, the membranes were washed with TBST and incubated with the corresponding horseradish peroxidase-conjugated secondary antibodies (1:50000, Proteintech, China) at room temperature for 1.5 h. After another round of washing with PBST, the protein bands were detected using the Immobilon Western Chemiluminescent HRP Substrate (Millipore, USA). Protein band analysis was performed using ImageJ software.

**In vitro mineralization evaluations of hPDLSCs after different treatments**

On day 7 after inducing osteogenesis, hPDLSCs were washed three times with PBS and fixed using 4% paraformaldehyde for 30 min. To assess alkaline phosphatase (ALP) activity, the BCIP/NBT ALP staining kit (Beyotime, China) was applied following the manufacturer's instructions. Moreover, on day 21 after osteogenic induction, hPDLSCs were fixed with 4% paraformaldehyde and stained with 0.2% Alizarin Red S (ARS, Solarbio, China) to visualize calcium deposition. Then cells were washed three times with deionized water and observed under a phase-contrast microscope (Leica, German). Quantitative analysis of the images was performed using ImageJ software.

**Transcriptome sequencing of hPDLSCs after treatment with PPT NMs**

hPDLSCs were seeded in 6-well plates, followed by treatment with or without 100 μg/mL PPT NMs or LPS (10 μg/mL) for 24 h. Total RNA was isolated from hPDLSCs using the conventional Trizol extraction method. The Illumina NovaSeq 6000 system was employed by Sinotech Genomics Co., Ltd (Shanghai, China) to sequence the cDNA samples. To obtain high-quality sequencing data, the reads were filtered using SOAPnuke (v1.2) to remove reads containing the sequencing adapter, low-quality base ratio (base quality less than or equal to Q-score) over 20%, and unknown bases (N base ≥ 5%). The remaining clean reads were stored in FASTQ format and mapped onto the reference genome using HISAT2 (v2.0.4). Then, Bowtie2 (v2.2.5) was applied to map clean reads to the reference coding gene set, and the expression level of the genes was determined using StringTie (v2.1.2). DESeq2 (v1.4.5) was utilized to identify the differentially expressed genes (DEGs) with a rigorous threshold (Q value ≤ 0.05). Then, the volcano plots were drawn by R package “ggplot2” (version 3.3.6). The upset plots were drawn by R package “UpSetR” (version 1. 4. 0). To gain insights into changes in the phenotype, GO and KEGG enrichment analyses of the annotated DEGs were performed in “clusterProfiler” R package (version 4.4.4). The significant levels of terms and pathways were Bonferroni-corrected with a rigorous threshold (Q value ≤ 0.05). Meanwhile, Gene Set Enrichment Analysis (GSVA) was carried out based on these two datasets and were ordered top 10 of activated signaling pathway according to adjust *P*-value in “clusterProfiler” R package (version 4.4.4). Also, GSVA box plots were obtained based on KEGG in “clusterProfiler” R package (version 4.4.4). The heatmap was drawn by pheatmap (v1.0.12) according to the DEGs in different samples.

**Quantification of intracellular Fe^2+^ and lipid peroxide in hPDLSCs subjected to different treatments**

To measure the levels of intracellular Fe^2+^, a FerroOrange probe (Dojindo Shanghai, China) was utilized. After treatment with or without 100 μg/mL PPT NMs or LPS (10 μg/mL) for 24 h, hPDLSCs were rinsed with HBSS solution and incubated with a working solution of FerroOrange (1 μmol/L) at 37°C for 30 min. Then, the stained cells were observed by CLSM (Ex: 543 nm; Em: 580 nm), and quantitative analysis was conducted by a fluorescence microreader (Ex: 543 nm; Em: 580 nm) (Thermo Scientific, USA).

Additionally, to measure the levels of intracellular lipid peroxide (LPO), a Liperfluo probe (Dojindo Shanghai, China) was applied. After incubaiton with or without 100 μg/mL PPT NMs or LPS (10 μg/mL) for 24 h, hPDLSCs were rinsed with serum-free α-MEM and incubated with a working solution of Liperfluo (2 μmol/L) at 37°C for 30 min. Then the supernatant was discarded, and cells were rinsed twice with serum-free α-MEM. Finally, the stained cells were observed by CLSM (Ex: 488 nm; Em: 500-550 nm), and the fluorescence intensity was measured by flow cytometry (Ex: 488 nm; Em: 515-545 nm).

**Analyses of microarray data of human gingival tissues**

Microarray datasets GSE10334 (64 healthy samples and 183 diseased samples, https://www.ncbi.nlm.nih.gov/geo/query/acc.cgi?acc=GSE10334) and GSE16134 (69 healthy samples and 241 diseased samples, https://www.ncbi.nlm.nih.gov/geo/query/acc.cgi?acc=GSE16134) were obtained from Gene Expression Omnibus (GEO). CIBERSORT algorithm in R package was used to explore the discrepancy in immune cells between healthy and diseased samples in GSE10334 and GSE16134. Differences in immune cell proportions were evaluated using the Wilcoxon rank-sum test. P < 0.05 was considered statistically significant.

**Preparation, characterization, and degradation properties of bioactive electrospun nanofiber membranes**

Aqueous solutions of PVA (12 wt%) and GE (30 wt%) were thoroughly mixed to obtain PVA/GE solutions with weight ratios varying from 10/0, 8/2, 5/5, 2/8, to 0/10. Then, the mixed solutions were placed into a 5 mL syringe fitted to a needle with a tip diameter of 25 gauges (inner diameter = 0.25 mm). Electrospun fibers were prepared at 15 kV with a feeding rate of 0.5 mL/h using an electrospinning machine (Beijing Ucalery Technology & Development Co., China). A piece of aluminum foil plate was used as a collector. The distance between the needle tip and the collector was 15 cm. Then, the obtained electrospun nanofiber membranes were dried under vacuum. The same procedures were followed to prepare nanofibrous membranes containing PPT NMs or Cy5-PPT NMs, using a 20G needle and at the feeding rate of 0.1 mL/h. The morphology of fibers was observed by scanning electron microcopy (SEM, Crossbeam 340, Zeiss) and their diameter were analyzed by ImageJ. Also, the presence of PPT NMs into PVA/GE nanofiber membranes was observed by CLSM.

To determine the hydrolysis profiles of nanofiber membranes, they were cut into circles with diameter of 6 mm and weighed. Each piece of circle patches was placed into 96-well plates. Then, 300 μL of PBS was added into each well and incubated at 37°C. At different time points (0 h, 1 h, 3 h, 6 h, 12 h, 1 d, 2 d, 3 d, 4 d, 5 d, 6 d, 7 d, 9 d, 11 d, 14 d, 21 d, and 28 d), PBS was collected, and the patches were dried and weighed. The concentration of GE in PBS was determined using the BCA Protein Assay Kit (Beyotime, China), which was used to calculate the rate of nanofiber membrane hydrolysis. Following similar procedures, hydrolysis profiles of the PVA/GE nanofiber membranes in α-MEM solution and artificial saliva (Solarbio, China) were examined.

**In vitro release of PPT NMs from nanofiber patches**

To assess the release of PPT NMs from nanofiber patches, Cy5-PPT NMs-loaded nanofiber patches were cut into circles with diameter of 6 mm. Each piece of the patches was placed into 96-well plates. Then, 300 μL of PBS was added into each well and incubated at 37°C. At different time points (days 0, 1, 3, 7, 14, 21, and 28), the release medium was collected and the concentration of Cy5-PPT NMs was determined by a fluorescence microplate reader (Thermo, USA).

**Preparation and characterization of tannic acid (TA)-coated nanofiber patches**

To enhance the adhesive capacity and prolong the retention time of bioactive PVA/GE nanofiber patches, they were coated with different concentrations of TA (varying from 5%, 10%, 20%, to 30%) by nebulization via an air-compressing nebulizer. The coated patches were dried under vacuum. The surface morphology of TA-coated nanofiber patches was observed by SEM. Additionally, the presence of TA on patches was directly observed by incubation with 0.1 M AgNO_3_ for 1 h. Moreover, FTIR and ultraviolet-visible (US/VIS, Puxi, China) spectra of different samples were acquired to confirm the presence of TA. X-ray photoelectron spectroscopy (XPS) was also conducted using an Axis-Ultra DLD spectrometer (Kratos, UK).

To test adhesion properties of TA-coated patches, the patch-mediated bone-muscle adhesion was evaluated using a Test Bench material testing machine (TA Instruments, USA), following the modified standard test method for strength properties of tissue adhesives (ASTM F2255-05). Lean muscle tissues and cranial bones of rats were used as substrates for the experiments. The patches were placed onto the surface of the muscle section (binding area: 5 × 10 mm^2^), which was immediately covered with the bone tissue. The collected tissues were loaded until complete separation was achieved. The maximum load divided by the binding area was calculated to obtain the apparent lap shear stress (in Pa).

**Preparation and characterization of cell sheets derived from rPDLSCs**

To prepare cell sheets, rPDLSCs were cultured at a density of 1 × 10^5^ cells/well in regular culture medium in 24-well plates until reaching 80-90% confluence. Cells were induced to form cell sheets by supplementing the medium with 50 μg/mL of vitamin C. The culture medium was replaced every 2-3 days for continuous cultivation for 2 weeks. Then the culture plates were placed on ice and carefully scraped from the periphery to the center along the bottom surface to obtain intact cell sheets.

Then, cell sheets were fixed with 4% paraformaldehyde for 24 h. Surface morphology of cell sheets were observed by SEM after gradient dehydration, drying, and gold sputtering. H&E staining was used to observe the cross-sectional structure of the cell sheet. Additionally, after being fixed with polyoxymethylene, cell cytoskeletons were stained with phalloidin, and cell nuclei were stained with DAPI. The three-dimensional structure of the cell sheet was observed by CLSM.

Further, to determine the pro-osteogenic differentiation capacity of cell sheets, they were cultured with osteogenic medium for 14 days. ALP and ARS staining was applied to evaluate the ALP activity and visualize calcium deposition. Next, cell sheets were treated with LPS (10 μg/mL) or LPS combined with 100P/G in the pro-osteogenic medium. After 14 days, both the supernatant and cells were collected. ELISA and RT-qPCR analyses were performed to detect the levels of growth factors, including vascular endothelial growth factor (VEGF), insulin-like growth factor 1 (IGF-1), and transforming growth factor-β (TGF-β) at days 1, 3, 7, and 14.

**Preparation and characterization of multifunctional composite patches**

To prepare multifunctional double layer patches, the cell sheet was placed directly onto the bioactive membrane with or without TA coating. Thus obtained composite patch was used for further experiments. SEM was applied to observe the morphology of this bilayer patch. To observe the interface of the composite patches, the cell sheet was labeled with phalloidin, which was integrated with the Cy5-PPT NMs-containing PVA/GE film, followed by fluorescence imaging via CLSM.

**Establishment of mandibular/cranial defect models in rats and** **treatment procedures**

To establish mandibular defects in rats, a 2-cm incision was made on the skin overlying the mandible after anesthesia. The masseter muscles were carefully separated between the superior and inferior branches of the facial nerve and a defect of 3-mm diameter and 1-mm thickness was created around the periapical area of the molars on the right mandibular bone of rats using a dental trephine. The surgical site was continuously flushed with 0.9% saline to maintain cooling. Immediately after the surgery, different formulations were filled. Finally, the wound was sealed with sutures and treated with povidone iodine.

On the other hand, a dental trephine was used to establish full-thickness cranial defects with diameter of 4 mm on the right side of the cranium, with continuously flushing the area with 0.9% saline for cooling. The surgical site was filled with different formulations and then sutured and sanitized with povidone iodine.

In both cases, six rats were randomly assigned in each group. The model group was treated with saline, while rats with established defects were treated with different formulations. All animals were euthanized at day 28, and mandibular or cranial bones were collected for micro-computed tomography (micro-CT) and histological evaluations.

**In vivo retention of PPT NMs-loaded nanofiber patches or cell sheets in mandibular/cranial defects in rats**

To assess in vivo retention of PPT NMs-containing nanofiber patches in mandibular or cranial defects in rats, Cy5-PPT NMs-loaded patches were placed in the bone defects. Animals were subjected to in vivo imaging at predetermined time points using a Newton 7.0 FT100 imaging system (Vilber Lourmat, France). The fluorescence intensity was calculated by the Living Imaging software (Kuant, France). Similar methods were applied to assess in vivo retention of cell sheets. In this case, cells sheets were labeled with DiR and then transplanted into mandibular or cranial defects in rats. In vivo images were captured at predetermined time points using the Newton 7.0 FT100 imaging system.

**Quantification of inflammatory and oxidative mediators in tissues around the mandibular defect**

After being treated with different formulations, rats were euthanized on day 3 and the mandibular bones surrounding the defects were collected in cold PBS containing 10% FBS. After gently removing the muscles and teeth, bone tissues were carefully ground and subjected to lysis in cytokine lysis buffer for 30 min. After 10 min of centrifugation at 12,000 g and 4°C, the supernatant was collected. The levels of TNF-α, IL-6, and MPO in the supernatant were assessed by ELISA kit, while the H_2_O_2_ concentration was quantified using Amplex Red Hydrogen Peroxide/Peroxidase Assay Kit, according to the manufacturer’s protocols.

**Micro-CT analysis**

To observe and quantitatively analyze the bone formation, mandibular and cranial bones were fixed in 4% polyformaldehyde for 24 h. Then samples were scanned by a Skyscan Micro-CT system (μCT50, Bruker) with a voxel resolution of 10 μm. After three-dimensional reconstruction, the bone volume per tissue volume (BV/TV), bone mineral density (BMD), bone surface per tissue volume (BS/TV), trabecular thickness (Tb.Th), and trabecular number (Tb.N) at the region of interest were assessed by CTAn software (Version 1.20.8.0). The region of interest was defined as the area around the periapical region of defects with diameter of 3 mm and thickness of 1 mm for the mandibular bone, as well as the defect region with diameter of 4 mm and thickness of 1 mm for cranium.

**Histological evaluations**

After micro-CT scanning, the right mandibular bone and cranium were decalcified using EDTA, processed and dehydrated in ethanol gradient, followed by embedding in paraffin and sectioning into 5-μm slices. The sections were then stained with Masson or hematoxylin-eosin (H&E). For immunohistochemical analysis, the tissue sections were rehydrated and then subjected to antigen retrieval by exposing them at 95°C for 30 min. For immunohistochemistry analysis, tissue sections were rehydrated and subjected to antigen retrieval for 30 min at 95°C. The slides were subsequently incubated with rabbit polyclonal anti-Rankl (1:100, Zenbio Inc., China), rabbit polyclonal anti-OCN (1:500, Biorbyt, Cambridge, UK), rabbit monoclonal anti-MPO (1:100, Zenbio, China), rabbit monoclonal anti-TGF-β (1:200, ABclonal, China), rabbit monoclonal anti-IL-6 (1:500, ABclonal, China), rabbit polyclonal anti-IL-17 (1:500, Bioss, China), rabbit polyclonal anti-IL-10 (1:500, Bioss, China), rabbit polyclonal anti-ROR gamma T (RORγt, 1:200, Bioss, China), or rabbit polyclonal anti-Foxp3 (1:500, Bioss, China) overnight at 4°C. At the second day, slides were washed with PBS three times and then incubated with a horseradish peroxidase-conjugated goat anti-rabbit secondary antibody (1:1000, Abcam, UK) for 30 min at room temperature. Immunoreactions were detected with DAB (Proteintech, China). Cell nuclei were stained with hematoxylin (Yike Tianya Biotechnology Co., Chongqing, China). The corresponding positive areas and the integrated optical densities in each slide were quantified using OlyVIA software (Olympus, Japan).

**In vivo safety evaluations**

Mandibular bone defects and cranial defects in rats, established as aforementioned, were treated with different patches. At day 28 after different treatments, blood samples were collected for complete blood count analysis. Serum levels of aspartate aminotransferase (AST), alanine aminotransferase (ALT), blood urea (UREA), and creatinine (CREA) were also quantified. Major organs including the heart, liver, spleen, lung, and kidney were harvested for histological analyses.

**Statistical analysis**

All data are presented as mean ± standard deviation (SD). Statistical analysis was performed by SPSS 26.0 using the one-way ANOVA test for experiments with three or more groups. Statistical significance was considered at *P* < 0.05.

**Table S1.** Primers for quantitative real-time qPCR.

| Genes | Forward 5′—3′ | Reverse 5′—3′ |
| --- | --- | --- |
| (h) IL-6  (h) IL-17  (h) HO-1  (h) ALP | ACTCACCTCTTCAGAACGAATTG  AGATTACTACAACCGATCCACCT  CTCCCAGGGCCATGAACTTT  TAAGGACATCGCCTACCAGCTC | CCATCTTTGGAAGGTTCAGGTTG  GGGGACAGAGTTCATGTGGTA  GGGAAGATGCCATAGGCTCC  TCTTCCAGGTGTCAACGAGGT |
| (h) Runx2  (h) OCN  (h) OPN  (h) NF-κB1  (h) TRAF6  (h) ZIP14  (h) DMT1  (h) FTH  (h) AP-1  (h) CXCL1  (h) CXCL3  (r) VEGF  (r) TGF-β  (r) IGF-1 | GCGGTGCAAACTTTCTCCAG  GGCGCTACCTGTATCAATGG  CAGTTGTCCCCACAGTAGACAC  AACAGAGAGGATTTCGTTTCCG  TTTGCTCTTATGGATTGTCCCC  AAGGCCCTACTCAACCACCT  ATCGGCTCAGACATGCAAGAA  CGAGGTGGCCGAATCTTCC  GGGGCAAGGTGGAACAGTTAT  CGCCCAAACCGAAGTCATAG  CGCCCAAACCGAAGTCATAG  CACGACAGAAGGGGAGCAGAAAG  GACCGCAACAACGCAATCTA  TCACTGCCCAATTGAAATACGA | TCACTGTGCTGAAGAGGCTG  GTGGTCAGCCAACTCGTCA  GTGATGTCCTCGTCTGTAGCATC  TTTGACCTGAGGGTAAGACTTCT  CATTGATGCAGCACAGTTGTC  CGACTGCTCGCTGAAATTGTG  TTCCGCAAGCCATATTTGTCC  GTTTGTGCAGTTCCAGTAGTGA  CCGCTTGGAGTGTATCAGTCA  GGATTTGTCACTGTTCAGCATCTT  GCTCCCCTTGTTCAGTATCTTTT  GGCACACAGGACGGCTTGAAG  GACAGCAATGGGGGTTCTGG  TTAGGCCCAGACAGTTTAAACAAAG |
| (h) GAPDH  (r) GAPDH | GGAGCGAGATCCCTCCAAAAT  GATTGTTGCCATCAACGACC | GGCTGTTGTCATACTTCTCATGG  GTGCAGGATGCATTGCTGAC |

(h) indicates the primers for human cells, while (r) denotes the primers for rat cells.

**
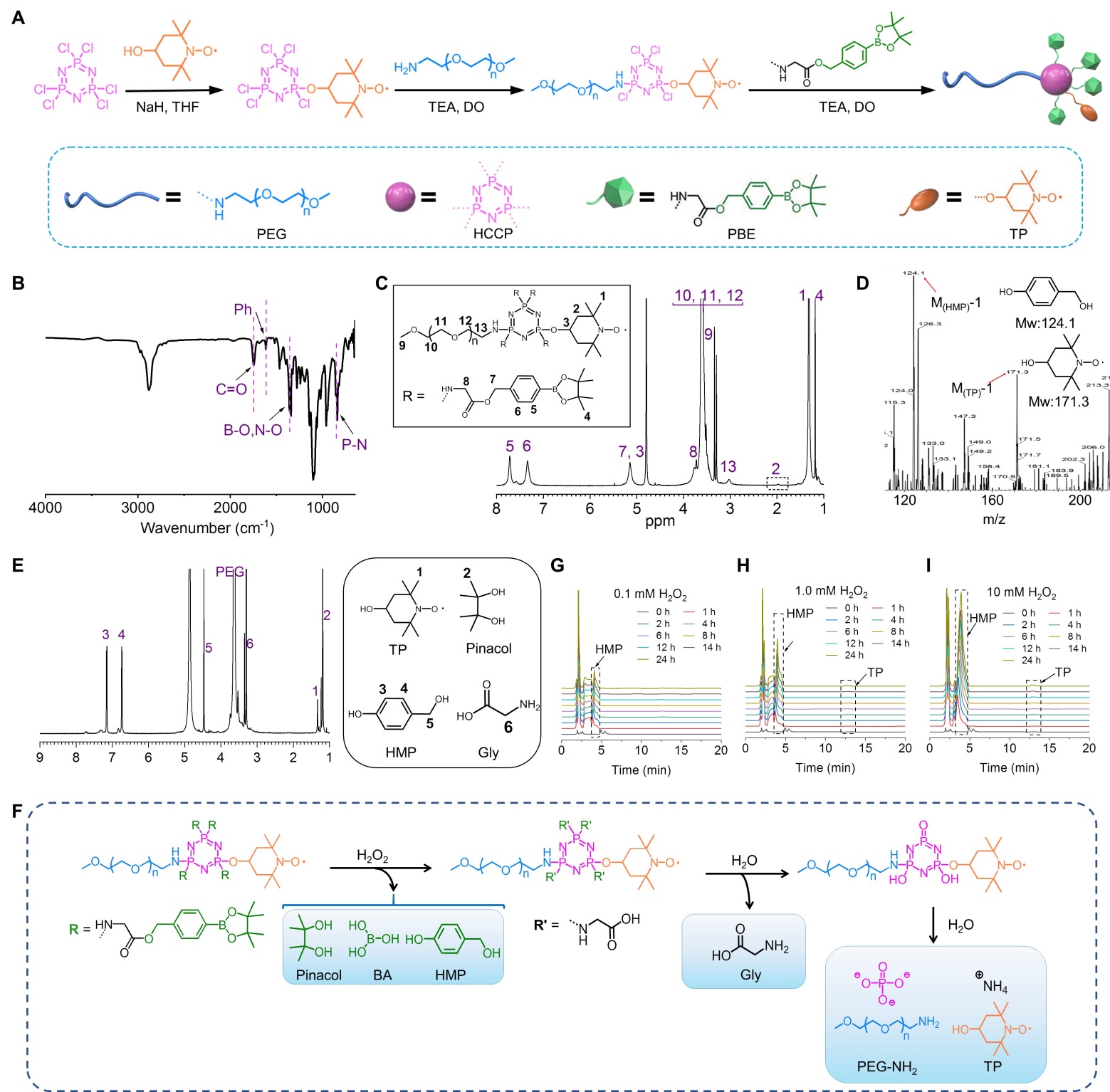
**

**Figure S1.** Synthesis, characterization, and hydrolysis profile of PPT. (A) The synthetic route of PPT. HCCP was conjugated with TP, PEG-NH_2_, and Gly-PBE units by consecutive nucleophilic substitution reactions. THF, tetrahydrofuran; TEA, triethylamine; DO, dioxane. (B-C) FTIR (B) and ^1^H NMR (C) spectra of PPT. (D) Electrospray ionization mass spectrum of the hydrolyzed products of PPT. (E) ^1^H NMR spectrum of PPT in CD_3_OD after incubation with H_2_O_2_. (F) Hydrolysis mechanisms of PPT in the presence of H_2_O_2_. (G-I) HPLC curves show release of HMP and TP from PPT after incubation with various concentrations of H_2_O_2_ (i.e., 0.1, 1, and 10 mM) for different time periods. BA, boric acid; Gly, glycine.


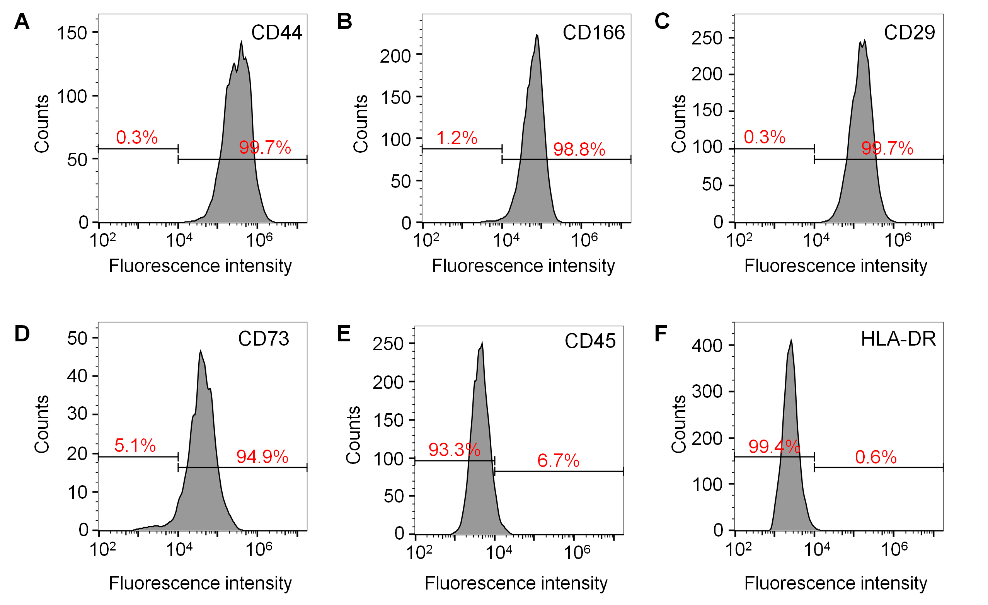


**Figure S2.** Flow cytometry identification of hPDLSCs. (A-F) Flow cytometric quantification illustrates the high expression of surface markers of CD44 (A), CD166 (B), CD29 (C), and CD73 (D), as well as the low expression of CD45 (E) and HLA-DR (F) on hPDLSCs.


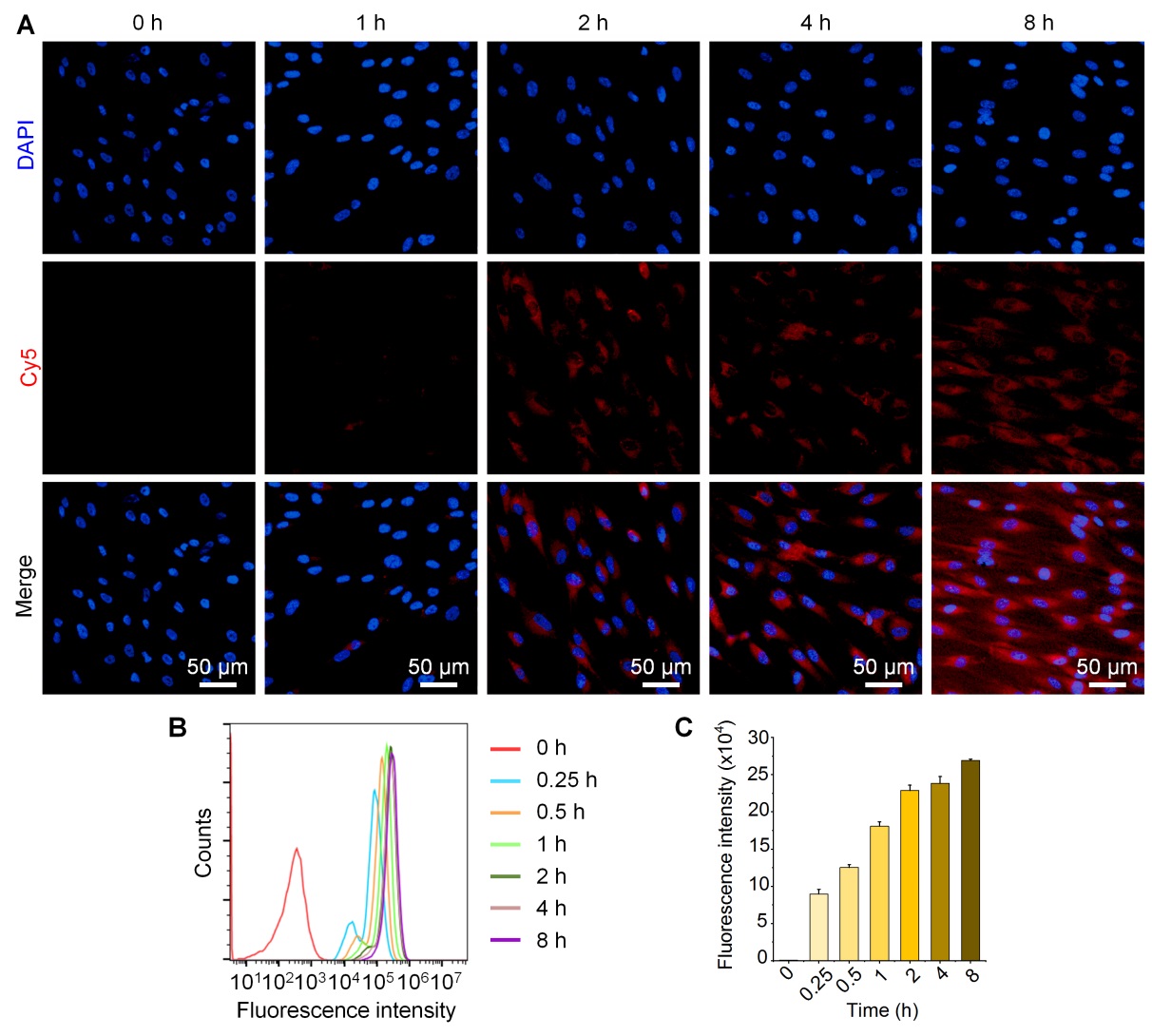


**Figure S3.** Time-dependent cellular uptake of Cy5-PPT NMs in hPDLSCs. (A) Fluorescence images showing time-dependent internalization of Cy5-PPT NMs at 50 µg/mL in hPDLSCs. (B-C) Typical flow cytometric curves (B) and quantitative analysis (C) of time-dependent cellular uptake of Cy5-PPT NMs at 50 µg/mL in hPDLSCs. Data in (C) are presented as mean ± SD (n = 3).


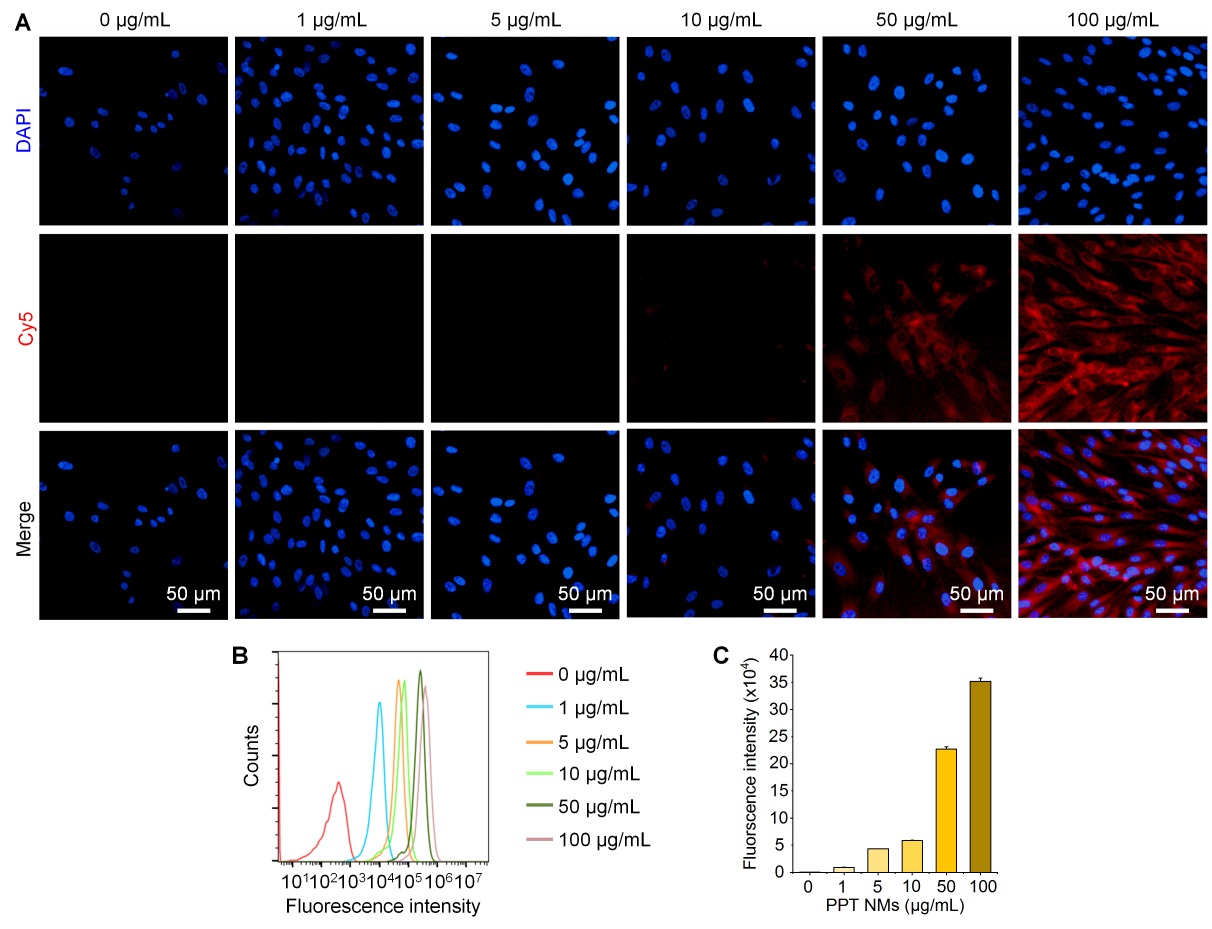


**Figure S4.** Dose-dependent cellular uptake of Cy5-PPT NMs in hPDLSCs. (A) Fluorescence images showing dose-dependent internalization of Cy5-PPT NMs in hPDLSCs after 4 h of incubation. (B-C) Typical flow cytometric curves (B) and quantitative analysis (C) of dose-dependent cellular uptake of Cy5-PPT NMs in hPDLSCs. Data in (C) are presented as mean ± SD (n = 3).


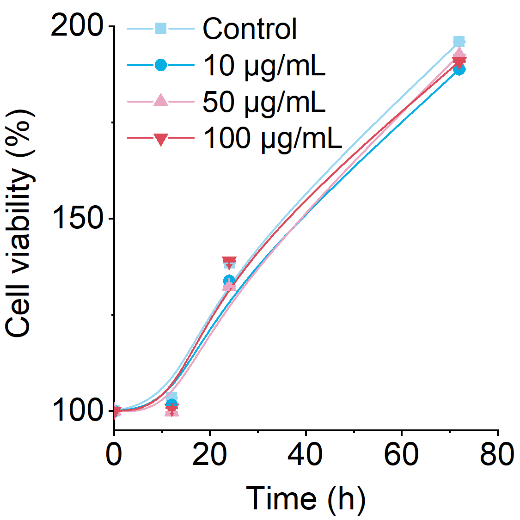


**Figure S5.** Cell viability of hPDLSCs after treatment with different doses of PPT NMs at pre-defined time points. The control group was treated with fresh medium. Data are presented as means ± SD (n = 3).


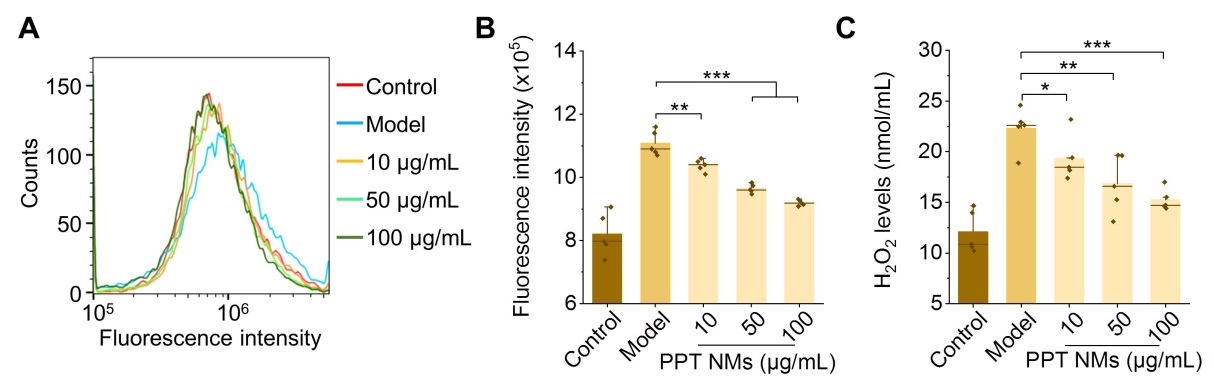


**Figure S6.** The effect of PPT NMs on ROS generation in hPDLSCs. (A-B) Flow cytometric profiles (A) and quantitative analysis (B) of ROS generation in hPDLSCs after different treatments and stained with a fluorescent probe DCFH-DA. (C) Quantified ROS levels in hPDLSCs via Amplex Red Hydrogen Peroxide/Peroxidase Assay Kit. hPDLSCs in the control group were treated with fresh medium, while the model group was induced with 10 µg/mL LPS for 24 h. For PPT NMs groups, cells were treated with 10 µg/mL LPS and various doses of PPT NMs for 24 h. Data in (B-C) are presented as means ± SD (n = 5). *P < 0.05, **P < 0.01, and ***P < 0.001.


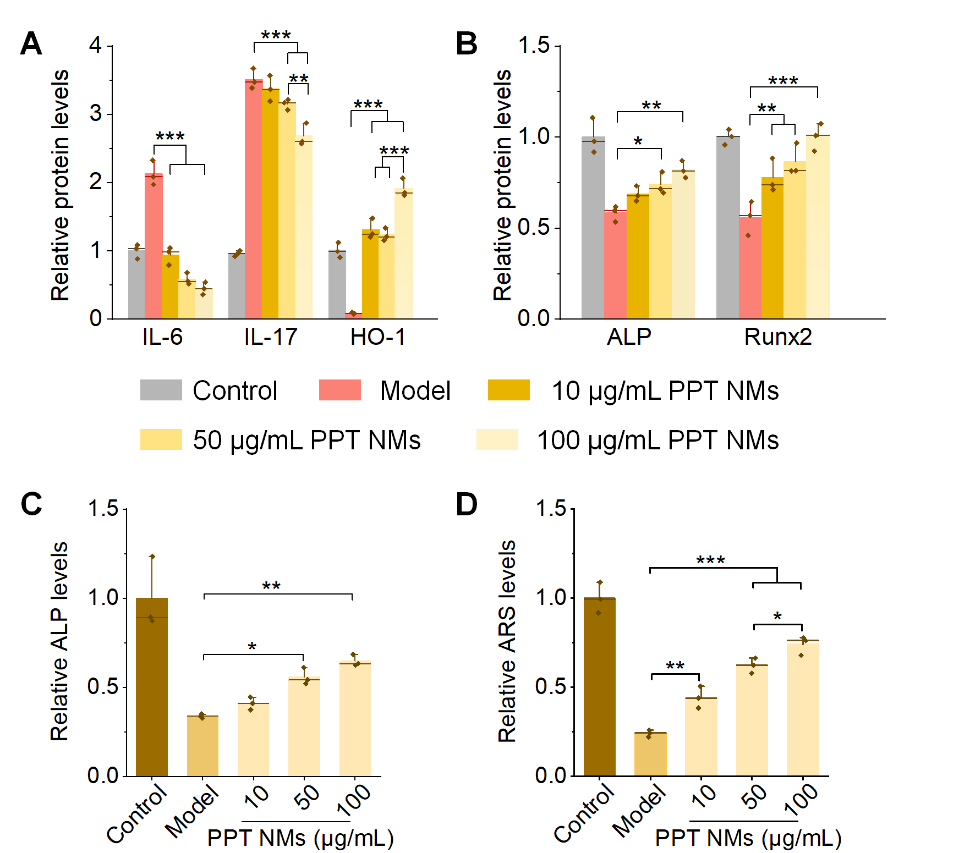


**Figure S7.** PPT NMs attenuate inflammation and promote osteogenic differentiation of hPDLSCs under chronic inflammatory conditions. (A-B) WB quantification of protein levels of IL-6, IL-17, HO-1, ALP, and Runx2 at day 7 after different treatments. (C-D) Quantitative analysis of the ALP levels (C) and ARS levels (D) after different treatments at days 7 and 21, respectively. Data are means ± SD (n = 3). *P < 0.05, **P < 0.01, and ***P < 0.001.

**
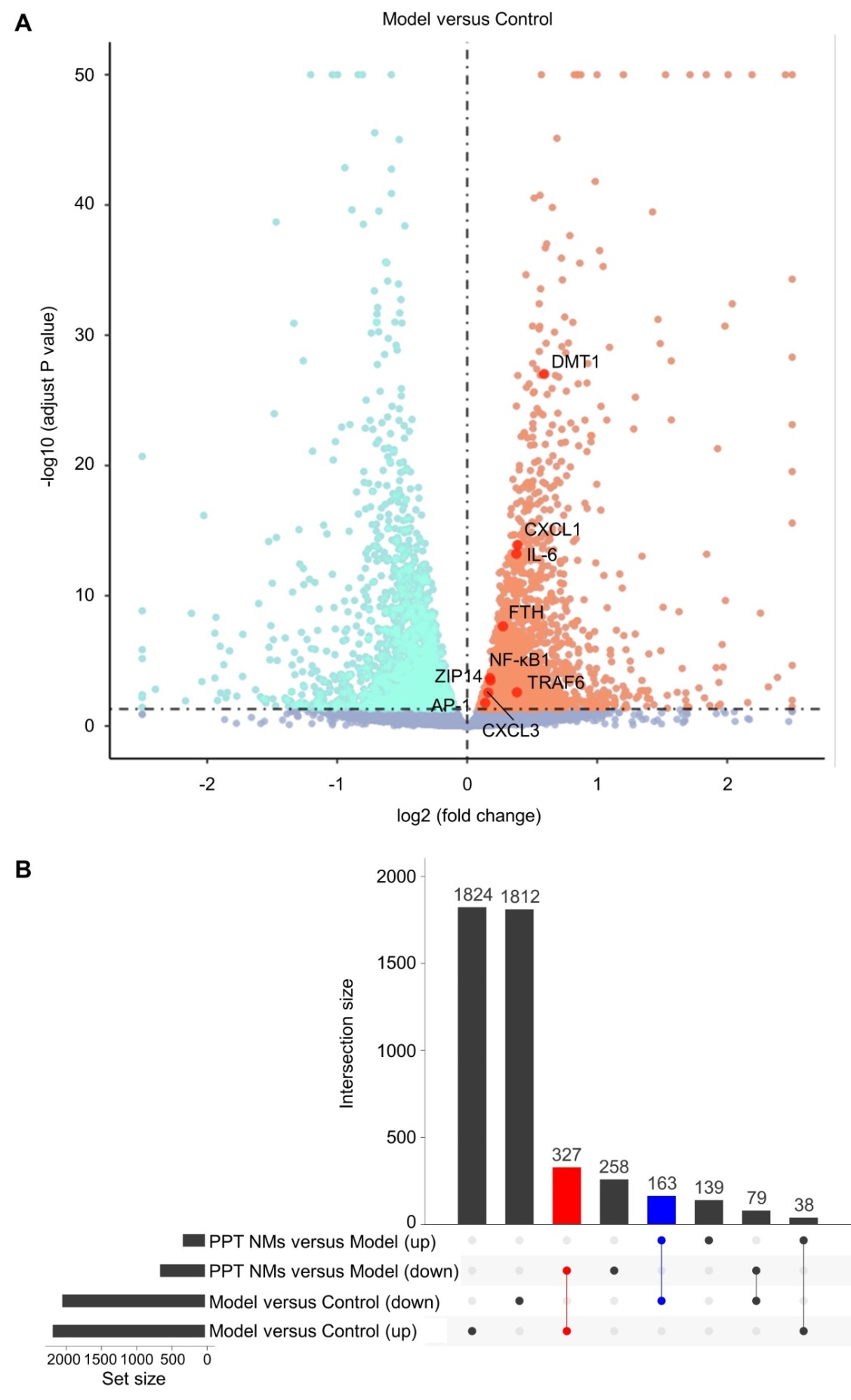
**

**Figure S8.** RNA-seq analysis of hPDLSCs after different treatments. (A) The volcano plot of RNA-seq results illustrates the expressions of up-regulated genes (orange dots) and down-regulated genes (green dots) in the control and model groups. (B) The upset plot illustrates the DEGs and their overlaps between different experimental groups.


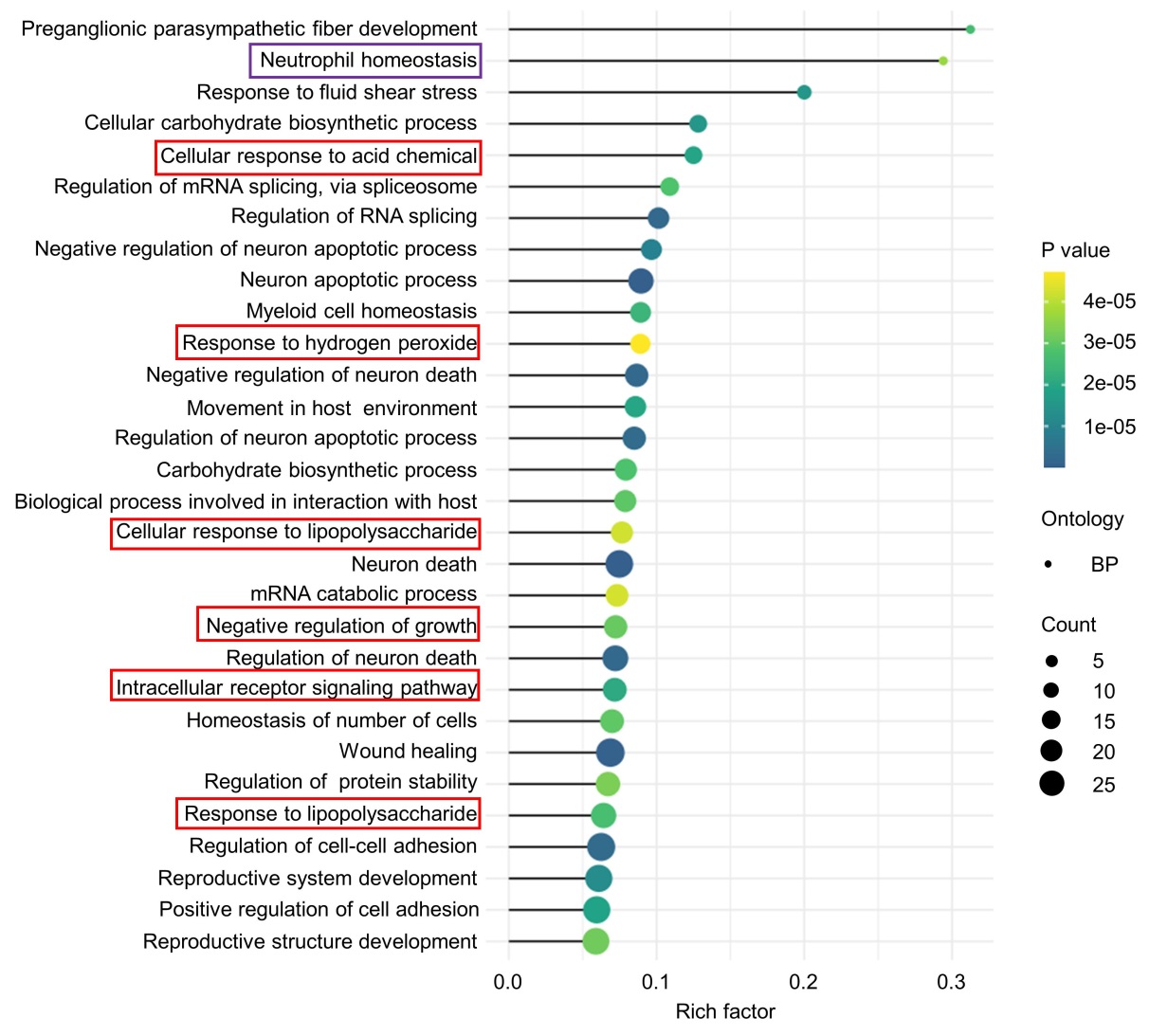


**Figure S9.** GO analysis of DEGs in hPDLSCs identified by RNA-seq. The purple box indicates immune regulation, while red boxes show inflammatory responses. BP, biological process.

**
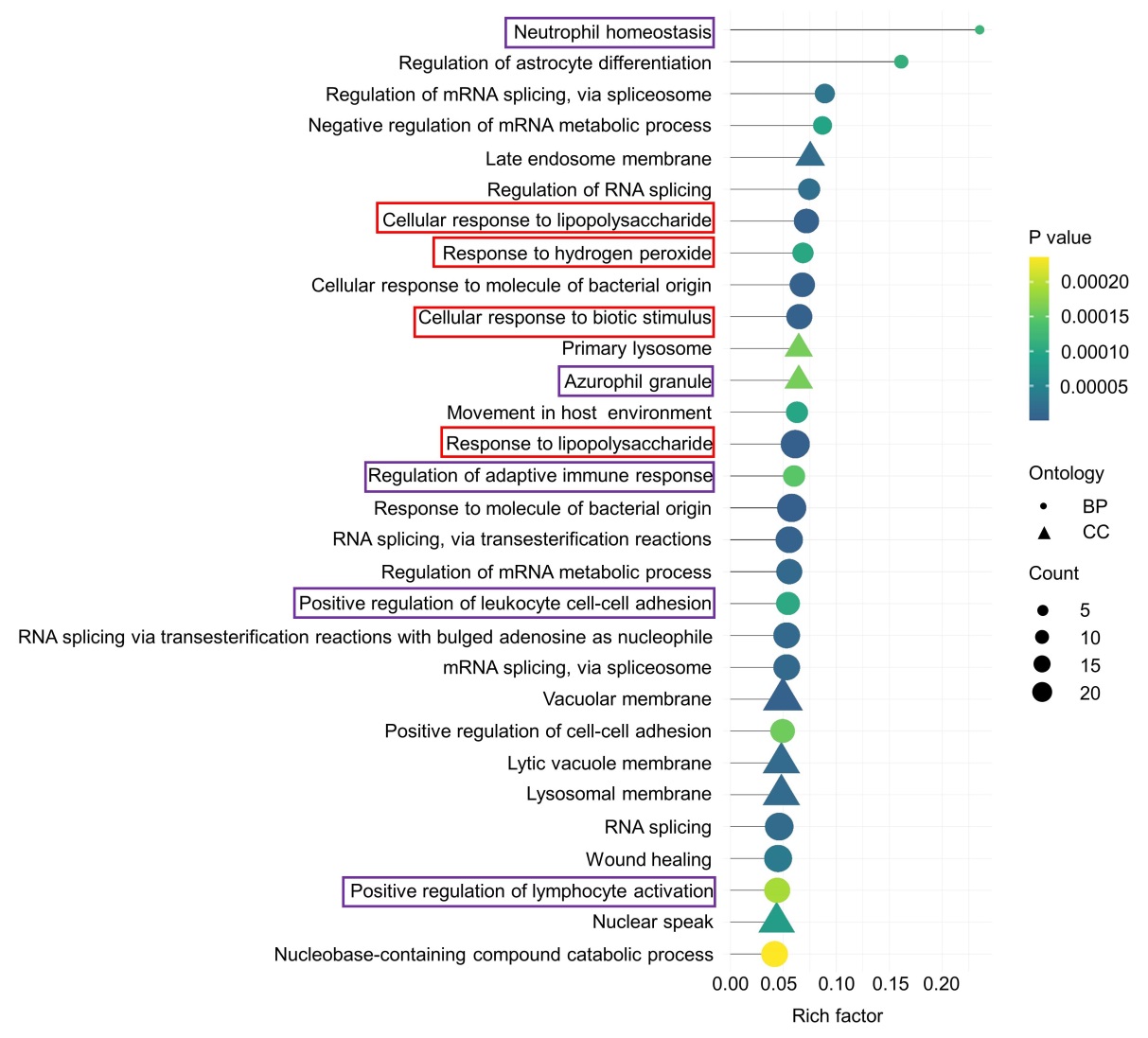
**

**Figure S10.** GO analysis of co-expressed DEGs between up-regulated genes in the model group and down-regulated ones in the PPT NMs group in hPDLSCs. The purple boxes denote immune regulations, while the red boxes show inflammatory responses. BP, biological process; CC, cellular component.

**
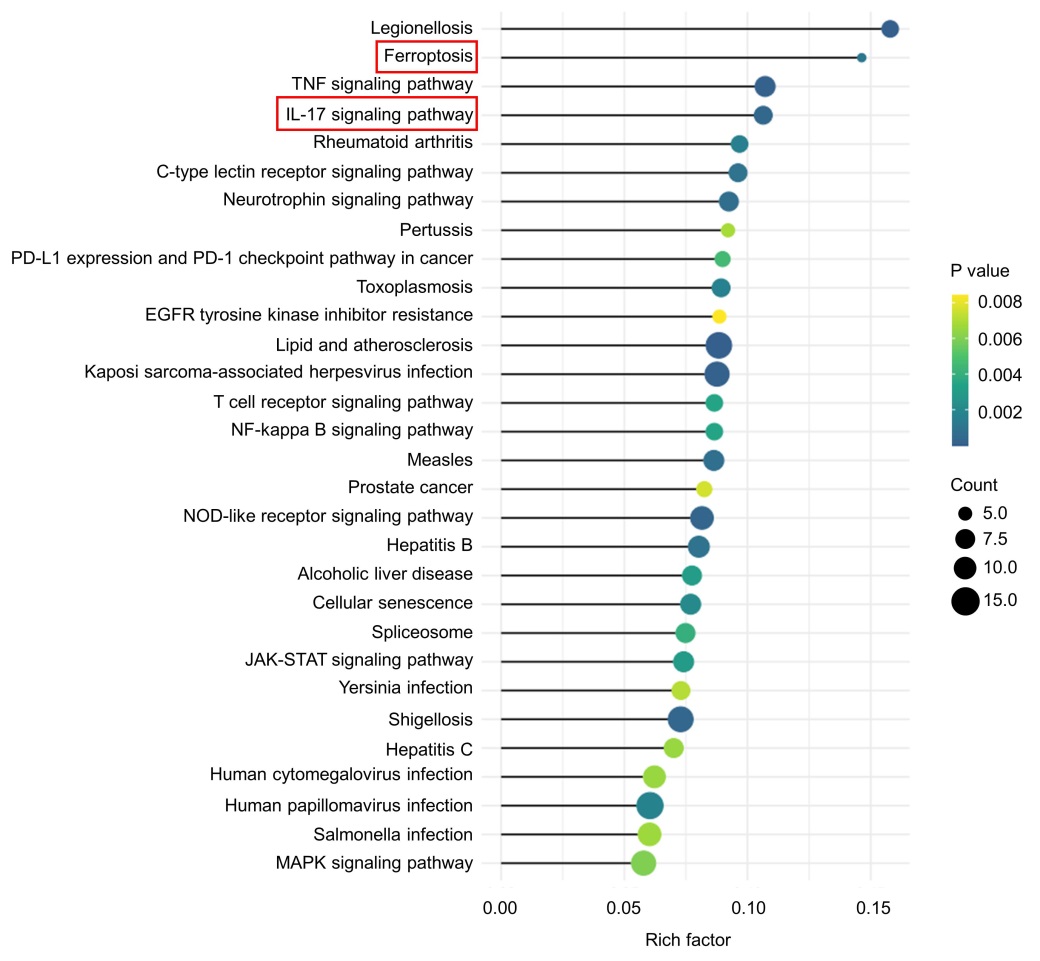
**

**Figure S11.** KEGG pathway enrichment of DEGs identified from RNA-seq.


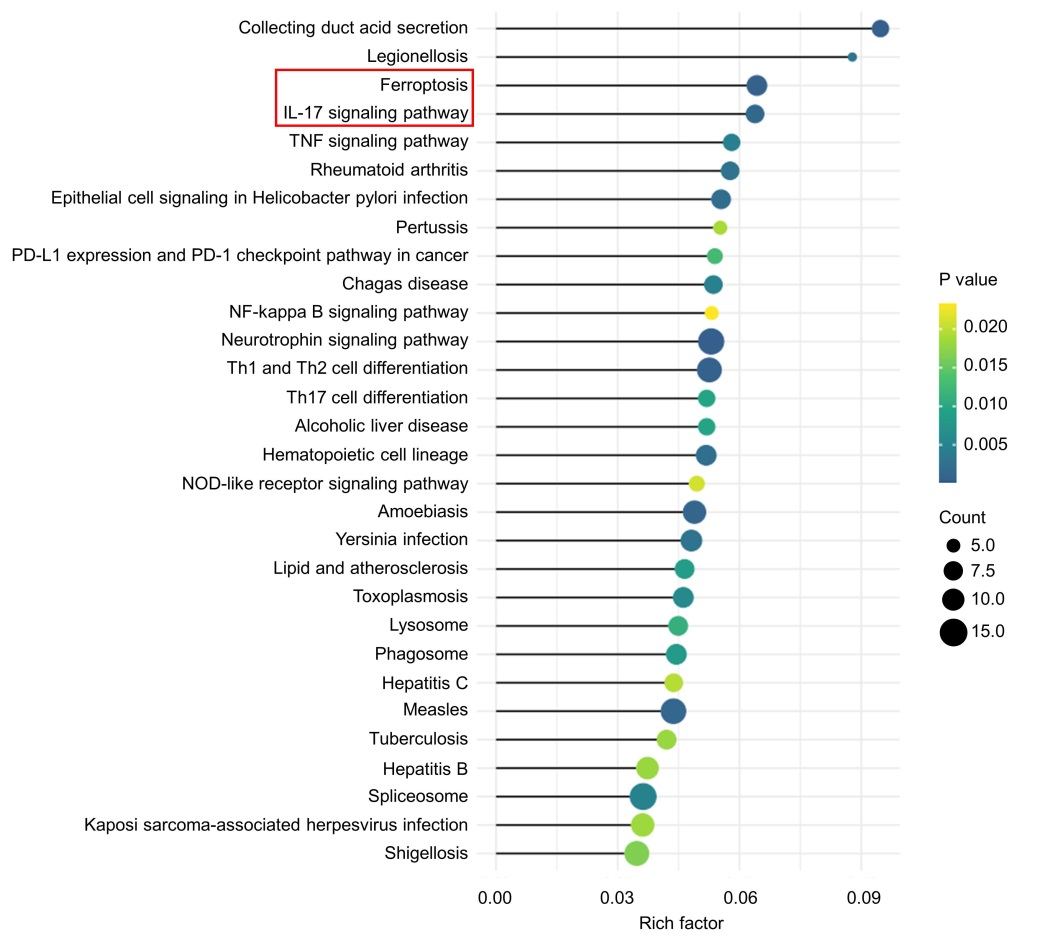


**Figure S12.** KEGG pathway enrichment of the co-expressed DEGs in hPDLSCs between the up-regulated genes in the model group (compared to the control) and the down-regulated ones in the PPT NMs group (compare to the model).


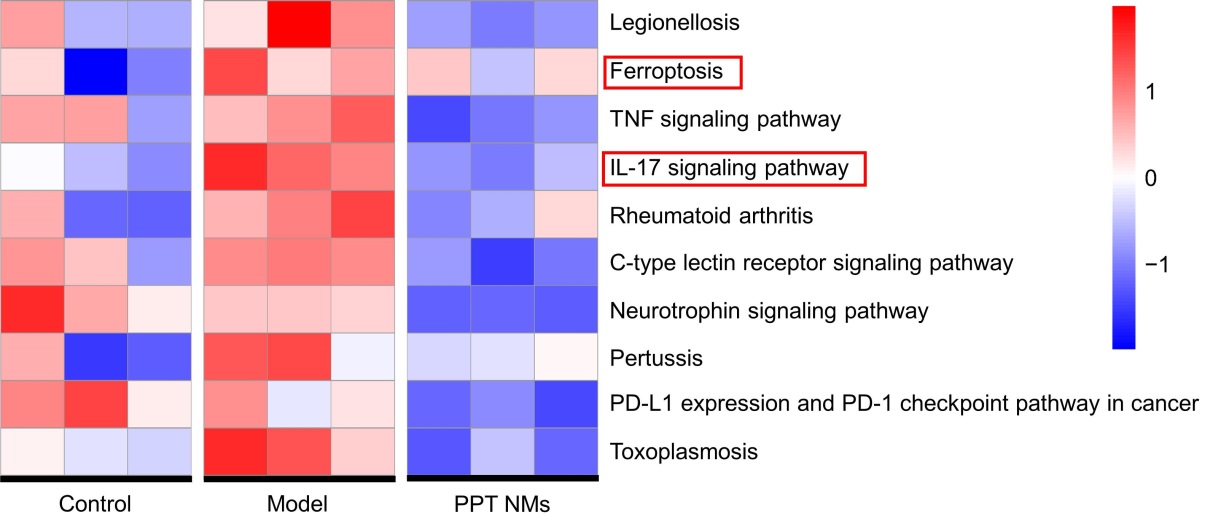


**Figure S13.** GSVA of KEGG shows the top 10 pathways.


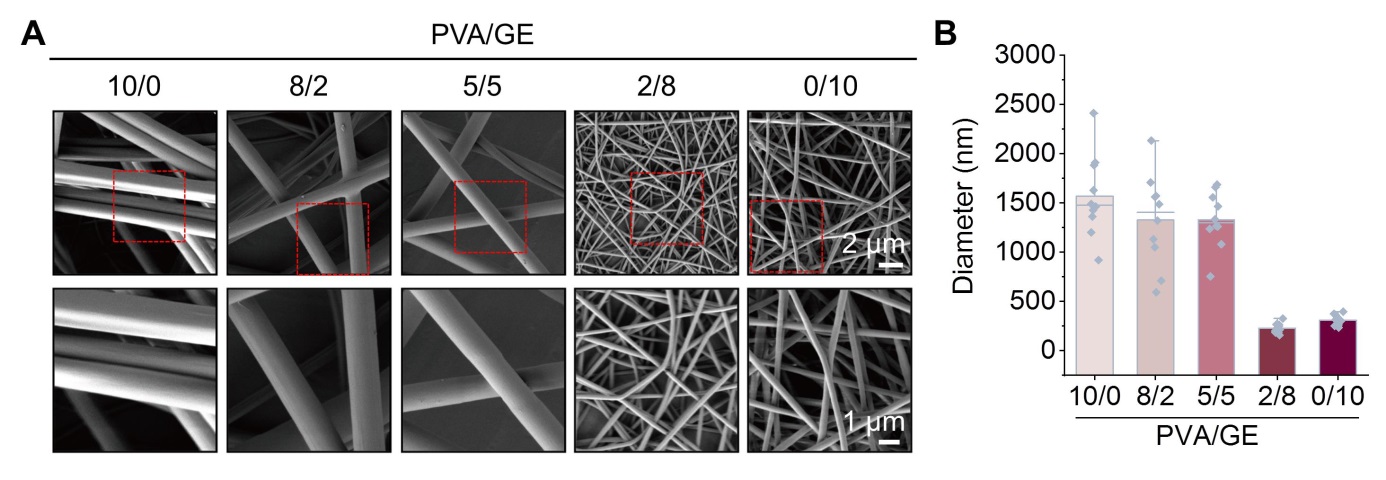


**Figure S14.** Characterization of PVA/GE nanofibers via SEM. (A-B) SEM images (A) and the quantified diameter (B) of nanofibers produced with different weight ratios of PVA/GE. Data in (B) are presented as means ± SD (n = 10).

**
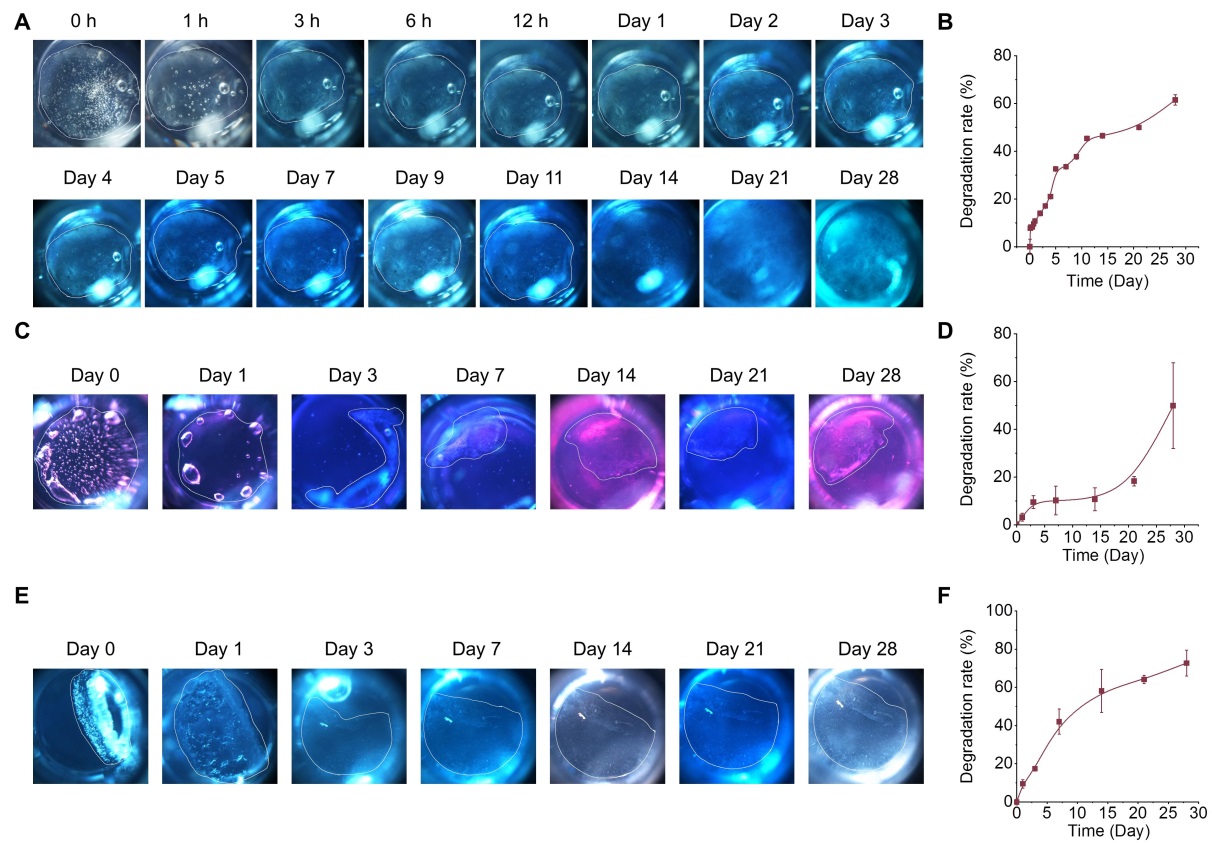
**

**Figure S15.** In vitro hydrolysis profiles of P/G nanofibers in different media. (A-F) Typical digital photos (A, C, E) and degradation rates (B, D, F) of P/G nanofibers after incubation in PBS (A-B), α-MEM (C-D), or artificial saliva (E-F) for different time periods. Data in (B, D, and F) are presented as means ± SD (n = 4).


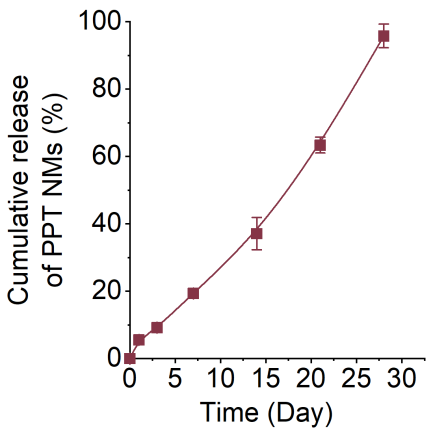


**Figure S16.** In vitro release profile of PPT NMs from P/G. Data are presented as means ± SD (n = 4).


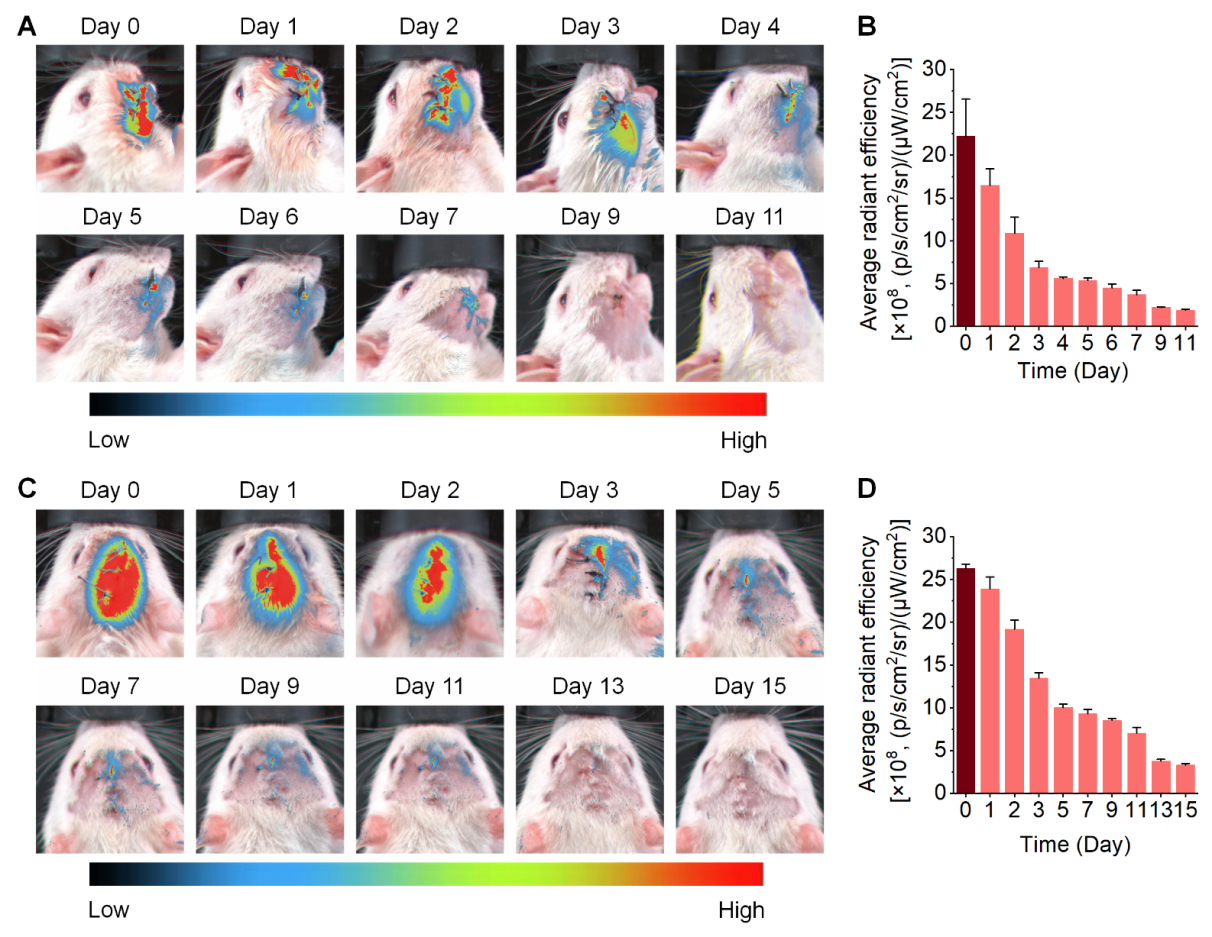


**Figure S17.** In vivo retention effects of the locally implanted bioactive patch. (A-B) In vivo time-lapse fluorescence images of mandible areas of rats after placing the patch containing Cy5-PPT NMs in the mandibular defect (A) and quantitative analysis of fluorescence intensities (B). (C-D) In vivo time-lapse fluorescence images of calvaria areas of rats after placing the patch containing Cy5-PPT NMs in the cranial defect (C) and quantitative analysis of fluorescence intensities (D). Data in (B, D) are presented as means ± SD (n = 3).


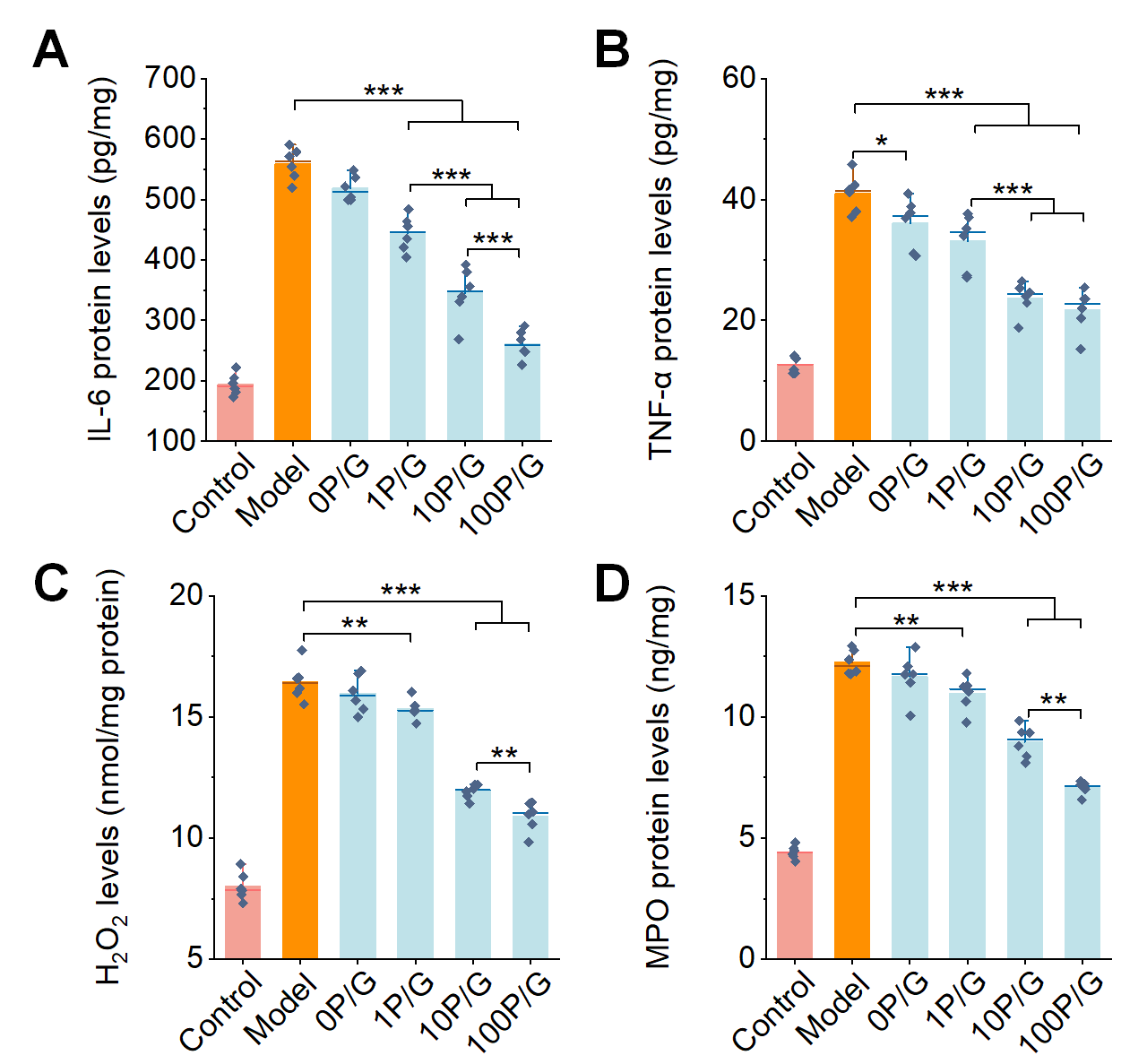


**Figure S18.** In vivo antioxidative and anti-inflammatory effects of PPT NMs in rats with mandibular defects. (A-D) Quantification of IL-6 (A), TNF-α (B), ROS (C), and MPO (D) in the mandibular defects at day 3 after treatment with bioactive patches containing various contents of PPT NMs. Data are presented as means ± SD (n = 6). *P < 0.05, **P < 0.01, and ***P < 0.001.


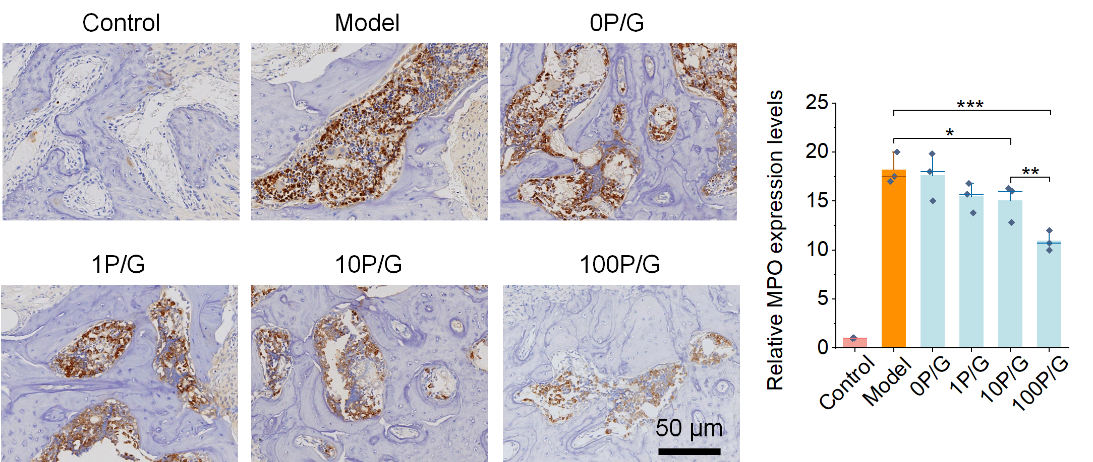


**Figure S19.** Representative microscopic images and quantitative analysis show the levels of MPO in mandibular defect tissue sections after different treatments. Data are presented as means ± SD (n = 3). *P < 0.05, **P < 0.01, and ***P < 0.001.

**
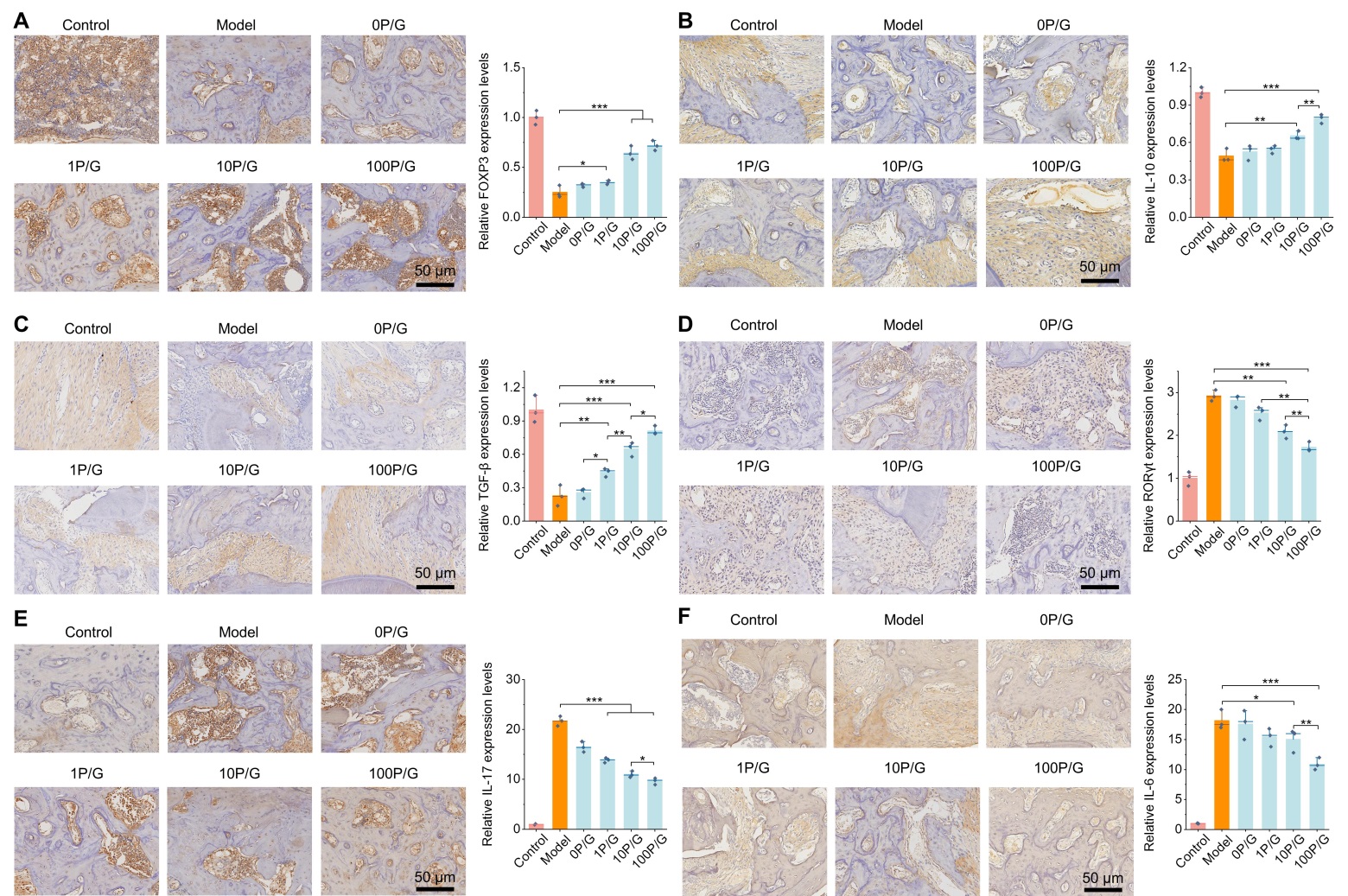
**

**Figure S20.** IHC analyses of the osteoimmunity-regulating effects of different bioactive patches in a rat model of mandibular defects at day 28. (A-F) Microscopic images and quantitative analysis indicate the expression levels of FOXP3 (A), IL-10 (B), TGF-β (C), RORγt (D), IL-17 (E), and IL-6 (F) in mandibular defect tissue sections after different treatments. Data are presented as means ± SD (n = 3). *P < 0.05, **P < 0.01, and ***P < 0.001.

**
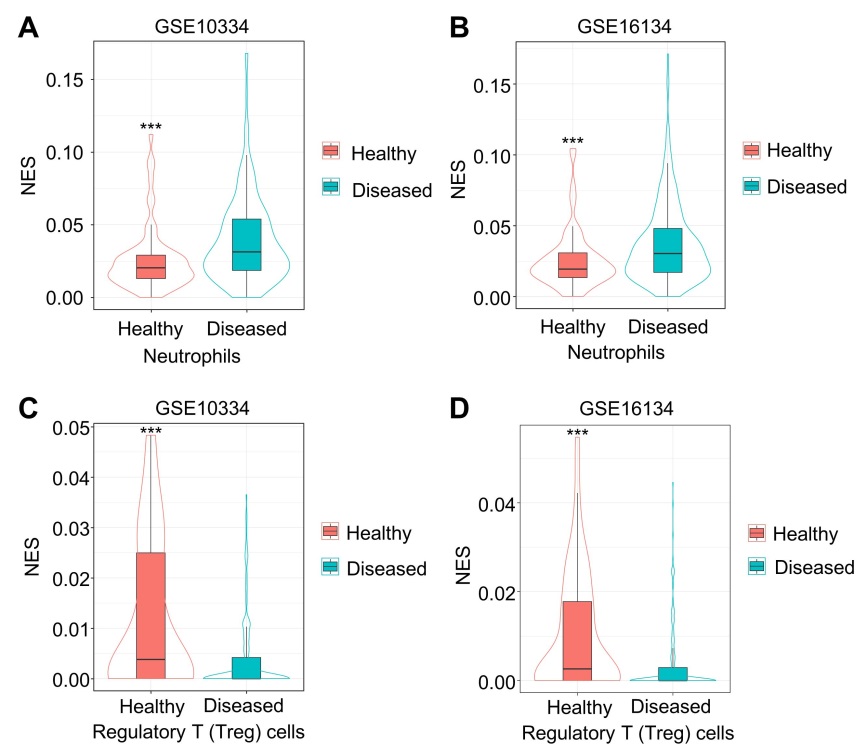
**

**Figure S21.** RNA-seq analysis of immune infiltration. (A-B) Relative levels neutrophils (A, B) and regulator T (Treg) cells (C, D) in the periodontal gingival tissues for healthy and diseased patients based on GSE10334 and GSE16134. ***P < 0.001.


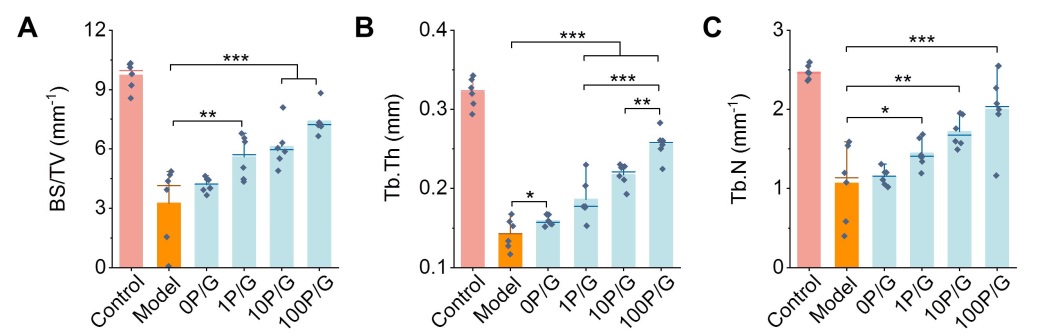


**Figure S22.** Bioactive patches promote osteogenesis in rats with mandibular defects. (A-C) Quantification of BS/TV (A), Tb.Th (B), and Tb.N (C) in mandibular defect areas. Data are presented as means ± SD (n = 6). *P < 0.05, **P < 0.01, and ***P < 0.001.

**
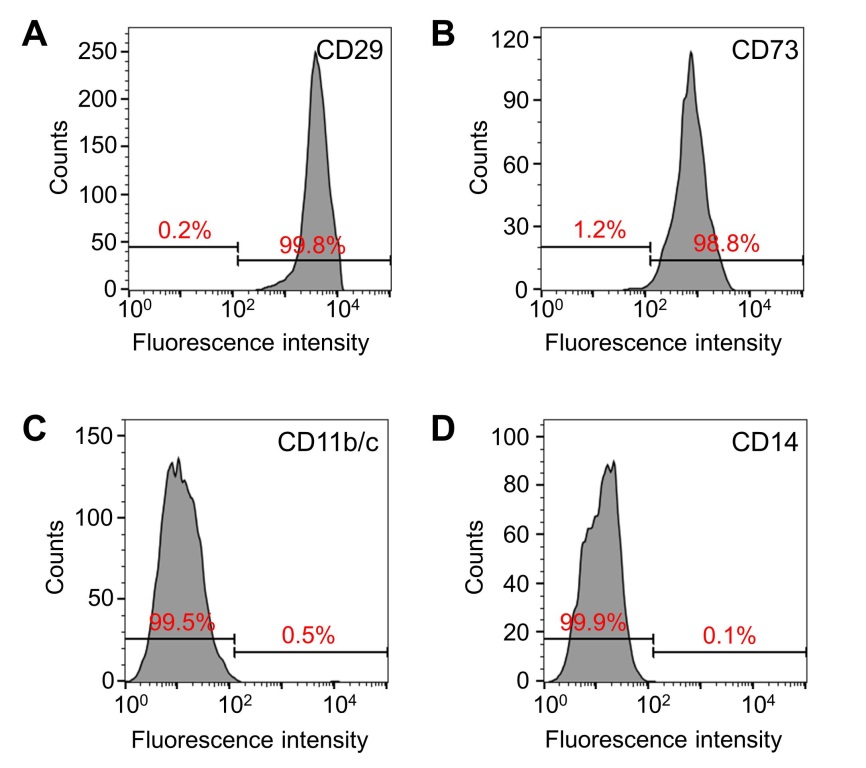
**

**Figure S23.** Flow cytometry identification of rPDLSCs. (A-D) Flow cytometric quantification illustrates the high expression of surface markers of CD29 (A) and CD73 (B) as well as the low expression of CD11b/c (C) and CD14 (D) on isolated rPDLSCs.


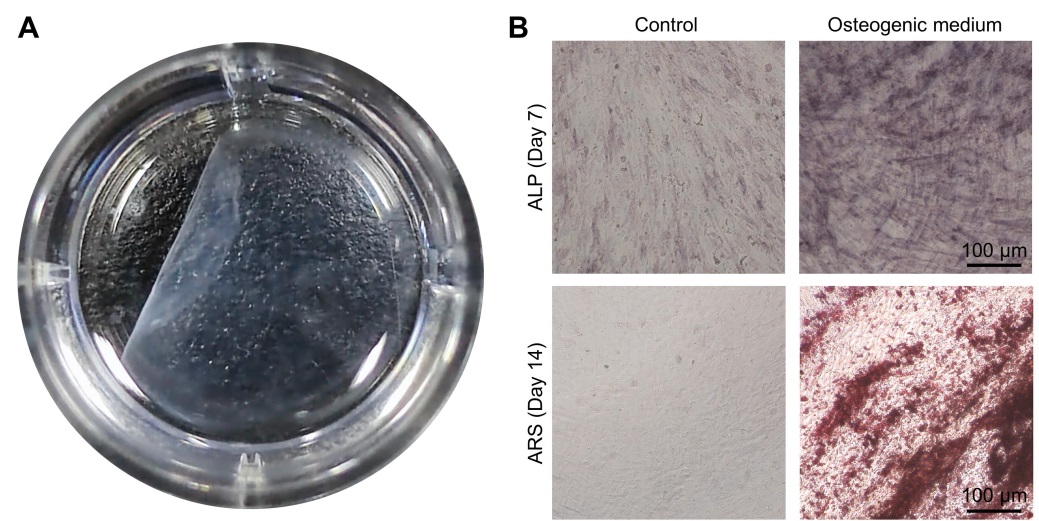


**Figure S24.** Preparation and evaluation of the rPDLSCs sheet. (A) Digital photos of a stem cell sheet of rPDLSCs. (B) The stem cell sheets stained with ALP or ARS with or without osteogenic induction at days 7 and 14, respectively.


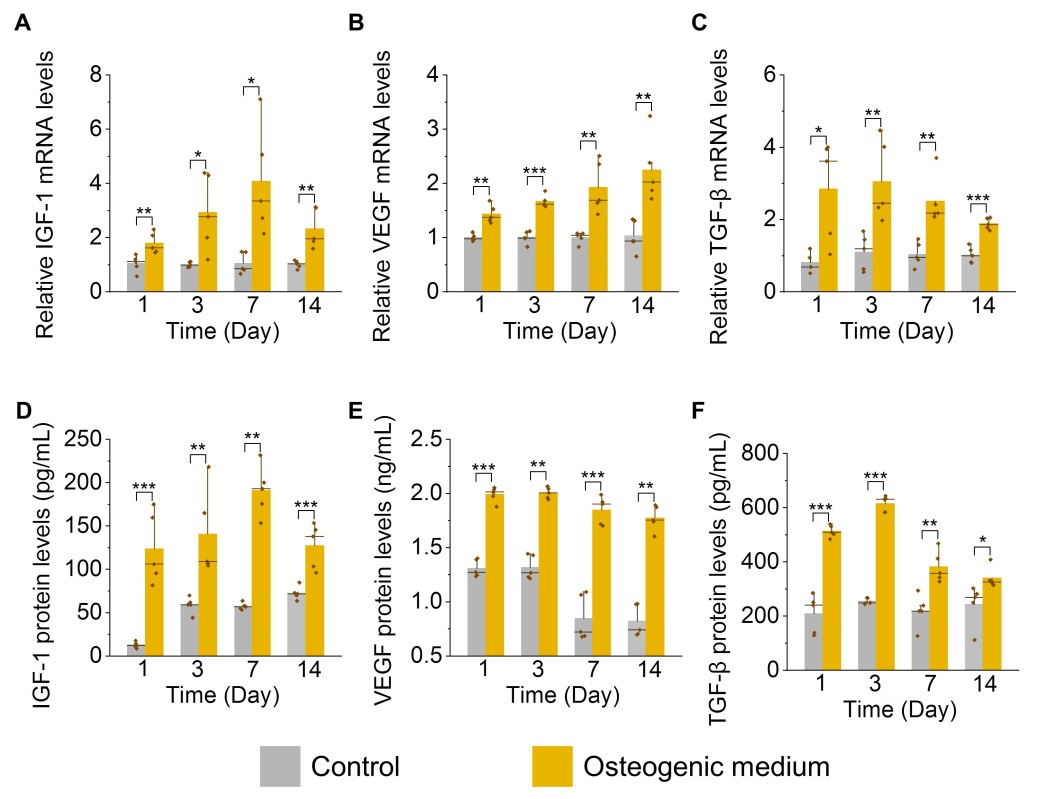


**Figure S25**. The paracrine effects of the rPDLSCs sheet under osteogenic induction. (A-C) mRNA levels of IGF-1 (A), VEGF (B), and TGF-β (C). (D-F) Protein levels of IGF1 (D), VEGF (E), and TGF-β (F). In the control group, the cell sheets were incubated in the regular medium, while cell sheets in the other group were incubated in the osteogenic medium. Data are means ± SD (n = 5). *P < 0.05, **P < 0.01, and ***P < 0.001.

**
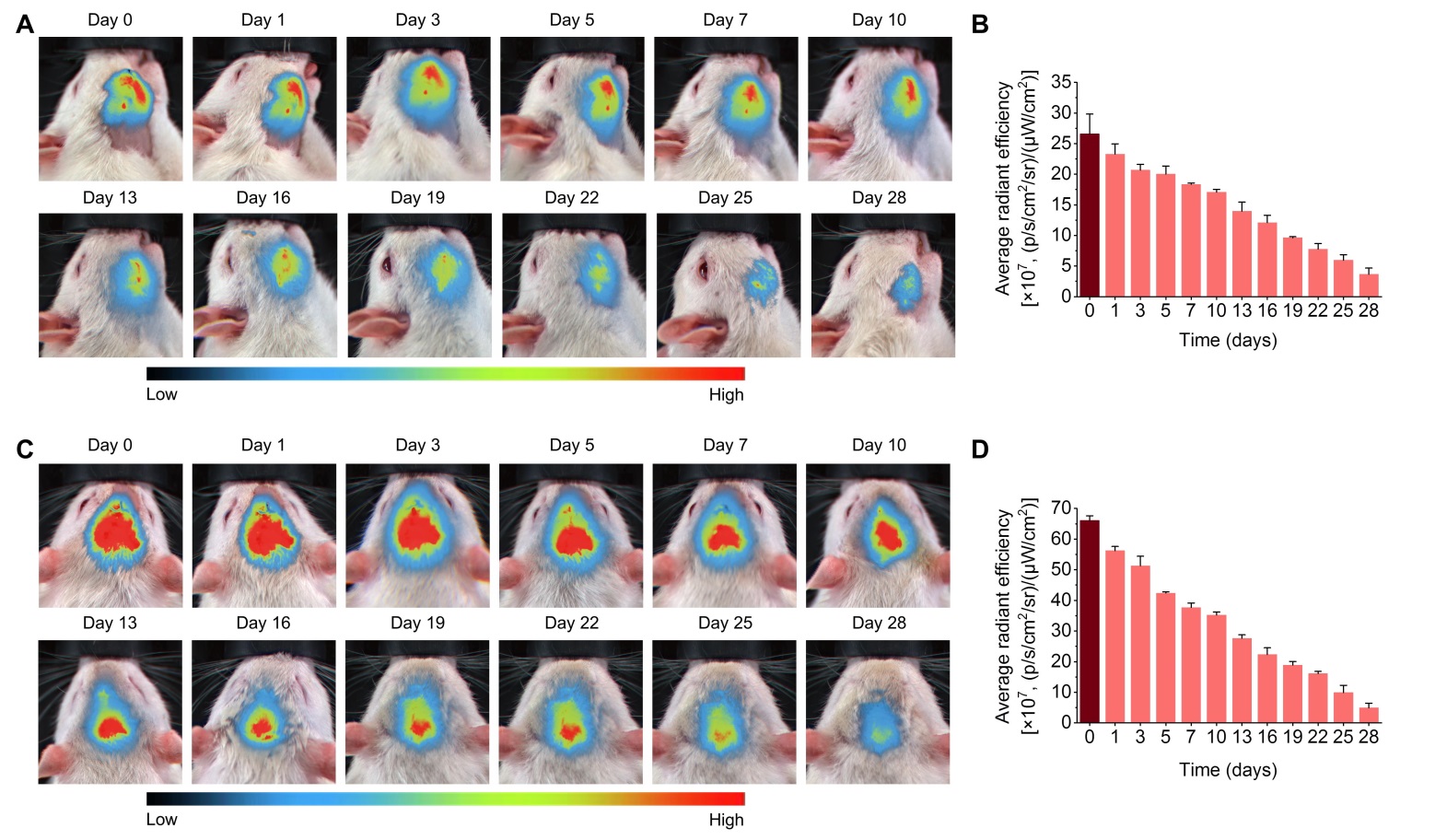
**

**Figure S26.** In vivo retention effects of the locally implanted cell sheets of rPDLSCs. (A-B) In vivo time-lapse fluorescence images of mandible areas of rats after planting the cell sheets in the mandibular defect (A) and quantitative analysis of fluorescence intensities (B). (C-D) In vivo time-lapse fluorescence images of calvaria areas of rats after planting the cell sheets in the cranial defect (C) and quantitative analysis of fluorescence intensities (D). Data in (B, D) are presented as means ± SD (n = 3).
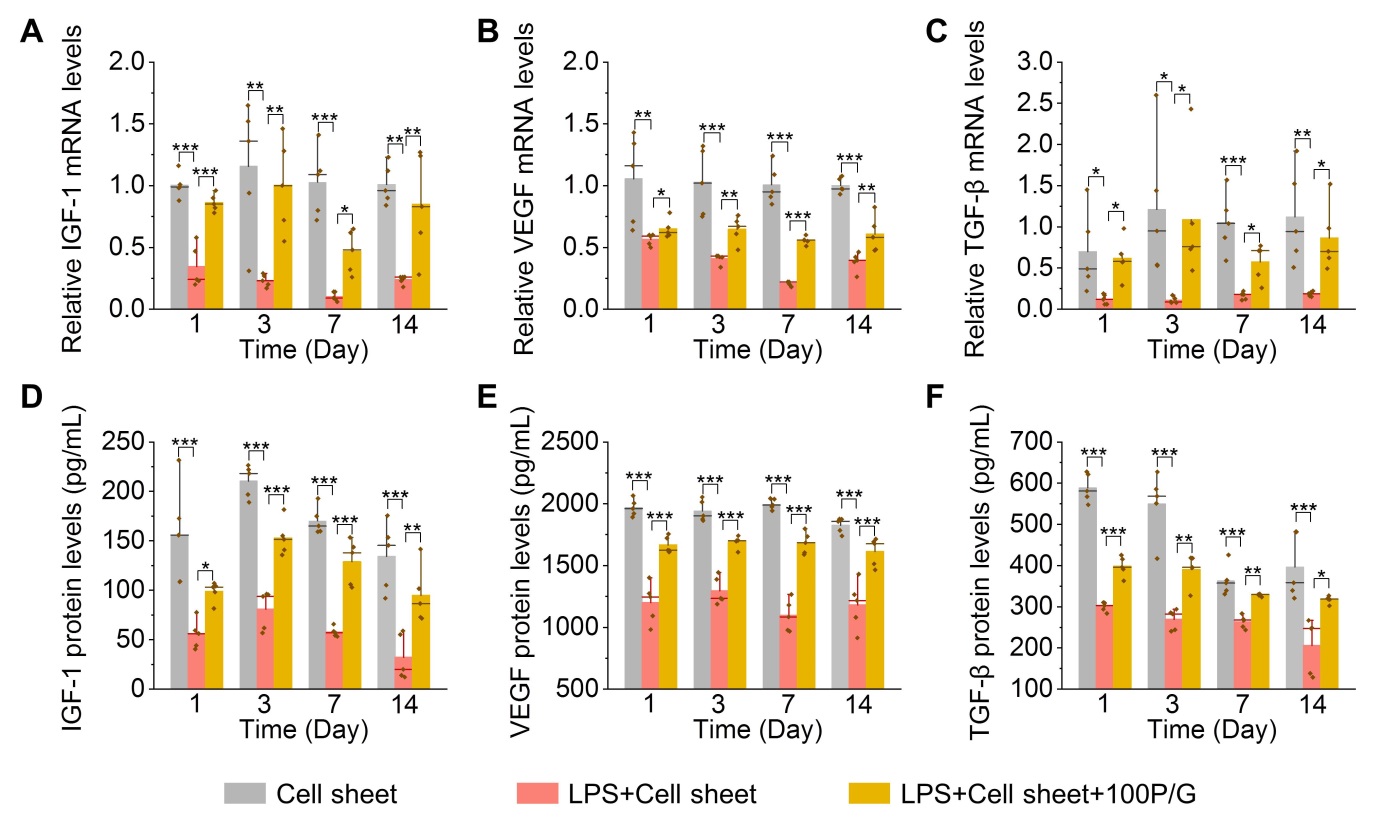


**Figure S27**. The effects of C100P/G on paracrine actions of the rPDLSCs sheet in the presence of LPS-mediated chronic inflammation. (A-C) mRNA levels of IGF-1 (A), VEGF (B), and TGF-β (C). (D-F) Protein levels of IGF-1 (D), VEGF (E), and TGF-β (F). In all cases, the cell sheets of rPDLSCs were cultured in the osteogenic medium for different time periods. In the control group, the cell sheets were treated with the osteogenic medium alone, while the model group was induced with 10 µg/mL LPS. For the 100P/G group, the cell sheets were treated with 10 µg/mL LPS and 100P/G. Data are presented as means ± SD (n = 5). *P < 0.05, **P < 0.01, and ***P < 0.001.


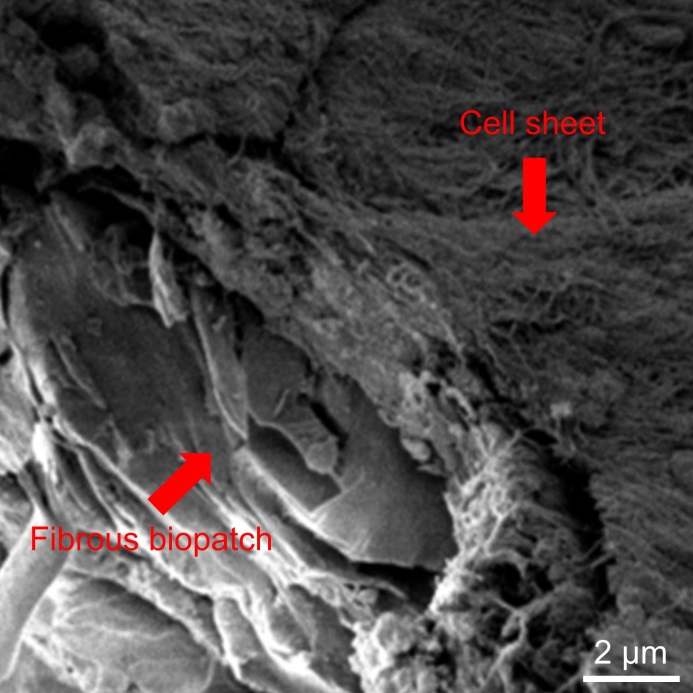


**Figure S28.** A typical SEM image illustrating the bilayer structure of the Janus biopatch.


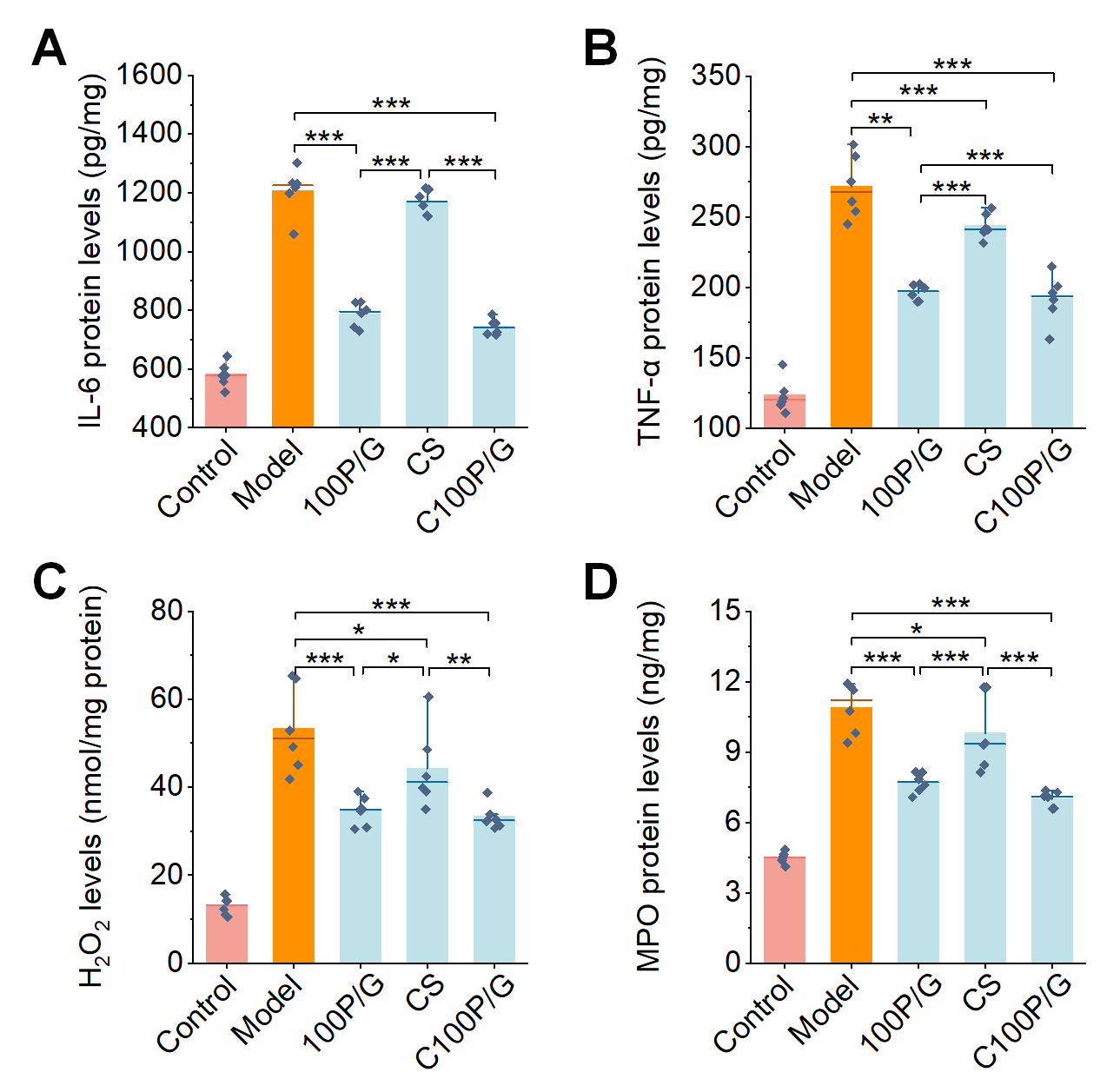


**Figure S29.** Antioxidative and anti-inflammatory effects of different patches. (A-D) Quantification of IL-6 (A), TNF-α (B), ROS (C), and MPO (D) in the mandibular defects at day 3 after treatment with 100P/G, CS, or their combination. Data are means ± SD (n = 6). *P < 0.05, **P < 0.01, and ***P < 0.001.


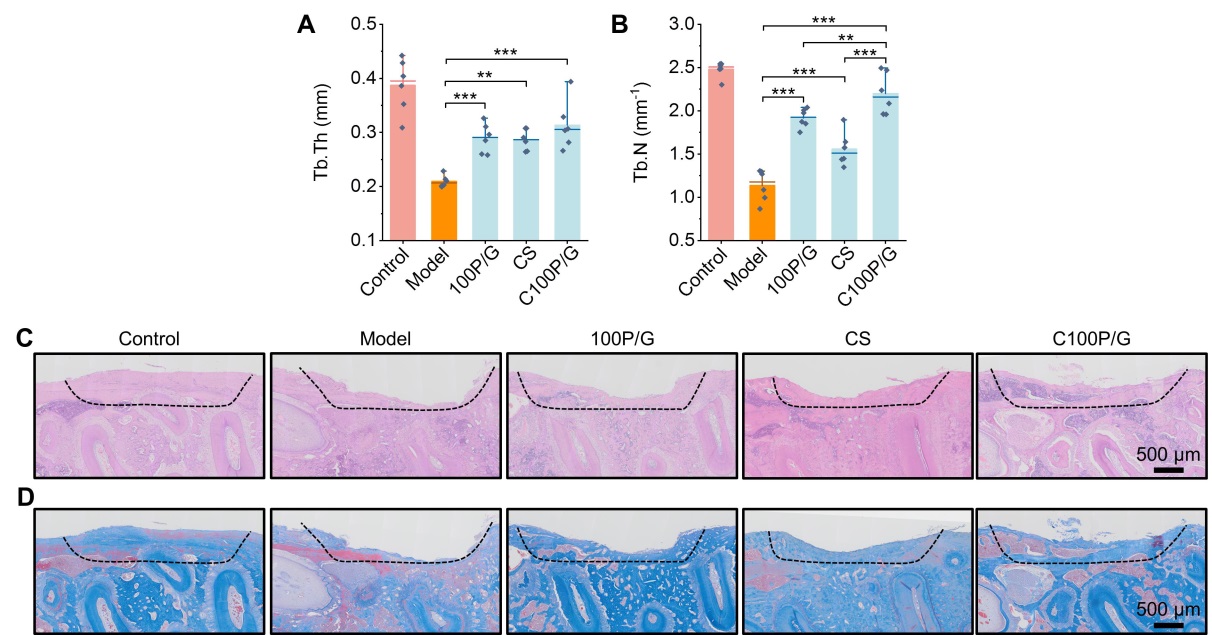


**Figure S30.** The bilayer patch promotes osteogenesis in rats with mandibular defects. (A-B) Quantification of Tb.Th (A) and Tb.N (B) in mandibular defect areas. (C-D) Microscopic images of defect tissue sections stained with H&E (C) or Masson (D). Data in (A, B) are means ± SD (n = 6). **P < 0.01, ***P < 0.001.

**
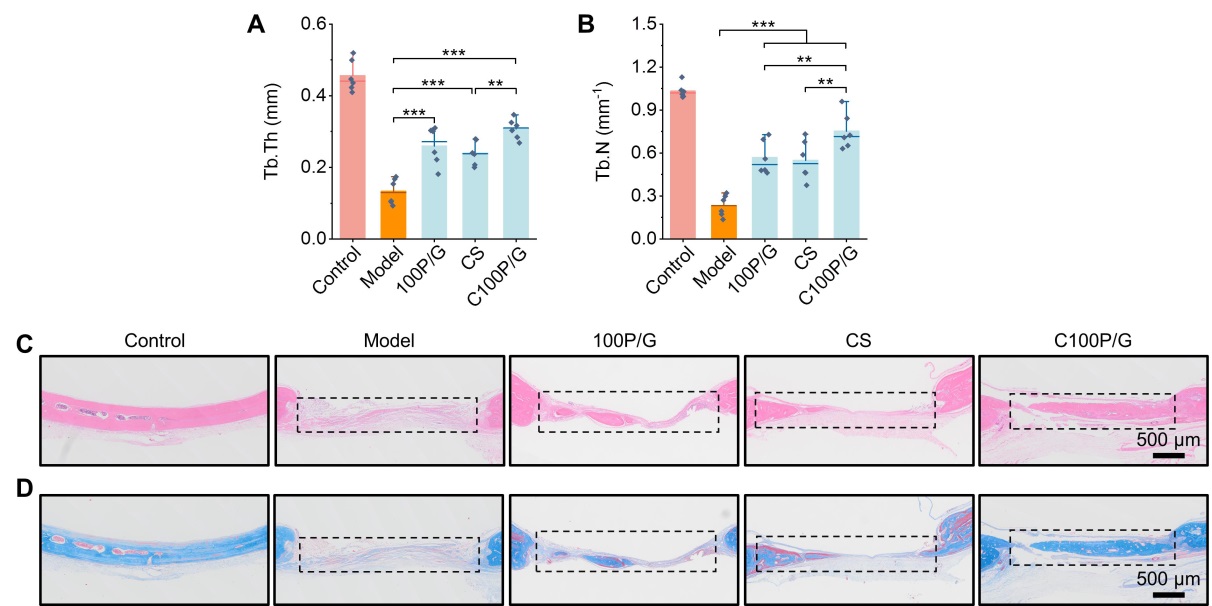
**

**Figure S31.** The bilayer patch promotes osteogenesis in rats with cranial defects. (A-B) Quantification of Tb.Th (A) and Tb.N (B) in cranial defect areas. (C-D) Microscopic images of defect tissue sections stained with H&E (C) or Masson (D). Data in (A, B) are presented as means ± SD (n = 6). **P < 0.01, ***P < 0.001.

**
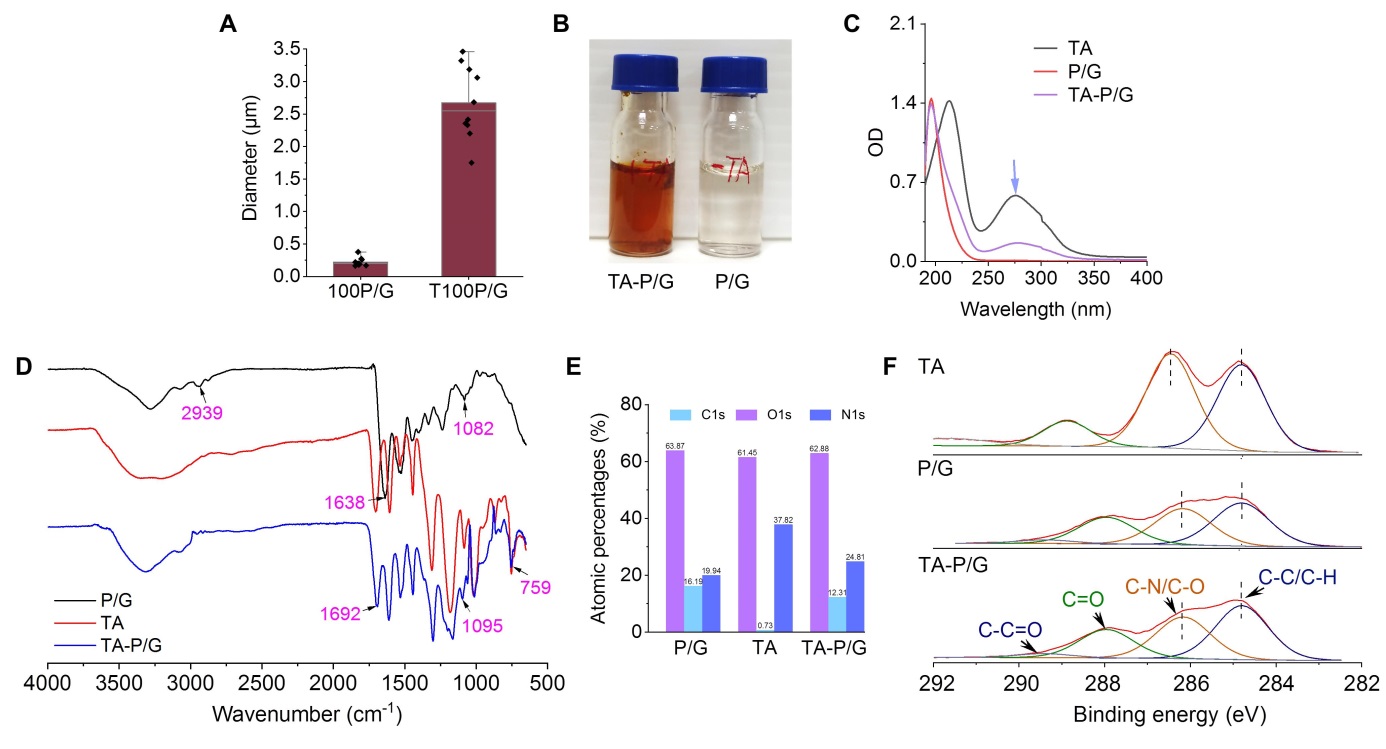
**

**Figure S32.** Characterization of the TA-functionalized bioactive patch. (A) The diameter of nanofibers with or without TA coating. 100P/G, the bioactive patch containing 100 µg PPT NMs; T100P/G, the TA-coated bioactive patch containing 100 µg PPT NMs. (B) Typical digital photos of TA-coated P/G or P/G after incubation with aqueous solution of AgNO_3_ at 37°C for 1 h. TA-P/G, the TA-coated P/G nanofibrous patch; P/G, the P/G nanofibrous patch. (C-D) UV-visible (C) and FTIR (D) spectra of P/G, TA, and TA-P/G. (E-F) XPS analysis of different samples. Data in (A) are presented as means ± SD (n = 10).


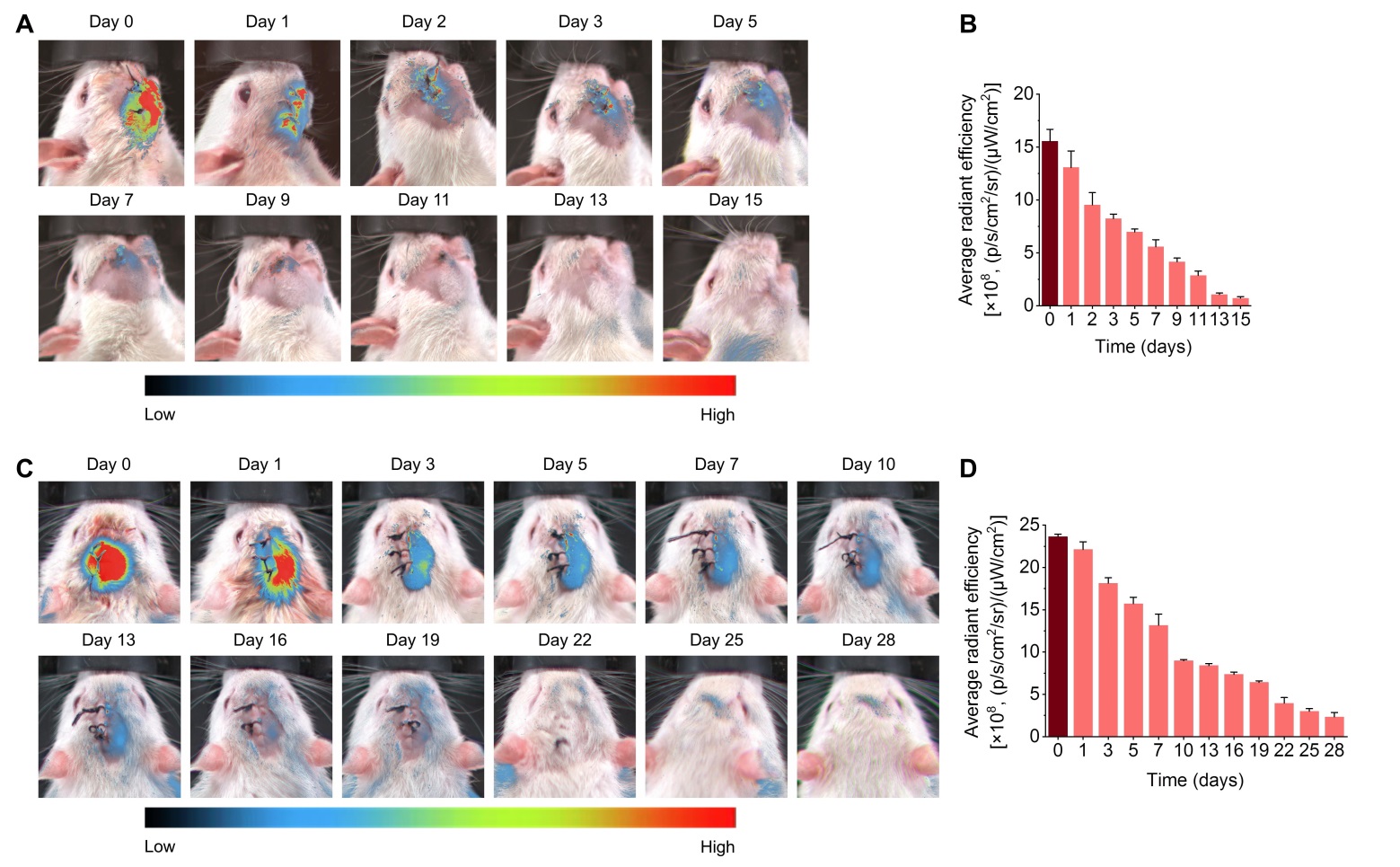


**Figure S33.** In vivo retention effects of the locally implanted TA-functionalized bioactive patch. (A-B) In vivo time-lapse fluorescence images of mandible areas of rats after placing the TA-functionalized patch containing Cy5-PPT NMs in the mandibular defect (A) and quantitative analysis of fluorescence intensities (B). (C-D) In vivo time-lapse fluorescence images of calvaria areas of rats after placing the TA-functionalized patch containing Cy5-PPT NMs in the cranial defect (C) and quantification of fluorescence intensities (D). Data are presented as means ± SD (n = 3).

**
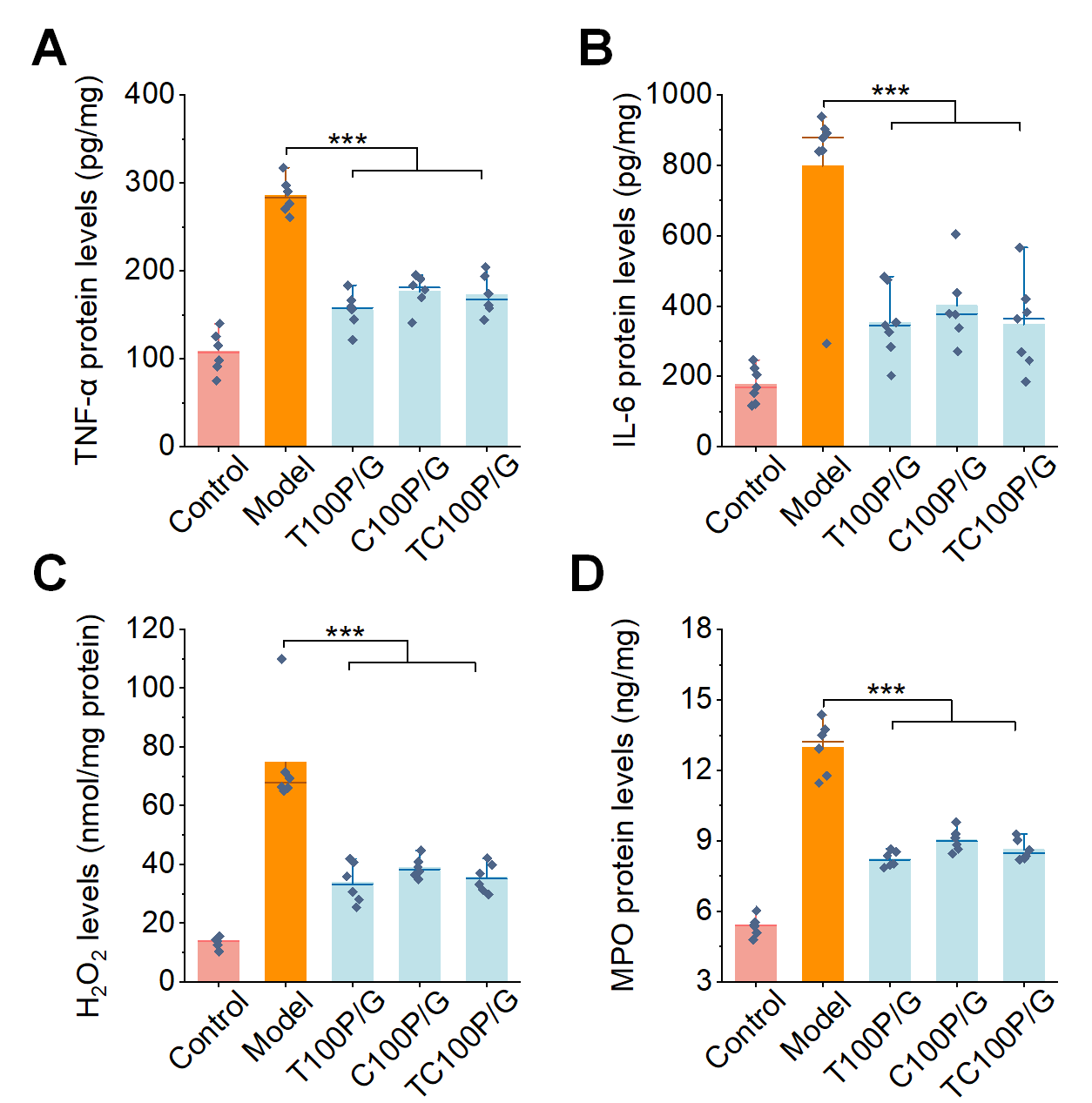
**

**Figure S34.** Anti-inflammatory and anti-oxidative effects of the bioadhensive Janus patch. (A-D) Quantification of IL-6 (A), TNF-α (B), ROS (C), and MPO (D) in the mandibular defects at day 3 after different treatments. Data are presented as means ± SD (n = 6). ***P < 0.001.


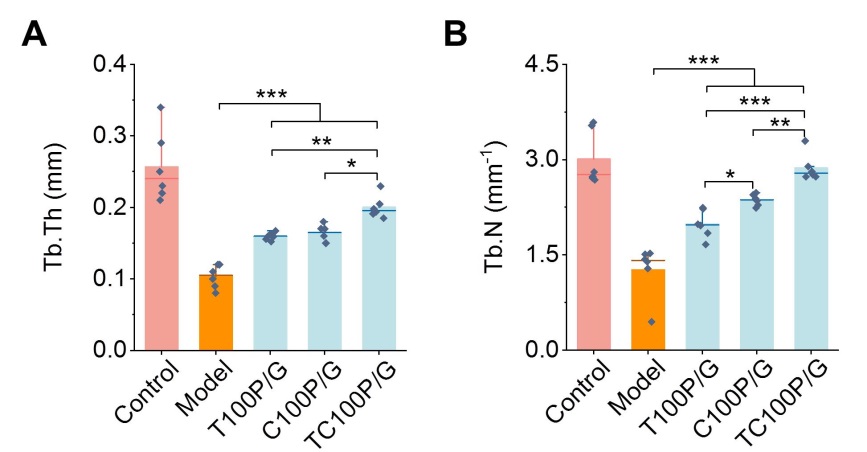


**Figure S35.** The bioadhensive Janus patch promotes osteogenesis in rats with mandibular defects. (A-B) Quantification of Tb.Th (A) and Tb.N (B) in mandibular defect areas. Data are presented as means ± SD (n = 6). *P < 0.05, **P < 0.01, and ***P < 0.001.


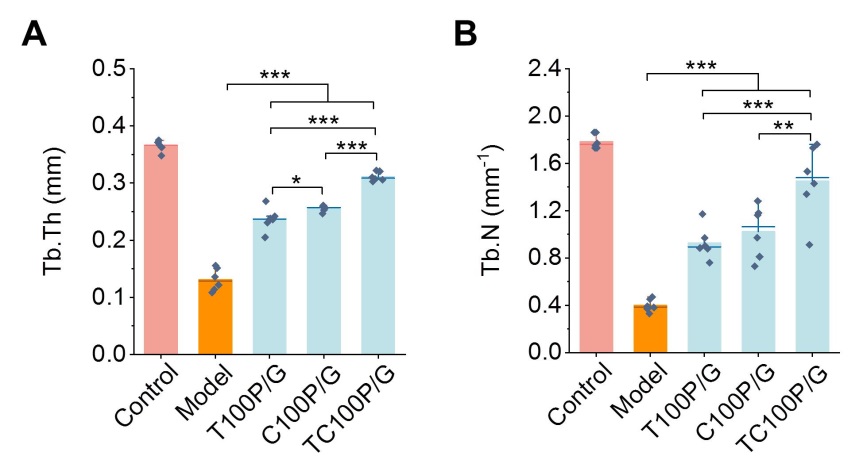


**Figure S36.** The bioadhensive Janus patch promotes osteogenesis in rats with cranial defects. (A-B) Quantification of Tb.Th (A) and Tb.N (B) in mandibular defect areas. Data are presented as means ± SD (n = 6). *P < 0.05, **P < 0.01, and ***P < 0.001.

**
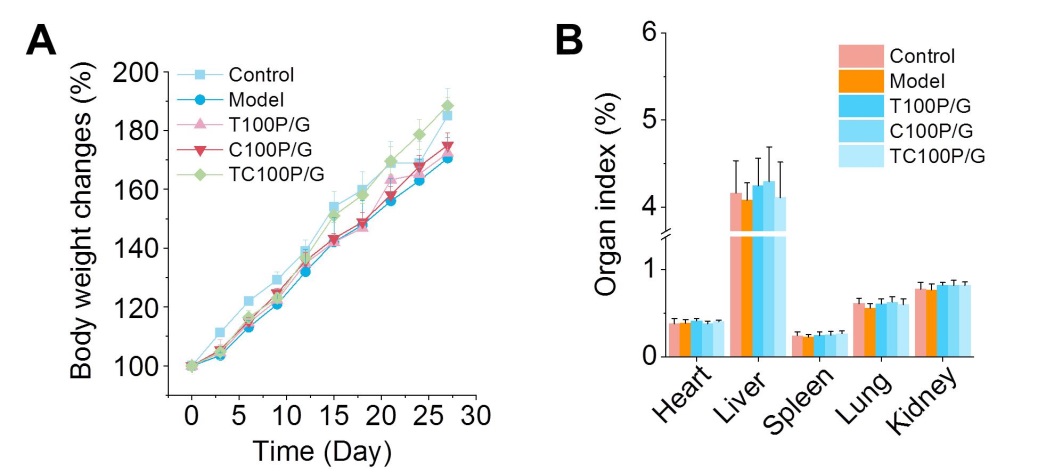
**

**Figure S37.** In vivo safety evaluation of T100P/G, C100P/G, and TC100P/G in rats. (A-B) Changes in the body weight (A) and the organ index of major organs (B) for rats in different groups. Data are presented as mean ± SD (n = 6).


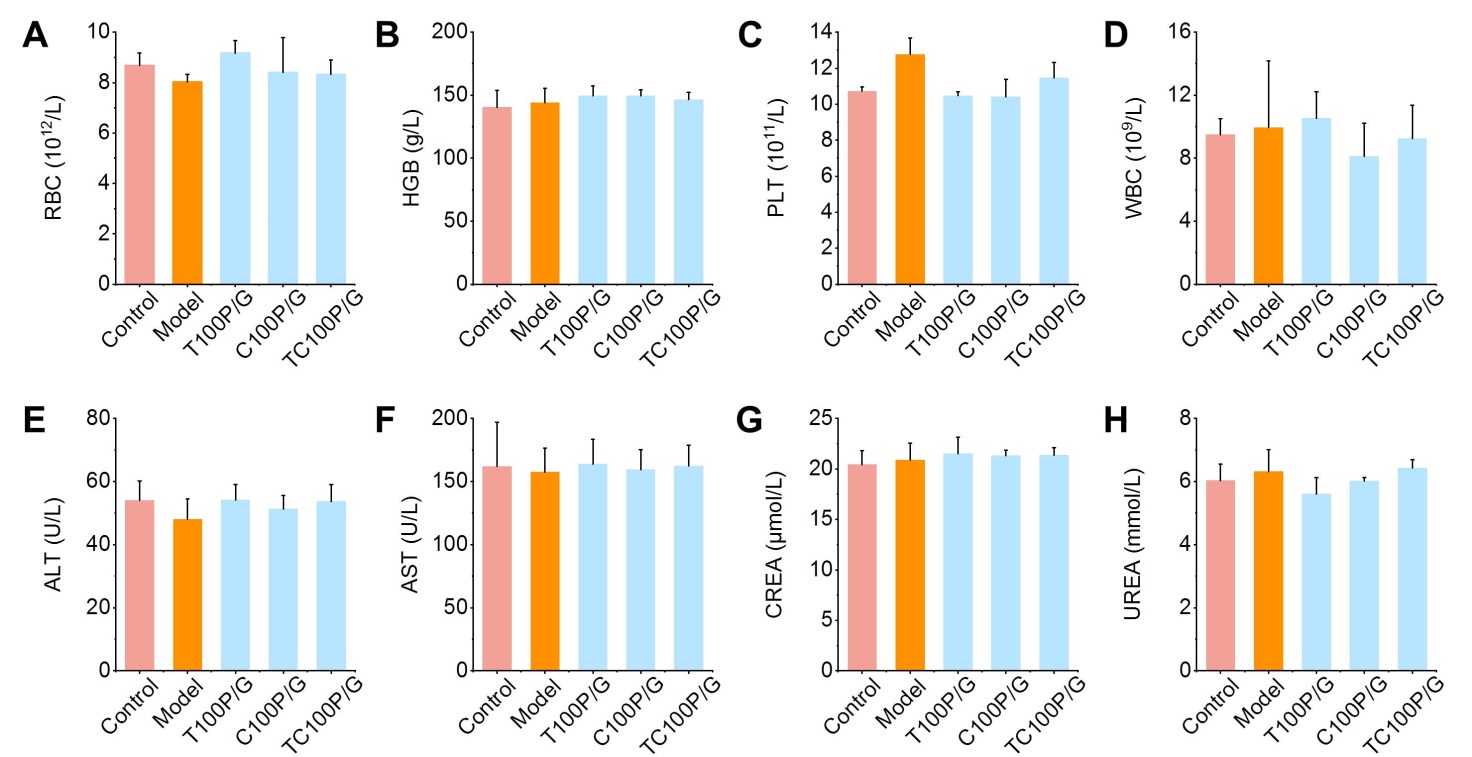


**Figure S38.** In vivo safety evaluations in rats after treatment with T100P/G, C100P/G, and TC100P/G. (A-D) Levels of typical hematological parameters of rats, including RBC (A), HGB (B), PLT (C), and WBC (D). (E-H) The serum levels of biochemical markers relevant to hepatic and kidney functions, including ALT (E), AST (F), CREA (G), and UREA (H). RBC, red blood cells; HGB, hemoglobin; PLT, platelets; WBC, white blood cells; ALT, alanine transaminase; AST, aspartate transaminase; CREA, creatinine; UREA, blood urea. Data are presented as mean ± SD (n = 6).


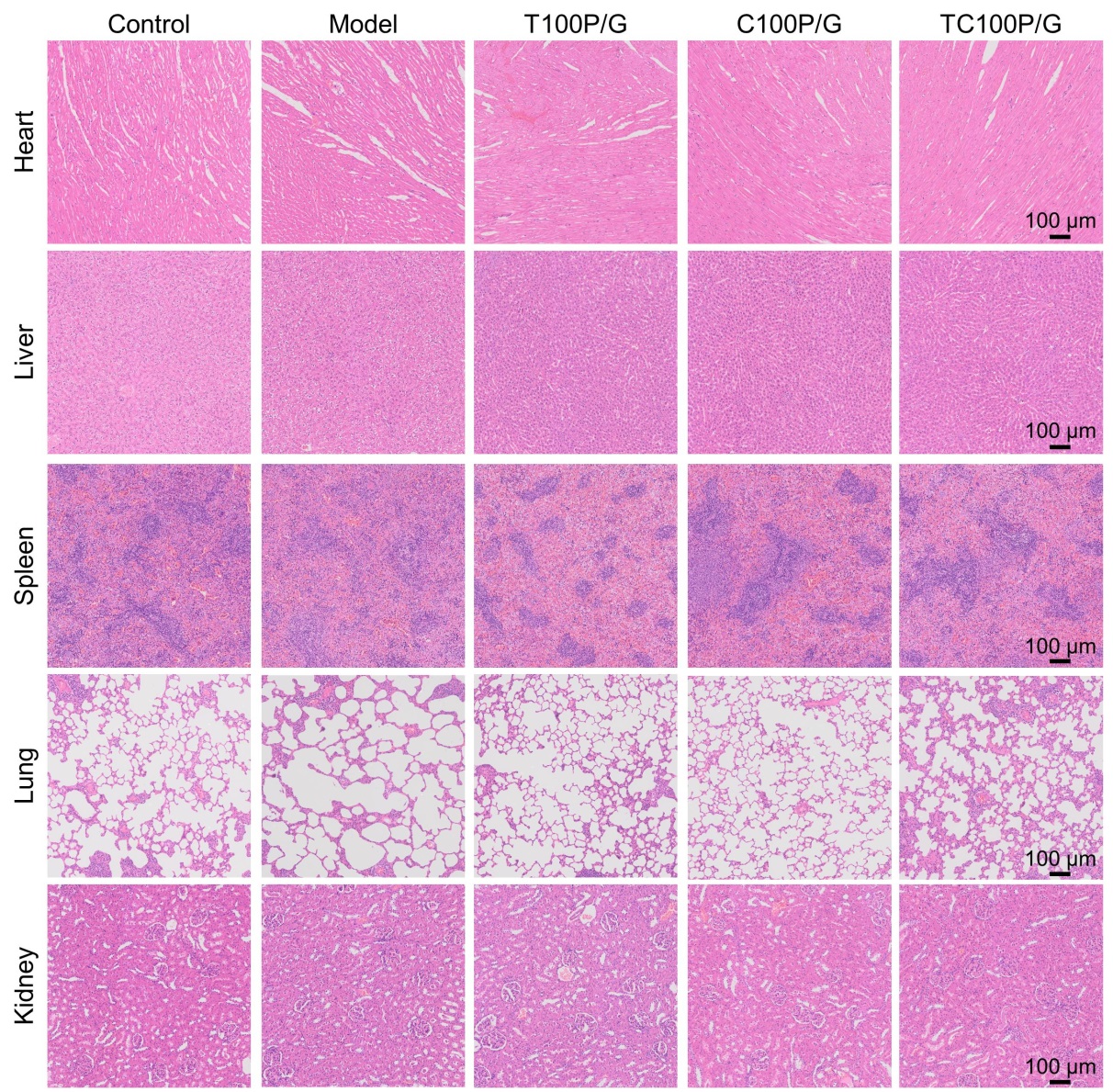


**Figure S39.** H&E-stained histological sections of major organs.
